# Towards energy-efficient massive MIMO-NOMA systems with sigma–delta ADCs and group SIC detection

**DOI:** 10.1038/s41598-026-49425-y

**Published:** 2026-05-01

**Authors:** Samar I. Farghaly, Mohamed M. Khafaga, Salah Khamis

**Affiliations:** Electronics and Electrical communications, Faculty of Engineering, Tanta University, Tanta, Egypt

**Keywords:** Massive MIMO, NOMA, Sigma–Delta ADCs, GSIC, Spectral efficiency, Energy efficiency, Engineering, Mathematics and computing

## Abstract

This paper investigates a massive MIMO–NOMA system in which the base station (BS) employs a large antenna array with low-resolution (1–2-bit) spatial Sigma–Delta (ΣΔ) ADCs to shape and suppress quantization noise. A linear minimum mean-squared error (LMMSE) estimator based on the Bussgang decomposition is developed to accurately acquire CSI under quantization. We derive asymptotic spectral efficiency (SE) expressions for group successive interference cancellation (GSIC) receivers over Rician and general fading channels, and establish a unified framework to compare linear, SIC, and GSIC detection schemes. Results show that, with GSIC receivers, transmitting power can be scaled inversely with the number of antennas, and system performance strongly depends on group size, ADC resolution, and antenna count. A low-complexity power-allocation scheme is proposed to satisfy quality-of-service (QoS) constraints with minimal transmitted power. Simulations confirm that 2-bit ΣΔ ADCs combined with MRC–GSIC receivers and a small number of groups achieve excellent SE and energy efficiency (EE), with negligible loss compared to full-resolution systems.

## Introduction

In next-generation wireless networks, integrating diverse services and applications requires wide-area connectivity, along with high spectral efficiency (SE) and energy efficiency (EE)^1–10^. Non-orthogonal multiple access (NOMA), designed to serve multiple user equipments (UEs) at the same time, is recognized for enhancing SE and EE under massive connectivity scenarios^20–30^. NOMA has already attracted strong interest from both academia and industry for beyond-5G and 6G communications^31–44^ and has been widely investigated in the literature for downlink transmission scenarios inspired by 3GPP Release 15^35–47^. At the same time, because of its favorable trade-off between SE and EE, massive multiple-input multiple-output (MIMO) technology with low-resolution analog-to-digital converters (ADCs) has emerged as a key candidate for next-generation wireless systems^8^. Low-resolution quantization is a major area of study for both the transmitter and receiver. For the uplink (precoding), using one-bit digital-to-analog converters (DACs) is a topic of emerging research. A common approach is to quantize the output of a conventional linear precoder, such as the zero-forcing (ZF) and maximum ratio transmission (MRT) precoders. The performance impact of this coarse quantization is often analyzed using the Bussgang decomposition^9–11^. More recent efforts emphasize directly designing optimal one-bit precoders; however, these designs face a major challenge in solving the resulting large-scale integer optimization problems.

### Related work

Many studies have focused on the spatial Sigma–Delta (ΣΔ) ADC architecture and its circuit implementations^12,13,48–51^. Previous work has shown that spatial ΣΔ ADCs in massive MIMO systems improve channel estimation accuracy and spectral efficiency, particularly for angularly confined users or spatially oversampled antenna arrays^14,50^. The uplink SE of a massive MIMO base station employing one-bit spatial ΣΔ quantization has been analyzed and compared with systems using infinite-resolution ADCs and standard one-bit quantization for maximum ratio combining (MRC) and zero-forcing (ZF) receivers, achieving strong energy and spectral efficiency. However, one-bit systems require significantly more antennas, especially for ZF, to match the performance of full-resolution architectures^15^. Doubly one-bit quantized massive MIMO systems, despite their minimal RF complexity and power consumption, can achieve mean squared error (MSE) and symbol error rate (SER) performance close to that of full-resolution massive MIMO systems, with future extensions considering imperfect channel state information (CSI) and overall energy efficiency^16^. Only^17,18^ studied massive MIMO systems employing spatial ΣΔ modulation with one- or two-bit ADCs, using an elementwise Bussgang decomposition to derive linear minimum mean-squared error (LMMSE) channel estimators that account for the correlation among quantizer outputs, and further analyzed the achievable uplink rates for MRC, ZF, and LMMSE receivers. In^17^, channel estimation in massive MIMO systems employing spatial ΣΔ modulation with low-resolution (one- or two-bit) ADCs was investigated. Elementwise Bussgang decomposition was used to develop LMMSE channel estimators that account for the correlation among quantizer outputs. These estimators were shown to improve uplink performance when combined with MRC, ZF, or LMMSE receivers. Simulation results indicated that when users are confined to a specific angular sector or when antenna elements are spaced closer than half a wavelength, the spatial ΣΔ architecture significantly enhances channel estimation accuracy and spectral efficiency compared to conventional direct one- or two-bit quantization. Moreover, at low-to-moderate signal-to-noise ratios (SNRs), the performance of ΣΔ-based systems approaches that of systems with infinite-resolution ADCs. Theoretical expressions for channel estimation error closely match simulation results, and lower bounds on achievable spectral efficiency have been established. These findings highlight the potential of spatial ΣΔ arrays to improve energy efficiency, reduce hardware complexity, and lower fronthaul data rates in large-scale antenna systems.

### Contributions

In this work, we focus on uplink reception, adopting an approach that defines the quantization noise in a manner consistent with the spatial Sigma–Delta (ΣΔ) architecture^19^. We exploit the Bussgang decomposition, which provides an equivalent linear signal-plus-quantization-noise model, to derive a linear minimum mean-squared error (LMMSE) channel estimator for both one- and two-bit quantization. Using approaches like^20–22^, we derive a lower bound on the achievable uplink rate for a linear receiver based on the proposed LMMSE channel estimate. A more detailed analysis of the achievable rate bounds and estimation error is presented in^23^.

Simulation results for channel estimation and spectral efficiency using MRC and ZF receivers demonstrate that the spatial ΣΔ architecture significantly outperforms standard 1–2-bit quantization. Low-complexity receivers are crucial for the practical implementation of NOMA systems^3^. In particular, MRC receivers are attractive in massive MIMO systems due to their computational simplicity^24^. Combining MRC with group successive interference cancellation (GSIC) can therefore provide a robust and low-complexity receiver architecture for systems employing low-resolution ADCs.

To the best of the authors’ knowledge, existing studies mainly focus on transmit power minimization for a given quality-of-service (QoS), without a systematic comparison of the power-scaling behavior of linear, SIC, and GSIC receivers. Consequently, it remains unclear how key system parameters—such as the number of antennas, ADC resolution, Rician K-factor, and number of groups—affect system performance. Therefore, this paper investigates the performance of GSIC receivers by conducting a unified asymptotic analysis of linear, SIC, and GSIC receivers to characterize their power-scaling behavior. Both Rayleigh and Rician fading channels are considered under perfect and imperfect CSI. Furthermore, we compute the minimum transmit power required to satisfy the QoS constraints of each UE and derive the corresponding closed-form approximations for NOMA systems.

The main contributions are summarized as follows:

- Integration of Spatial Sigma–Delta ADCs: Unlike conventional low-resolution ADCs, the proposed spatial ΣΔ structure shapes quantization noise directionally, effectively reducing distortion while keeping hardware complexity low.
- Advanced LMMSE Channel Estimation under Quantization: A Bussgang-based LMMSE estimator is developed for quantized NOMA–MIMO systems, accurately modeling the nonlinear ADC behavior and enabling reliable channel estimation under both perfect and imperfect CSI in Rayleigh and Rician channels.
- Unified Asymptotic Spectral Efficiency Analysis: Closed-form asymptotic SINR and SE expressions are derived for MRC, ZF, SIC, and GSIC receivers. The analysis shows that ADC resolution and Rician K-factor are key limiting factors, and reveals that for very large antenna arrays, the SE of all receivers converges, while ZF-GSIC outperforms MRC-based receivers when the antenna count is moderate.
- Power-Scaling Laws and Energy Efficiency: The study establishes a power-scaling law, demonstrating that transmit power can decrease inversely with the number of BS antennas. It also shows that moderate-resolution ADCs, strong LoS paths, and ZF-based receivers significantly improve energy efficiency (EE).
- Practical QoS-Aware Power Allocation: Using Random Matrix Theory (RMT), an approximate power allocation scheme is proposed to meet target QoS requirements, showing that SIC-based receivers (MRC-SIC, MRC-GSIC) require substantially less transmit power than linear MRC.
- Comprehensive Performance Validation: Extensive numerical simulations confirm the accuracy of the asymptotic approximations and validate that 2-bit Sigma–Delta ADCs combined with low-complexity MRC-GSIC receivers can achieve high SE and EE, with the optimal trade-off reached when using a medium number of user groups.

### Keywords

The standard mathematical conventions are used to denote variables: matrices are represented by boldface uppercase letters (e.g., **A**) vectors by boldface lowercase letters (e.g., **a**) and scalars by plain lowercase letters (e.g., a). For specific matrix operations, the $\:N\:\times\:\:N$ identity matrix is denoted by $\:{\mathbf{I}}_{\mathbf{N}}$. Furthermore, column extraction is indicated by $\:{\left[\cdot\:\right]}_{:}{,}_{k}$, which denotes the k-th column of a matrix, while the entry located at the $\:{\left(m,k\right)}_{th}$ column of a matrix is denoted by $\:{\left[\cdot\:\right]}_{m}{,}_{n}$.

The remaining of the paper is organized as follows. The system model is introduced in Sect.  2. While achievable uplink rate analysis is presented in Sect.  3. Asymptotic Performance and Computational Complexity Analysis is studied in Sect.  4. MRC-GSIC Receiver Performance via Strategic Power Allocation are proposed and derived in Sect.  5. The asymptotic analysis is validated with numerical results in Sect.  6 and the conclusions are given in Sect.  7.

## System model

**Fig. 1:**
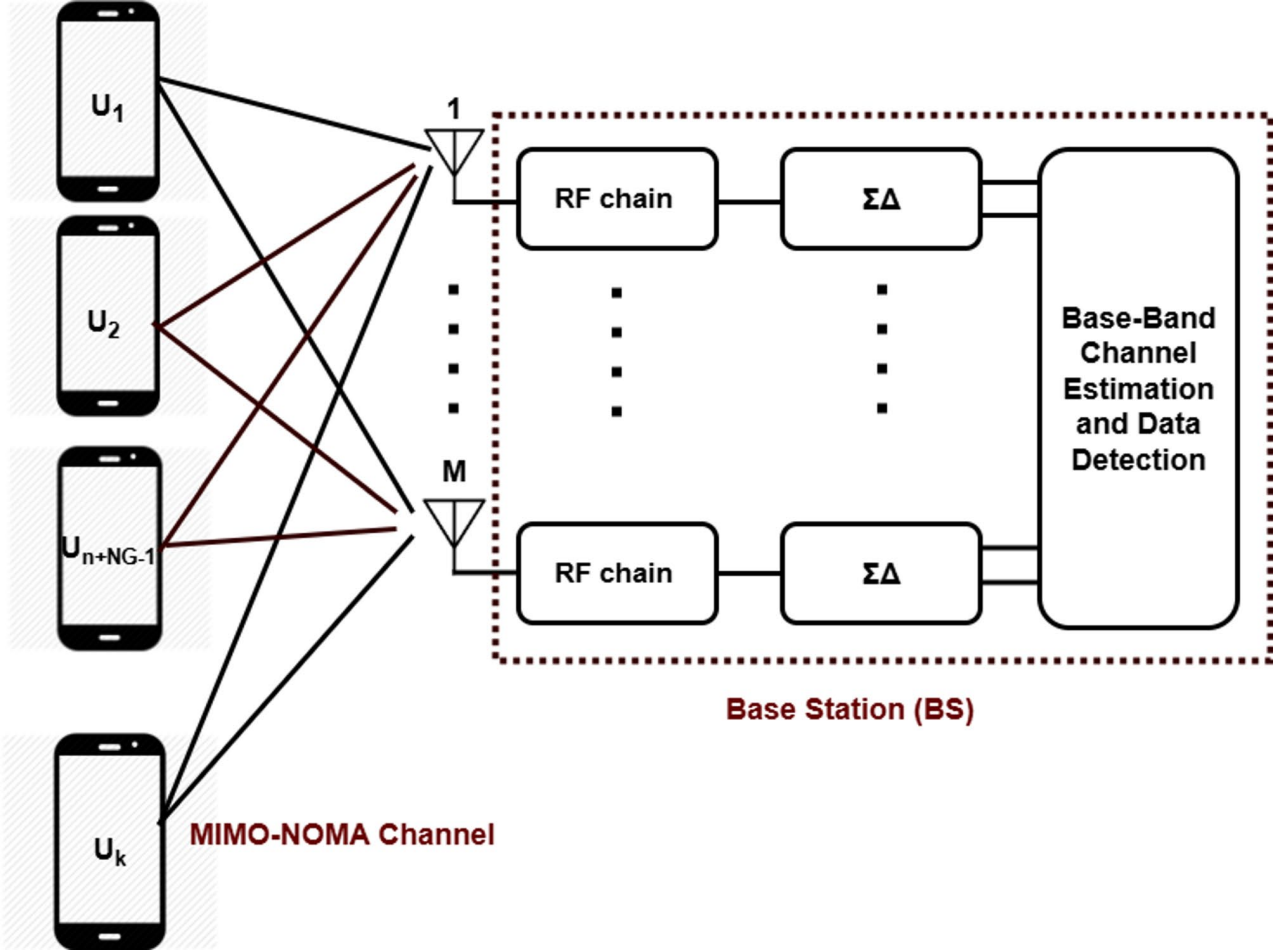
A massive MIMO system with Sigma-Delta $\:\left({\Sigma\:}{\Delta\:}\right)$ ADC and baseband channel estimation and data decision.

Consider the uplink of a single-cell MIMO system where the BS is equipped with $\:M$ antennas and serves $\:K$single-antenna user equipment (UEs) is presented in Fig. 1. The cell is assumed to be circular with radius $\:{d}_{max}\:$. In addition, $\:{H}_{m,k}\:\:$ with $\:\:\left(m,k\right)$-th entry of the channel matrix $\:H$, representing the channel gain between the $\:k$-th UE and the $\:m$-th BS antenna. Following^25,26^, the BS antennas are arranged in a uniform linear array (ULA) with half-wavelength spacing ( $\:d=\frac{\lambda\:}{2}$ ) where $\:d$ is the antenna spacing and $\:\lambda\:$ is the carrier wavelength. In line with^5^, this work focuses on power-domain massive MIMO–NOMA systems employing ΣΔ-ADCs and SIC or GSIC at the receiver. The discussion of the proposed system model begins with the channel model, as follows:

### Channel model

We define $\:\mathbf{G}\in\:\:{\mathbb{C}}^{M\times\:K}\:$as the channel gain between the $\:K$ UEs and *M* BS antennas, where its $\:\left(m,\:k\right)-$th entry denotes the channel response between the $\:{k}^{th}$ UE and the $\:{m}^{th}$ BS antenna. We have

1 $$\:\mathbf{G}\:=\stackrel{\sim}{\mathbf{G}}{\mathbf{D}}^{\frac{1}{2}}\:\:$$

where $\:\mathbf{D}=\mathrm{d}\mathrm{i}\mathrm{a}\mathrm{g}\:\left({{\upbeta\:}}_{1}\:,{{\upbeta\:}}_{2},\dots\:.,{{\upbeta\:}}_{k}\right)$ denotes as a diagonal matrix with its $\:{k}^{th}$ diagonal entry $\:\:{\boldsymbol{\varTheta\:}}_{kk}\:=\:{\mathcal{K}}_{k}\:$and $\:{\mathcal{K}}_{\boldsymbol{k}}\:$is the Rician $\:\mathcal{K}$-Factor for user $\:k$. In addition, $\:{\beta\:}_{\mathrm{K}}$ and $\:\stackrel{\sim}{\mathbf{G}}$ denote the large-scale fading for $\:k$ UE and small-scale fading between $\:k-$ UE and the $\:{m}^{th}$BS antenna, respectively. Consequently, $\:\stackrel{\sim}{\mathbf{G}}$ can be modeled as follows:

2 $$\:\mathbf{G}={{\mathbf{G}}^{\mathrm{L}\mathrm{o}\mathrm{S}}\:\left[\boldsymbol{\Theta\:}\:\right(\boldsymbol{\Theta\:}+\:{\mathbf{I}}_{\mathrm{k}})}^{-1}{]}^{1/2}+{\mathbf{G}}^{\mathrm{N}\mathrm{L}\mathrm{o}\mathrm{S}}\:{\left[\right(\boldsymbol{\Theta\:}\:+{\mathbf{I}}_{\mathrm{k}})}^{-1}{]}^{1/2}$$

where $\:{\mathbf{G}}^{\mathrm{L}\mathrm{o}\mathrm{S}}$ and $\:{\mathbf{G}}^{\mathrm{N}\mathrm{L}\mathrm{o}\mathrm{S}}\:$ denote the line-of-sight (LoS) and non-LoS (NLoS) channel components, respectively. Following^27,28^, the NLoS components are presented as:

3 $$\:{{[\mathbf{G}}^{\mathrm{N}\mathrm{L}\mathrm{o}\mathrm{S}}\:\:]}_{m,k}={e}^{-\:\mathrm{j}(m-1)\:\left)\:\frac{\:2{\uppi\:}\mathrm{d}\:}{{\uplambda\:}}\:\mathrm{s}\mathrm{i}\mathrm{n}\right({{\uptheta\:}}_{\mathrm{k}}\:)}\:\:\:$$

where $\:{{\uptheta\:}}_{\mathrm{k}}$ is the angle of arrival (AoA) for $\:{k}^{th}$ UE, and it is assumed to be uniformly distributed in $\:\left[-\frac{{\uppi\:}}{2},\frac{{\uppi\:}}{2}\right]$.

### Signal model

The received signal by BS is given as:

4 $$\:\mathbf{y}\:=\:\sqrt{{\mathrm{p}}_{\mathrm{s}}}\:\mathbf{G}\:\mathbf{s}+\mathbf{v}=\:\sqrt{{\mathrm{p}}_{\mathbf{s}}}\:\:\:{\sum\:}_{\mathrm{k}=1}^{\mathrm{K}}{\mathbf{g}}_{\mathrm{k}}\:{\mathrm{s}}_{\mathrm{k}}\:+\mathbf{v}$$

where $\:\mathbf{G}\:=\:\left[{\mathrm{g}}_{1},\:{\mathrm{g}}_{2},\:\dots\:,\:{\mathrm{g}}_{k}\right]\in\:\:{\mathbb{C}}^{M\times\:k}\:$is the channel matrix, $\:{\mathbf{S}\:=\:\left[{\mathrm{s}}_{1},\:{\mathrm{s}}_{2},\dots\:,\:{\mathrm{s}}_{k}\:\right]}^{\mathrm{T}}\:$ is the transmitted signal vector, $\:{\mathrm{P}}_{\mathrm{s}}$ is the transmit power for all UEs, and $\:\mathbf{v}\:\in\:\:{\mathbb{C}}^{M\times\:1}\:$is the additive white Gaussian noise (AWGN) with zero mean variance$\:\:{{\upsigma\:}}^{2}$. As shown in Fig. 1, the analog observations are converted into digital signals using $\:{\Sigma\:}{\Delta\:}$**-**ADCs. We assume that finite-resolution ΣΔ converters are equipped on the RF chains at the BS. To characterize the effect of quantization noise, we adopt the additive quantization noise model (AQNM), and the output can be expressed as

5 $$\:{\mathbf{y}}_{\mathrm{q}}\:=\:\mathbf{A}.\mathbf{y}\:+\:{\mathbf{v}}_{\mathrm{q}}\:=\:\mathbf{A}\sqrt{{\mathrm{p}}_{\mathrm{s}}\:}\:\:\mathbf{G}\:\mathbf{s}+\:\mathbf{A}\:\mathbf{v}\:+\:{\mathbf{v}}_{\mathbf{q}}$$

$\:\mathbf{A}\:$is a diagonal matrix **A** = dig {$\:\:{a}_{1}$,$\:\:{a}_{2},\dots\:,{a}_{m}$} and where$\:{\:a}_{m}$ is scale factor and the additive Gaussian quantization noise $\:{\mathbf{v}}_{\mathbf{q}}$ is uncorrelated with **y**. Moreover, the covariance matrix of the quantization noise $\:{\mathbf{v}}_{\mathbf{q}}$ is calculated by:

6 $$\:{{\mathbf{R}}_{\mathrm{v}}}_{\mathrm{q}}=\mathbb{E}\:\left\{{\mathbf{v}}_{\mathrm{q}}{{\mathbf{v}}_{\mathrm{q}}}^{\mathrm{H}}|\mathrm{G}\right\}=\mathbf{A}\left(1-\mathbf{A}\right)\mathrm{d}\mathrm{i}\mathrm{a}\mathrm{g}\:\left(\mathbf{G}{\mathbf{R}\mathrm{x}\mathbf{G}}^{\mathrm{H}}+\:{{\upsigma\:}}^{2}{\mathbf{I}}_{M}\right)$$

where $\:\mathbf{R}\mathrm{x}$ *is a* $\:K\:\times\:\:K\:$diagonal matrix whose $\:{k}^{th}$ diagonal entry equals $\:{\mathrm{P}}_{\mathrm{s}}$. The vectors **s**,** v** and $\:{\mathbf{v}}_{\mathrm{q}}$ are assumed to be mutually independent. The entries of $\:{\mathbf{v}}_{\mathbf{q}}$ have zero meaning, and their variances correspond to the respective diagonal elements of $\:{{\:\mathbf{R}}_{\mathrm{v}}}_{\mathrm{q}}$. In practice, the CSI is often obtained during a training phase. In this paper, we consider the LMMSE channel estimation similar in^24,27^. Where each UE transmits a pilot signal of length L, the transmission power of UE$\:\:k$ $\:{p}_{k}^{ce}$, the large-scale fading coefficient $\:{\beta\:}_{k}\:$and the deterministic component $\:{\mathbf{G}}^{\mathrm{L}\mathrm{o}\mathrm{S}}$ are known at the BS. The channel estimation error for UE $\:k$

7 $$\:{\:\:\:\mathbf{e}}_{\mathrm{k}}={\mathbf{G}}_{\mathrm{k}}-{\widehat{\mathbf{G}}}_{\mathrm{k}\:}=\frac{1}{\sqrt{{K}_{k}+1}}\:\left({\mathbf{G}}_{\mathrm{N}\mathrm{L}\mathrm{o}\mathrm{s}.\:\mathrm{k}\:}-{\widehat{\mathbf{G}}}_{\mathrm{N}\mathrm{L}\mathrm{o}\mathrm{s},\:\:\mathrm{k}\:\:}\right)$$

It can be shown that the variance of the entries in $\:{\mathbf{e}}_{\mathbf{k}}$can be computed as:

8 $$\:{{{\:{\upsigma\:}}^{2}}_{\mathrm{e}}}_{k}=\:\frac{{\:{\upbeta\:}}_{\mathrm{k}}\:-{\:{\upbeta\:}}_{\mathrm{k}}{{\upeta\:}}_{\mathrm{k}\:}}{{\mathrm{K}}_{\mathrm{k}}+1}$$

9 $$\:{{\upeta\:}}_{\mathrm{k}\:}=\frac{{{p}_{k}^{ce}\mathrm{L}{\upbeta\:}}_{\mathrm{k}}}{{\mathrm{P}}_{\mathrm{k}}^{\mathrm{c}\mathrm{e}}\mathrm{L}{{\upbeta\:}}_{\mathrm{k}}+{(K}_{k}+1)\left[\frac{{{\upsigma\:}}^{2}}{\mathrm{a}}+(\frac{1}{\mathrm{a}}-1){\sum\:}_{\mathrm{i}=k}^{K}{p}_{i}^{ce}{{\upbeta\:}}_{\mathrm{i}}\right]\:}$$

In NOMA systems, all UEs share the same frequency, making the choice of multi-user detectors critical for system performance. As discussed, MRC offers low complexity but is susceptible to inter-user interference (IUI). This interference can be mitigated using a ZF receiver, while ZF-SIC receivers further improve performance by alleviating noise enhancement. In this section, we analyze the SE of systems employing MRC-GSIC, which encompasses linear MRC and MRC-SIC as special cases. For the transmission rate with a ZF-GSIC receiver, we follow the approach in^27^. Specifically, the K users are divided into $\:{N}_{G}$ groups, each containing $G=\left\lfloor {\frac{K}{{{N_G}}}} \right\rfloor$UEs, where $\:1\le\:{N}_{G}\le\:K$. The $\:{g}_{n}$-th group consists of UEs with indices $\:k=G\left({g}_{n}-1\right)+1$to $\:k=G\cdot\:{N}_{G}$. For any given UE $\:k$, its group index is determined as ${g_n}=\left\lfloor {\frac{n}{G}} \right\rfloor$. Detection is performed successively group by group, while all UEs within the same group are detected in parallel. Assuming ideal interference cancellation from previously processed groups, the received signal for UE $\:k$can then be estimated accordingly.

10 $$\:{\widehat{\mathrm{s}}}_{\mathrm{k}}=\:{\mathbf{g}}_{\mathrm{k}}^{H}\:\left({\mathbf{y}}_{\mathrm{q}}\:-\:{\mathrm{a}}_{\mathrm{m}}\sum\:_{\mathrm{i}=1}^{\left({g}_{n}-1\right)\mathrm{G}\:}{\mathbf{g}}_{\mathrm{i}}\:{\mathrm{s}}_{\mathrm{i}}\right)=\:{\mathbf{g}}_{\mathrm{k}}^{H}\:{\mathbf{y}}_{\mathrm{q}}\:\:-\:\sum\:_{\mathrm{i}=1}^{\left({g}_{n}-1\right)\mathrm{G}\:}\:{\boldsymbol{\updelta\:}}_{\mathrm{k}\mathrm{i}}{\:\mathrm{s}}_{\mathrm{i}}$$

where $\:{\mathbf{g}}_{\mathrm{k}}$ is the receiver filter for $\:{k}^{th}$ UE and $\:{{\updelta\:}}_{ki}\approx\:{\mathrm{a}}_{\mathrm{m}}\:{\mathbf{g}}_{\mathrm{k}}^{H}\:\:{\mathbf{g}}_{\mathrm{i}}$.

## Achievable uplink rate analysis for $\:\boldsymbol{\Sigma\:}\boldsymbol{\Delta\:}$ array

**Fig. 2:**
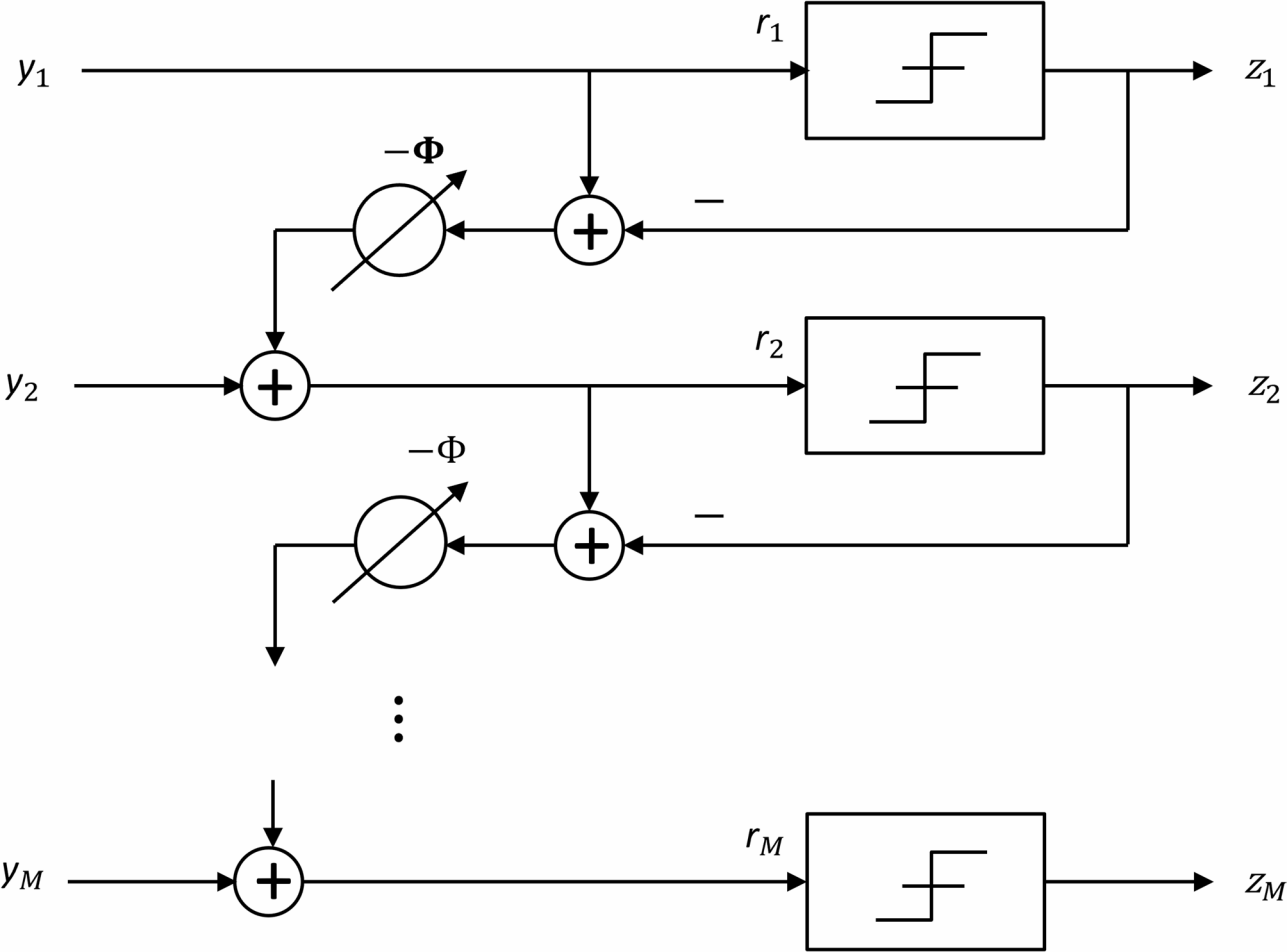
A first**-**order one-bit$\:\:{\Sigma\:}{\Delta\:}$ array steered to direction $\:{\Phi\:}$.

To gain a clear understanding of the spatial ΣΔ array and facilitate the derivation of its equivalent linear model, it is helpful to first examine its architectural design. As depicted in Fig. 2, the array consists of $\:M$ antenna elements arranged with a feedback mechanism to achieve spatial noise shaping. Specifically, the quantization error from one antenna is phase-shifted by an angle $\:{\Psi\:}$ and fed into the input of the next antenna. This feedback loop effectively redistributes the quantization noise to spatial frequencies away from the AoA corresponding to $\:{\Phi\:}$, resulting in a high signal-to-quantization-noise ratio (SQNR) for users within that angular sector. The extent of this high-SQNR region is influenced by the spatial oversampling factor, which is practically limited by mutual coupling between antenna elements. Figure 2 provides a schematic overview of this architecture, illustrating the input signals $\:{y}_{m}$, the quantizer inputs $\:{r}_{m}$, the feedback paths implementing the phase shifts, and the digitized outputs $\:{z}_{m}$. This visualization offers an intuitive understanding of the ΣΔ array operation prior to introducing the mathematical model. The system is represented using $\:MN\times\:1$ vectors spanning $\:M\:$antennas and $\:N$ training samples. The main vectors include the received signal $\:\mathbf{y}$, the quantizer input $\:r$, and the array output $\:\mathbf{z}$. Each element of these vectors, $\:\left\{{y}_{m},{r}_{m},{z}_{m}\right\}$, corresponds to antenna index $\:{m}^{{\prime\:}}$ = “*mod*”_*M*_ (*m*) for the training sample $\:\left[\frac{m}{M}\right]$.

The array output is produced by applying an antenna-dependent complex quantization function $\:Q\left(\cdot\:\right)$ to the input $\:r$. The element-wise output $\:{z}_{m}\:$is defined as:

11 $$\:{z}_{m}={a}_{{m}^{{\prime\:}}}{Q}_{{m}^{{\prime\:}}}\left(\mathrm{Re}\left({r}_{m}\right)\right)+j{a}_{{m}^{{\prime\:}}}{Q}_{{m}^{{\prime\:}}}\left(\mathrm{Im}\left({r}_{m}\right)\right)$$

where $\:{Q}_{{m}^{{\prime\:}}}$denotes the quantization operation for antenna $\:{m}^{{\prime\:}}$, and $\:{a}_{{m}^{{\prime\:}}}$represents the quantizer output level. Unlike conventional one-bit quantization where the output levels are identical across antennas, $\:{a}_{{m}^{{\prime\:}}}$ can vary for each antenna in this design. In vector form, the ΣΔ array output can be expressed as:

12 $$\:\boldsymbol{z}=Q\left(\boldsymbol{r}\right)={\left[{Q}_{1}\left({r}_{1}\right),\dots\:,{Q}_{M}\left({r}_{M}\right),{Q}_{1}\left({r}_{M+1}\right),\dots\:,{Q}_{M}\left({r}_{MN}\right)\right]}^{T}$$

The quantizer input $\:\boldsymbol{r}$ is generated through a linear combination of the received signal $\:\boldsymbol{y}$ and the array output $\:\boldsymbol{z}$, implementing a feed-forward/feedback network characterized by matrices $\:\mathbf{U}$ and $\:\mathbf{V}$:

13 $$\:\mathbf{r}=\mathbf{U}\mathbf{y}-\mathbf{V}\:$$

Here, the matrices $\:\mathbf{U}\:$and $\:\mathbf{V}$ are constructed using the $\:N\times\:N\:$identity matrix and define the weights for the feed-forward/feedback paths, respectively, thereby shaping the quantization process across the array. The Kronecker product $\otimes$ is given by:

14 $${\mathbf{V}}={{\mathbf{I}}_N} \otimes \underbrace {{\left[ {\begin{array}{*{20}{c}} 0&0& \ldots &0&0 \\ {{e^{ - j{{\boldsymbol{\Phi}}}}}}&0& \ldots &0&0 \\ {{e^{ - j2{{\boldsymbol{\Phi}}}}}}&{{e^{ - j{{\boldsymbol{\Phi}}}}}}& \ldots &0&0 \\ {}&{}& \vdots &{}&{} \\ {{e^{ - j\left( {M - 1} \right){{\boldsymbol{\Phi}}}}}}&{{e^{ - j\left( {M - 2} \right){{\boldsymbol{\Phi}}}}}}& \ldots &{{e^{ - j{{\boldsymbol{\Phi}}}}}}&0 \end{array}} \right]}}_{{{{\mathbf{V}}_{\mathbf{d}}}}}~$$

15 $${\mathbf{U}}={{\mathbf{I}}_{\mathrm{N}}} \otimes \underbrace {{{{\mathbf{I}}_{\mathrm{M}}}+{{\mathbf{V}}_{\mathrm{d}}}}}_{{{{\mathrm{U}}_{\mathrm{d}}}}}$$

In the next section, we will present an equivalent linear model to describe the output of the $\:{\Sigma\:}{\Delta\:}$ array in terms of **r**. This linear model, combined with the structure of the matrices **U** and **V**, is used to formulate a recursion for the computation of the output power, a quantity that is essential for LMMSE channel estimation.

### Equivalent linear model for the $\:\varSigma\:\varDelta\:$ array

The operation of the $\:{\Sigma\:}\varDelta\:$ array using an equivalent linear model is defined by:

16 $$\:\mathbf{Z}=\boldsymbol{\Gamma\:}\mathbf{r}+\mathbf{q}$$

for a given matrix **Γ**, **q** is the equivalent quantization noise. There are an infinite number of such models, one for choice of **Γ**, and each will result in a particular definition for every quantization noise with differing statistical properties. One possible choice for the matrix **Γ** is obtained by making **r** uncorrelated with **q**, i.e. $\:\mathbb{E}$ [**r** $\:{\mathbf{q}}^{\mathrm{H}}$] .This leads to $\:\boldsymbol{\Gamma\:}\:=\:\text{}{\mathrm{C}}_{\mathrm{z}\mathrm{r}}\:{{\mathrm{C}}_{\mathrm{r}}}^{-1}$​,where $\:{\mathrm{C}}_{\mathrm{z}\mathrm{r}}\mathbb{}=\mathbb{\:}\mathbb{E}\mathbb{\:}\left[\mathbf{z}{\:\mathbf{r}}^{\mathrm{H}}\right]$is the cross-covariance matrix between **z** and **r**, and $\:{\mathbf{C}}_{\mathbf{r}}$ is the covariance matrix of **r**. Assuming the elements of$\:\:\:r\:$are jointly Gaussian, the Bussgang theorem^28^ can be applied. This approach was used to derive channel estimates in^20^ for conventional one-bit quantization, and in our initial work on the $\:{\Sigma\:}\varDelta\:\:$array^29^. However, the resulting definition for the quantization noise does not have a physical interpretation in the context of Fig. 2. Instead, we define $\:\boldsymbol{\Gamma\:}$ following the approach of^19^, applying the Bussgang decomposition elementwise such that $\:\mathbb{E}\left[{r}_{m}{q}_{m}^{*}\right]$. With this definition, $\:\boldsymbol{\Gamma\:}$ becomes a diagonal matrix whose $\:{m}^{th}$ diagonal element, $\:{{\upgamma\:}}_{\mathrm{m}}^{{\prime\:}}\:$, is given by:

17 $$\:{{\upgamma\:}}_{\mathrm{m}}\:=\:\:\frac{\mathbb{E}\left[{\mathrm{r}}_{\mathrm{m}}\:{\mathbf{z}}_{m}^{*}\right]}{\mathbb{E}\left[{\left|{\mathrm{r}}_{\mathrm{m}}\right|}^{2}\right|]}$$

By substituting $\:\boldsymbol{r}=\boldsymbol{U}\boldsymbol{y}-\boldsymbol{V}\boldsymbol{z}$ into Eq. (16), we obtain

18 $$\:\begin{array}{cccc}&\:\boldsymbol{z}=(\boldsymbol{I}+\boldsymbol{\Gamma\:}\boldsymbol{V}{)}^{-1}\boldsymbol{\Gamma\:}\boldsymbol{U}\boldsymbol{y}+(\boldsymbol{I}+\boldsymbol{\Gamma\:}\boldsymbol{V}{)}^{-1}\boldsymbol{q}&\:&\:\end{array}$$

The specific value of $\:{\gamma\:}_{{m}^{{\prime\:}}}$depends on the chosen output level $\:{a}_{{m}^{{\prime\:}}}$. To simplify the analysis, we select $\:{a}_{{m}^{{\prime\:}}}\:$such that $\:{\gamma\:}_{{m}^{{\prime\:}}}=1$, or equivalently $\:\boldsymbol{\Gamma\:}={\mathbf{I}}_{\boldsymbol{M}\boldsymbol{N}}$. Under this condition, Eq. (18) simplifies to

19 $$\:\begin{array}{cccc}&\:\boldsymbol{z}=\boldsymbol{y}+{\boldsymbol{U}}^{-1}\boldsymbol{q}&\:&\:\end{array}$$

This configuration is particularly convenient because it represents the exact spatial counterpart of the conventional temporal $\:\varSigma\:\varDelta\:$ modulator. In this model, the signal $\:x$effectively passes through a spatial all-pass filter, while the quantization noise $\:q$is shaped spatially away from the AoA associated with $\:{\Phi\:}$. Although this choice of $\:{a}_{{m}^{{\prime\:}}}$yields strong performance in simulations, we do not provide formal proof of its optimality. Deriving such proof appears analytically challenging and is thus left for future work. Nevertheless, deviating from $\:{\gamma\:}_{{m}^{{\prime\:}}}=1$would be difficult to justify, as it would imply that the input signal $\:x$is spatially distorted by the ADC in an unclear manner. As discussed in the subsequent sections for one- and two-bit quantization, the optimal value of $\:{a}_{{m}^{{\prime\:}}}$generally depends on the statistical properties of $\:{r}_{m}$, which must remain time-invariant for consistent quantizer gains. In practice, this can be ensured by employing automatic gain control (AGC) at the ADC input.

**A. 1 bit Sigma-Delta ADC**.

The output of $\:1-$bit $\:{\Sigma\:}\varDelta\:-\:$ADC can be expressed as:

20 $$\:{\mathbf{z}}_{\mathrm{m}}\:=\:{a}_{\mathrm{m}{\prime\:}}\left(\mathrm{S}\mathrm{i}\mathrm{g}\mathrm{n}\right(\mathrm{R}\mathrm{e}\left({\mathrm{r}}_{\mathrm{m}}\right))\:+\:j\:\mathrm{S}\mathrm{i}\mathrm{g}\mathrm{n}(\mathrm{I}\mathrm{m}\left({\mathrm{r}}_{\mathrm{m}}\right)\left)\right)$$

Then Eq. (17) can be simplified to:

21 $$\:{{\upgamma\:}}_{\mathrm{m}}={a}_{\mathrm{m}{\prime\:}}\:\frac{\mathbb{E}\left[\mathrm{R}\mathrm{e}\right({\mathrm{r}}_{\mathrm{m}}\left)\right|+\left|\mathrm{I}\mathrm{m}\right({\mathrm{r}}_{\mathrm{m}}\left)\mathrm{j}\right]}{\mathrm{E}\left[{\left|{\mathrm{r}}_{\mathrm{m}}\right|}^{2}\right|]}={a}_{\mathrm{m}{\prime\:}}\:\frac{\mathbb{E}\left[\mathrm{R}\mathrm{e}\right({\mathrm{r}}_{\mathrm{m}}\left)\right|]}{\mathbb{E}\left[{\left|{\mathrm{r}}_{\mathrm{m}}\right|}^{2}\right|]}$$

Since the power of the pilot symbols is time$\:-$invariant, the statistics of $\:{\mathrm{r}}_{\mathrm{m}}$ are identical to those of$\:\:{\mathrm{r}}_{\mathrm{m}}$ because of circularly symmetric $\:{\mathrm{r}}_{\mathrm{m}}$. Consequently, we can set $\:{{\upgamma\:}}_{\mathrm{m}}=1$, which leads to:

22 $$\:{a}_{\mathrm{m}{\prime\:}}=\:\frac{\mathbb{E}\left[{\mathrm{R}\mathrm{e}\left({\mathrm{r}}_{\mathrm{m}}\right)|}^{2}\right]}{\mathbb{E}\left[{\left|{\mathrm{r}}_{\mathrm{m}}\right|}^{2}\right|]}$$

If $\:{\mathrm{r}}_{\mathrm{m}}$ were Gaussian, Eq. (22) could be simplified to:

23 $$\:{a}_{\mathrm{m}{\prime\:}}=\:\frac{\sqrt{{\uppi\:}\mathrm{E}\left[{\left|{\mathrm{r}}_{\mathrm{m}}\right|}^{2}\right|]}}{2}$$

The value for $\:{a}_{\mathrm{m}{\prime\:}}$ provides sufficiently accurate estimates of the spectral efficiency for the case of CSI is already known. However, the deviation of $\:{\mathrm{r}}_{\mathrm{m}}$ from Gaussianity, while not large, is sufficient to render Eq. (22) unsuitable for channel estimation. The derivation of Eq. (22) for Gaussian random variables relies on the fact that:

24 $$\:\frac{\sqrt{\mathbb{E}\left[\right|\mathrm{R}\mathrm{e}\left({\mathrm{r}}_{\mathrm{m}}\right)|{]}^{2}}}{\mathbb{E}\left[\right|\mathrm{R}\mathrm{e}\left({\mathrm{r}}_{\mathrm{m}}\right)\left|\right]}\:\:=\:\sqrt{\frac{{\uppi\:}}{2}}$$

However, due to the non-linear feedback of the $\:{\Sigma\:}\varDelta\:$ array, the tails of the distribution of $\:{\mathrm{r}}_{\mathrm{m}}$ are slightly heavier than a Gaussian, so the ratio on the left hand side of (24) is slightly greater than $\:\sqrt{{\uppi\:}/2}$. Whether Eq. (19) or (20) is used to calculate $\:{a}_{\mathrm{m}{\prime\:}}$, in a practical implementation some empirical measurement of the mean power and absolute value of $\:{\mathrm{r}}_{\mathrm{m}}$ in the $\:{\Sigma\:}\varDelta\:$ architecture would be necessary and could be facilitated using an AGC. Rather than implementing the computation of $\:{a}_{\mathrm{m}{\prime\:}}\:$ according to Eq. (23), in our simulations of the one-bit case presented later, we simply calculate $\:{a}_{\mathrm{m}{\prime\:}}$ as:

25 $$\:{a}_{\mathrm{m}{\prime\:}}={\upbeta\:}\frac{\sqrt{{\uppi\:}\mathrm{E}\left[{\left|{\mathrm{r}}_{\mathrm{m}}\right|}^{2}\right|]}}{2}$$

with a value of $\:{\upbeta\:}\:>\:1$. But there is a very small range of values near that are appropriate for one. To see this, let $\:{{{\upsigma\:}}^{2}}_{\mathrm{y}\mathrm{m}}\:=\mathrm{E}[{\left|{\mathrm{r}}_{\mathrm{m}}\right|}^{2}$ Using similar definitions $\:{{{\upsigma\:}}^{2}}_{\mathrm{y}\mathrm{m}}\:\mathrm{a}\mathrm{n}\mathrm{d}\:{{{\upsigma\:}}^{2}}_{\mathrm{q}\mathrm{m}}$ for fact that$\:\:{\:\mathrm{r}}_{\mathrm{m}}$ and $\:{\:\mathrm{q}}_{\mathrm{m}}$ are uncorrelated, we obtain the following relationship between the powers of the input and output of the array:

26 $$\:{{{\upsigma\:}}^{2}}_{\mathrm{y}\mathrm{m}}\:=\:\frac{{\uppi\:}}{2}\:{\upbeta\:}\:{{{\upsigma\:}}^{2}}_{{\mathrm{r}}_{\mathrm{m}}}\:,\:\:{{{\upsigma\:}}^{2}}_{{\mathrm{q}}_{\mathrm{m}}}=\:\:{{{\upsigma\:}}^{2}}_{\mathrm{y}\mathrm{m}}\:-\:{{{\upsigma\:}}^{2}}_{{\mathrm{r}}_{\mathrm{m}}}$$

From Eq. (26), we can see that in order to prevent the quantization noise power from becoming greater than the input power $\:{{{\upsigma\:}}^{2}}_{{\mathrm{r}}_{\mathrm{m}}}$, we must ensure that $\:(\frac{{\uppi\:}}{2}\:{{\upbeta\:}}^{2}\:-\:1\:\:)\:<\:1$, and hence that $\:1\:\le\:\:{\upbeta\:}\:<\:2/\sqrt{{\uppi\:}}\:\approx\:1.1284$. Otherwise, the input power to each ADC grows monotonically with the antenna index.

**B.2 bit Sigma–Delta ADC**.

In this section, we extend the above analysis to the case where the quantizers in the Σ∆ array employ $\:2-$bits resolution with four quantization levels. Unlike the $\:1-$ bit case, the Gaussian approximation for $\:{\mathrm{r}}_{\mathrm{m}}$ is quite accurate with $\:2-$bits. We use the well-known Lloyd-max condition to determine the optimum quantization levels that minimize the distortion^29,30^. Hence, the quantization levels and the associated intervals will be denoted for minimization of the distortion for unit variance Gaussian inputs by $\:{\mathrm{v}}_{\mathrm{i}\:}\:$and ($\:{\mathrm{v}}_{\mathrm{i}}^{\mathrm{l}\mathrm{o}},\:{\mathrm{v}}_{\mathrm{i}}^{\mathrm{h}\mathrm{i}})\:,\:\mathrm{i}=\:1,\dots\:.,4,$, respectively.

27 $$\:{\:\:\mathrm{Q}}_{\mathrm{m}{\prime\:}\:\left({\mathrm{r}}_{\mathrm{m}}^{\mathrm{R}\mathrm{e}}\right)\:\:={\mathrm{v}}_{\mathrm{i}\:}\:}\:\mathrm{i}\mathrm{f}\:{\mathrm{r}}_{\mathrm{m}}^{\mathrm{R}\mathrm{e}}\:\in\:\:\:(\frac{{{\upsigma\:}}_{\mathrm{r}\:\mathrm{m}}}{\sqrt{2}}\:{\mathrm{v}}_{\mathrm{i}}^{\mathrm{l}\mathrm{o}},\:\frac{{{\upsigma\:}}_{\mathrm{r}\:\mathrm{m}}}{\sqrt{2}}\:{\mathrm{v}}_{\mathrm{i}}^{\mathrm{h}\mathrm{i}}\:)$$

where $\:{\mathrm{r}}_{\mathrm{m}}^{\mathrm{R}\mathrm{e}}\:$= Re($\:{\mathrm{r}}_{\mathrm{m}}\:)$ is the above quantization levels satisfy $\:{\mathrm{v}}_{\mathrm{i}}^{\mathrm{h}\mathrm{i}}\:={\mathrm{v}}_{\mathrm{i}+1}^{\mathrm{l}\mathrm{o}}$,$\:\:{\mathrm{v}}_{\mathrm{i}}^{\mathrm{l}\mathrm{o}}=-{\infty\:}\:\mathrm{a}\mathrm{n}\mathrm{d}\:{\mathrm{v}}_{4}^{\mathrm{h}\mathrm{i}}={\infty\:}$, and the quantization bins have been adjusted to span the range of the input levels by modeling $\:{\mathrm{r}}_{\mathrm{m}}$ as a circularly symmetric Gaussian random variable with variance $\:{{{\upsigma\:}}^{2}}_{{\mathrm{r}}_{\mathrm{m}}}$. Note that, while the convention is to also scale the output quantization level according to standard deviation of the input, we perform this scaling with the factor $\:{a}_{\mathrm{m}{\prime\:}}$. Assuming a linear model and using an elementwise Bussgang decomposition, Eq. (21) can be written as:

28 $$\:{{\upgamma\:}}_{\mathrm{m}}={a}_{\mathrm{m}{\prime\:}}\frac{\mathbb{E}\left[{\mathrm{r}}_{\mathrm{m}}^{\mathrm{R}\mathrm{e}}\:{z}_{\mathrm{m}}^{\mathrm{R}\mathrm{e}}\right]}{\mathbb{E}[{\left|{\mathrm{r}}_{\mathrm{m}}^{\mathrm{R}\mathrm{e}}\right]}^{2}}$$

Due to the circular symmetry of the data, where $\:{\mathrm{z}}_{\mathrm{m}}^{\mathrm{R}\mathrm{e}}$ $\:\mathrm{R}\mathrm{e}\left({\mathrm{r}}_{\mathrm{m}}\right).$The numerator of Eq. (28) can be obtained from Bussgang’s theorem^28^:

29 $$\:\mathrm{E}\left[{\mathrm{r}}_{\mathrm{m}}^{\mathrm{R}\mathrm{e}}\:{\mathrm{z}}_{\mathrm{m}}^{\mathrm{R}\mathrm{e}}\right]=\:{a}_{{\mathrm{m}}^{{\prime\:}}}\:\mathrm{E}\left[{\mathrm{r}}_{\mathrm{m}}^{\mathrm{R}\mathrm{e}}{\:\:\mathrm{Q}}_{{\mathrm{m}}^{{\prime\:}}\:}\:\left({\mathrm{z}}_{\mathrm{m}}^{\mathrm{R}\mathrm{e}}\right)\right]=\:{a}_{\mathrm{m}{\prime\:}}\underset{-{\infty\:}}{\overset{{\infty\:}}{\int\:}}\frac{1}{\sqrt{2{\uppi\:}}}\frac{{\partial\:\:\mathrm{Q}}_{\mathrm{m}{\prime\:}\:\:}\:\left({\mathrm{r}}_{\mathrm{m}}^{\mathrm{R}\mathrm{e}}\right)}{\partial\:{\mathrm{r}}_{\mathrm{m}}^{\mathrm{R}\mathrm{e}}}\mathrm{e}\mathrm{x}\mathrm{p}\:(-\frac{\left({\mathrm{r}}_{\mathrm{m}}^{\mathrm{R}\mathrm{e}}\right)}{{{{\upsigma\:}}^{2}}_{{\mathrm{r}}_{\mathrm{m}}}})\:\mathrm{d}{\mathrm{r}}_{\mathrm{m}}^{\mathrm{R}\mathrm{e}}=\:{a}_{\mathrm{m}{\prime\:}}\underset{-{\infty\:}}{\overset{{\infty\:}}{\int\:}}\frac{1}{\sqrt{2{\uppi\:}}}\frac{{\partial\:\:\mathrm{Q}}_{\mathrm{m}{\prime\:}\:\:}\:\left({\mathrm{r}}_{\mathrm{m}}^{\mathrm{R}\mathrm{e}}\right)}{\partial\:{\mathrm{r}}_{\mathrm{m}}^{\mathrm{R}\mathrm{e}}}\mathrm{e}\mathrm{x}\mathrm{p}\:(-\frac{\left({\mathrm{r}}_{\mathrm{m}}^{\mathrm{R}\mathrm{e}}\right)}{{{{\upsigma\:}}^{2}}_{{\mathrm{r}}_{\mathrm{m}}}})\:\mathrm{d}{\mathrm{r}}_{\mathrm{m}}^{\mathrm{R}\mathrm{e}}$$

The derivative $\:\frac{{\:\partial\:\:\mathrm{Q}}_{\mathrm{m}{\prime\:}\:\:}\:\left({\mathrm{r}}_{\mathrm{m}}^{\mathrm{R}\mathrm{e}}\right)}{\partial\:{\mathrm{r}}_{\mathrm{m}}^{\mathrm{R}\mathrm{e}}}$can be computed as:

30 $$\:\frac{{\:{\updelta\:}\:\mathrm{Q}}_{\mathrm{m}{\prime\:}\:\:}\:\left({\mathrm{r}}_{\mathrm{m}}^{\mathrm{R}\mathrm{e}}\right)}{{\updelta\:}{\mathrm{r}}_{\mathrm{m}}^{\mathrm{R}\mathrm{e}}}\:=\sum\:_{\mathrm{i}=2}^{4}\left({\mathrm{v}}_{\mathrm{i}\:}-{\mathrm{v}}_{\mathrm{i}-1\:}\right)\partial\:({\mathrm{r}}_{\mathrm{m}}^{\mathrm{R}\mathrm{e}}-\frac{{{\upsigma\:}}_{{\mathrm{r}}_{\mathrm{m}}}}{\sqrt{2}}\:{\mathrm{v}}_{\mathrm{i}}^{\mathrm{l}\mathrm{o}})$$

Using the Dirac delta function $\:\partial\:(.)$ to represent the derivative at the quantize steps. Substituting the above Eq. in (29) and evaluating the integral, we get:

Then, the value of $\:{a}_{\mathrm{m}{\prime\:}}$ those yields $\:{{\upgamma\:}}_{\mathrm{m}}=1$ is given by:

32 $$\:{a}_{\mathrm{m}{\prime\:}}=\frac{{{\upsigma\:}}_{{\mathrm{r}}_{\mathrm{m}}\:\sqrt{{\uppi\:}/2}}}{\sum\:_{\mathrm{i}=2}^{4}\frac{({\mathrm{v}}_{\mathrm{i}\:}-{\mathrm{v}}_{\mathrm{i}-1\:})}{\sqrt{2{\uppi\:}}}\:\mathrm{e}\mathrm{x}\mathrm{p}(-\frac{{\mathrm{v}}_{\mathrm{i}}^{\mathrm{l}\mathrm{o}}}{2}\:)}$$

According to the previous results, it is noticed that, $\:{a}_{{m}^{{\prime\:}}}\:$does not depend on the index $\:m$′ since the quantization levels and intervals for the m’$\:-$th ADC have been scaled by the standard deviation of the input in Eq. (30), e.g., by means of an AGC at the input. Thus, we will drop the dependence of $\:\mathrm{a}$ one $\:m\:$and choose the single value $\:a$ necessary to achieve $\:{{\upgamma\:}}_{\mathrm{m}}=1$. Note also that the result in Eq. (32) relies on the assumption that $\:{\mathrm{r}}_{\mathrm{m}}\:.$ is Gaussian. In reality, due to the non-linear feedback structure of the $\:{\Sigma\:}{\Delta\:}\:$array, the tails of the distribution of $\:{\mathrm{r}}_{\mathrm{m}}$ are slightly heavier than a Gaussian. In the same line, the ratio on the left-hand side of Eq. (32) is slightly greater than the right-hand side. For this reason, we will adopt a slightly larger value for $\:{a}_{\mathrm{m}{\prime\:}}$:

33 $$\:{a}_{\mathrm{m}{\prime\:}}=\left\{\begin{array}{c}\beta\:\sqrt{{\uppi\:}}/2\:\:\:\:\:\:\:\:\:\:\:\:\:\:\:\:\:\:\:\:\:\:\:\:\:\:\:\:\:\:\:\:\:\:\:\:\:\:\:\:\:\:\:\:\:\:for\:1-bit\\\:\frac{{{\upsigma\:}}_{{\mathrm{r}}_{\mathrm{m}}\:\sqrt{{\uppi\:}/2}}}{\sum\:_{\mathrm{i}=2}^{4}\frac{({\mathrm{v}}_{\mathrm{i}\:}-{\mathrm{v}}_{\mathrm{i}-1\:})}{\sqrt{2{\uppi\:}}}\:\mathrm{e}\mathrm{x}\mathrm{p}(-\frac{{\mathrm{v}}_{\mathrm{i}}^{\mathrm{l}\mathrm{o}}}{2}\:)}\:\:\:\:\:\:\:\:\:\:\:\:\:\:\:\:\:\:\:\:for\:2-bit\end{array}\right.$$

where $\:{\upbeta\:}>1\:$is a correction factor. While we could set$\:\:{\upbeta\:}=\:1.05$, it is better channel estimation results are obtained when a value slightly larger than one is used.

### Channel estimation with $\:\varSigma\:\varDelta\:-$ ADC

By representing the input-output relationship of the quantizer through an equivalent linear model using Bussgang decomposition, we can derive closed-form expressions for the quantization noise. Building on the results from Sect.  3.1, which define the channel model and its linear equivalent, we now proceed to analyze the performance of the receiver.

#### MRC-GSIC receivers with perfect CSI

At the BS, linear receiver filters employing MRC-GSIC are used to estimate the transmitted signals for data recovery. Under the assumption of perfect CSI, the estimated signal for UE $\:k$is expressed as:

34 $$\:{\mathbf{g}}_{\mathrm{k}}^{\mathrm{M}\mathrm{R}\mathrm{C}-\mathrm{G}\mathrm{S}\mathrm{I}\mathrm{C}}{={\left[{\mathbf{G}}_{{g}_{n}}\right]}_{:\:,\:\:\mathrm{k}-\left[\right({g}_{n}-1\left)\mathrm{G}\right]\:\:}}_{\:}$$

Where $\:{\mathbf{G}}_{{g}_{n}}{=\left[\mathbf{G}\right]}_{:,({g}_{n}-1)\:\mathrm{G}+1:\mathrm{K}\:}{\in\:\mathrm{c}\:}^{M\times\:[\mathrm{K}-\mathrm{G}({g}_{n}-1\left)\right]}$ and [⋅]:_,k_ denotes extracting the *k-*th column From a matrix. With MRC-GSIC, the estimated signal in Eq. (11) is thus obtained accordingly.

35 $$\:{\widehat{\mathrm{s}}}_{\mathrm{k}}=\:{\mathbf{g}}_{\mathrm{k}}^{H}\:{\mathrm{y}}_{\mathrm{q}}$$

where $\:{\mathbf{g}}_{\mathrm{k}}$ denotes the receiver filter for UE *k*. Let $\:{{{\rm\:Y}}}_{\mathrm{k}}\:$represent the signal-to-interference-plus-noise ratio (SINR) at the BS. The uplink gross transmission rate $\:{\stackrel{-}{\mathrm{R}}}_{\mathrm{k}}$ (in bit/s) For UE *k* with bandwidth B is given by:

36 $$\:{\stackrel{-}{\mathrm{R}}}_{\mathrm{k}}=\:\mathrm{B}\:{\stackrel{-}{\mathrm{S}\mathrm{E}}}_{\mathrm{k}}$$

where $\:{\stackrel{-}{\mathrm{S}\mathrm{E}}}_{\mathrm{k}}$ denotes the uplink gross SE for UE *k*, which is defined as

37 $$\:{\stackrel{-}{\mathrm{S}\mathrm{E}}}_{\mathrm{k}}\:=\:\mathrm{l}\mathrm{o}\mathrm{g}(1\:+\:{{\upgamma\:}}_{\mathrm{k}}\:)$$

The SINR For UE *k* is evaluated as:

38 $$\:{{\upgamma\:}}_{\mathrm{k}}=\:\frac{{{\mathrm{a}}^{2}\:\:\mathrm{P}}_{\mathrm{k}}\:{{\Vert\:\mathbf{g}}_{\mathrm{k}}\Vert\:}^{4}}{{\mathrm{I}}_{\mathrm{k}}}\:$$

The detected signal $\:{\widehat{\mathrm{s}}}_{\mathrm{k}}$ is given by:

39 $$\:{\widehat{\mathrm{s}}}_{\mathrm{k}}\:=\:{a}_{\mathrm{m}{\prime\:}}\:\:{{\Vert\:\mathbf{g}}_{\mathrm{k}}\Vert\:}^{2}\:{\mathrm{s}}_{\mathrm{k}}\:+\:\:{a}_{\mathrm{m}{\prime\:}}\:{\mathbf{g}}_{\mathrm{k}}^{H}\:\mathrm{v}+\:{\mathbf{g}}_{\mathrm{k}}^{H}{{{\:\mathbf{R}}_{\mathbf{v}}}_{\mathrm{q}}\:\mathbf{g}}_{\mathrm{k}}+\:{a}_{\mathrm{m}{\prime\:}}{\sum\:}_{\begin{array}{c}i=\left({g}_{n}-1\right)G\:\\\:i\ne\:k\end{array}}^{\mathrm{K}}{{\mathbf{g}}_{\mathrm{k}}^{H}\:\mathbf{g}}_{\mathrm{i}}{\:\mathrm{s}}_{\mathrm{i}}$$

where $\:{I}_{k}\:$denotes the distortion-plus-noise power and it is expressed as:

40 $$\:{\mathrm{I}}_{\mathrm{k}}{=a}_{m{\prime\:}}^{2}\:\:{{\Vert\:\mathbf{g}}_{\mathrm{k}}\Vert\:}^{2}\:{{\upsigma\:}}^{2}+\:{\mathbf{g}}_{\mathrm{k}}^{H}\:{{\:\mathbf{R}}_{\mathbf{v}}}_{\mathrm{q}}{\:\mathbf{g}}_{\mathrm{k}}+{a}_{m{\prime\:}}^{2}{\sum\:}_{\begin{array}{c}i=({g}_{n}-1)G+1\:\\\:,i\ne\:k\end{array}}^{\mathrm{N}}{\left|{\mathbf{g}}_{k}^{H}{\:\mathbf{g}}_{\mathrm{i}\:}\right|}^{2}{\:\mathrm{P}}_{\mathrm{i}}$$

The first term on the right-hand side (RHS) of Eq. (40) represents the channel noise power, the second term corresponds to the ADC quantization noise power, and the last term accounts for the IUI. For MRC-GSIC receivers, the power of the ADC quantization noise can be approximated as

41 $$\:{\mathbf{g}}_{\mathrm{k}}^{H}{{{\:\mathbf{R}}_{\mathbf{v}}}_{\mathrm{q}}\:\mathbf{g}}_{\mathrm{k}}={a}_{\mathrm{m}{\prime\:}}\:(1-\:{a}_{\mathrm{m}{\prime\:}})\:{\mathbf{g}}_{\mathrm{k}}^{H}\:\mathrm{d}\mathrm{i}\mathrm{a}\mathrm{g}\:({\mathrm{P}}_{\mathrm{s}}\:\mathbf{G}{\:\mathbf{G}}^{\mathrm{H}}+{{\upsigma\:}}^{2})\:{\mathbf{g}}_{\mathrm{k}}$$

Then, we complete the last term in (40). The *m-*th diagonal element $\:\mathrm{d}\mathrm{i}\mathrm{a}\mathrm{g}({\mathrm{p}}_{\mathrm{s}}\:\mathbf{G}{\:\mathbf{G}}^{\mathrm{H}}+\:{\sigma\:}^{2})$ can be calculated by $\:\mathrm{d}\mathrm{i}\mathrm{a}\mathrm{g}({\mathrm{P}}_{\mathrm{s}}\:\mathbf{G}{\:\mathbf{G}}^{\mathrm{H}}+\:{{\upsigma\:}}^{2})=\:{\mathrm{P}}_{\mathrm{s}}\{\:{\sum\:}_{\mathrm{i}=1}^{\mathrm{K}}{\:\parallel\:\:{\mathbf{g}}_{\mathrm{m}\mathrm{i}}\:\parallel\:}^{2}+\:{{\upsigma\:}}^{2}\}$, where $\:{\mathbf{g}}_{\mathrm{m}\mathrm{i}}$ is the *m-th* row and *i-*th column entry of the channel matrix $\:\mathbf{G}$. It donates the total average power at the input of the m-th ADC, given a channel realization $\:\mathbf{G}$, the noise-plus-interference term is given by:

42 $$\:{\mathbf{g}}_{\mathrm{k}}^{H}\:{{{\:\mathbf{R}}_{\mathbf{v}}}_{\mathrm{q}}\:\mathbf{g}}_{\mathrm{k}}={a}_{\mathrm{m}{\prime\:}}\:(1\:-\:{a}_{\mathrm{m}{\prime\:}})\:{\sum\:}_{\mathrm{k}=1}^{\mathrm{K}}{\:\parallel\:\:{\mathbf{g}}_{\mathrm{k}}\:\parallel\:}^{2}+\:{\mathrm{P}}_{\mathrm{s}}\{\:{\sum\:}_{\mathrm{m}=1}^{\mathrm{M}}{\:\parallel\:\:{\mathbf{g}}_{\mathrm{m}\mathrm{i}}\:\parallel\:}^{2}+\:{{\upsigma\:}}^{2}\}$$

Substituting the approximations into Eq. (38), we can approximate $\:{\mathrm{I}}_{\mathrm{k}}$ as:

43 $$\:{\mathrm{I}}_{\mathrm{k}}{\:={a}_{m{\prime\:}}^{2}\parallel\:\:{\mathbf{g}}_{\mathrm{k}}\:\:\parallel\:}^{2}{{\upsigma\:}}^{2}+{a}_{m{\prime\:}}^{2}\:{\sum\:}_{\begin{array}{c}i=({\mathrm{g}}_{\mathrm{n}}-1)G+1\\\:\:i\ne\:k\end{array}}^{\mathrm{K}}{{|\mathbf{g}}_{\mathrm{k}}^{H}\:{\mathbf{g}}_{\mathrm{i}\:}|}^{2}\:{\mathrm{P}}_{\mathrm{i}}\:+\:{a}_{\mathrm{m}{\prime\:}}\:(1-\:{a}_{\mathrm{m}{\prime\:}})\:{\mathrm{P}}_{\mathrm{s}}{\sum\:}_{\mathrm{i}=1}^{\mathrm{K}}{\:\parallel\:\:{\mathbf{g}}_{\mathrm{k}}\:\parallel\:}^{2}\{\:{\sum\:}_{\mathrm{m}=1}^{\mathrm{M}}{\:\parallel\:\:{\mathbf{g}}_{\mathrm{m}\mathrm{i}}\:\parallel\:}^{2}+\:{{\upsigma\:}}^{2}\}.$$

Define $\:{\mathbf{Q}\in\:\mathbb{C}}^{K\times\:K}$, $\:{\mathrm{Q}}_{\mathrm{k}\mathrm{i}}\triangleq\:{\sum\:}_{\mathrm{m}=1}^{\mathrm{M}}{\left|{\mathbf{g}}_{\mathrm{k}\mathrm{m}}^{\mathrm{*}}\:{\mathbf{g}}_{\mathrm{m}\mathrm{i}}\right|}^{2}$ whose (*k*,* i*)-th entry, where $\:{\mathbf{g}}_{\mathrm{k}\mathrm{m}}$ is the *m-*th entry of $\:{\mathbf{g}}_{\mathrm{k}}$, $\:{\mathbf{g}}_{\mathrm{m}\mathrm{i}\:}$is the *m*-th entry of $\:{\mathbf{g}}_{\mathrm{i}}$, and * is the complex conjugate. Then, $\:{I}_{k}$ can alternatively be expressed as:

44 $${{\mathrm{I}}_{\mathrm{k}}}=\underbrace {{{{ }}a_{{m'}}^{2}{{}}{{{\boldsymbol{\upsigma}}}^2}\parallel {{\mathbf{g}}_{\mathrm{k}}}{{~}}{\parallel ^2}}}_{{{\text{channel noise }}}}+\underbrace {{a_{{m'}}^{2}{{~}}\mathop \sum \limits_{{\begin{array}{*{20}{c}} {i=\left( {{g_n} - 1} \right)G+1} \\ {~i \ne k} \end{array}}}^{{\mathrm{K}}} |{\mathbf{g}}_{{\mathrm{k}}}^{H}~{{\mathbf{g}}_{{\mathrm{i}}}}{|^2}{{~}}{{\mathrm{P}}_{\mathrm{i}}}}}_{{{\mathrm{inter}} - {\mathrm{UE~interference}}}}+\underbrace {{{a_{{\mathrm{m'}}}}{{~}}\left( {1 - {{~}}{a_{{\mathrm{m'}}}}} \right){{~}}{{\mathrm{P}}_{\mathrm{s}}}\mathop \sum \limits_{{{\mathrm{i}}=1}}^{{\mathrm{K}}} {{\mathrm{Q}}_{{\mathrm{ki}}}}}}_{{{\text{ADC quantization noise }}}}$$

#### ZF-GSIC receivers with perfect CSI

It is known that the ZF receiver can fully mitigate the inter-UE interference. Thus, the estimated signal in Eq. (10) can be expressed as:

45 $${\mathbf{g}}_{{\mathrm{k}}}^{{{\mathrm{ZF}} - {\mathrm{GSIC}}}}={\left[ {G_{{{g_n}}}^{\dag }} \right]_{:,{\mathrm{~k}} - \left[ {\left( {{g_n} - 1} \right){\mathrm{G}}} \right]}}~$$

The ZF filter is corresponding to the Moore-Penrose pseudo-inverse of $\:{\boldsymbol{G}}_{{g}_{n}}$, where ${{\mathbf{G}}_{{g_n}}}^{\dag }={\mathbf{G}}_{{{g_n}}}^{{\mathrm{H}}}{({{\mathbf{G}}_{{g_n}}}{\mathrm{~}}{\mathbf{G}}_{{{g_n}}}^{{\mathrm{H}}})^{ - 1}},~G_{{{g_n}}}^{\dag }={\left[ {\mathbf{G}} \right]_{:,\left( {{g_n} - 1} \right){\mathrm{~G}}+1:{\mathrm{K~}}}} \in {\mathrm{c}}{{\mathrm{~}}^{{\mathrm{M}} \times \left[ {{\mathrm{K}} - {\mathrm{G}}\left( {{g_n} - 1} \right)} \right]}}~~$ and [⋅]:,k denotes extracting the *k-*th column of a matrix. With ZF-GSIC, the estimated signal in Eq. (10) can be written as:

46 $$\:{\mathrm{s}}_{\mathrm{k}}\:=\:{\mathbf{g}}_{\mathrm{k}}^{H}\:\:{\mathbf{y}}_{\mathrm{q}}$$

47 $$\:{\widehat{\mathrm{s}}}_{\mathrm{k}}\:={a}_{m{\prime\:}}\:\sqrt{{\mathrm{p}}_{\mathrm{s}}}\:{{\Vert\:\mathbf{g}}_{\mathrm{k}}\Vert\:}^{2}\:{\mathrm{s}}_{\mathrm{k}}\:+\:\:{a}_{m{\prime\:}}\:\:{\mathbf{g}}_{\mathrm{k}}^{H}\:\:\mathrm{v}+\:{\mathbf{g}}_{\mathrm{k}}^{\mathrm{H}}\:\:{{{\:\mathbf{R}}_{\mathbf{v}}}_{\mathrm{q}}\:\mathbf{g}}_{\mathrm{k}\:}$$

The SINR For UE *k* is evaluated as:

48 $$\:{{{\rm\:Y}}}_{\mathrm{k}}\:=\:\frac{{a}_{m{\prime\:}}^{2}\:{\mathrm{P}}_{\mathrm{k}}\:}{{\mathbf{I}}_{\mathrm{k}}}\:$$

where $\:{I}_{k}\:$denotes the distortion-plus-noise power and it may be expressed as

49 $$\:{\mathrm{I}}_{\mathrm{k}}={\mathrm{a}}^{2}\:\:{{\Vert\:\mathbf{g}}_{\mathrm{k}}\Vert\:}^{2}\:{{\upsigma\:}}^{2}+\:{\mathbf{g}}_{\mathrm{k}}^{H}\:{{{\:\mathbf{R}}_{\mathbf{v}}}_{\mathrm{q}}\:\mathbf{g}}_{\mathrm{k}}$$

The first term on the RHS of Eq. (49) represents the channel noise power, while the second term accounts for the ADC noise of quantization. Using the previous approximations for the ADC quantization noise, $\:{I}_{k}$ can be further expressed as:

50 $$\:{\mathrm{I}}_{\mathrm{k}}{\:={a}_{m{\prime\:}}^{2}\:\parallel\:\:{\mathbf{g}}_{\mathrm{k}}\:\:\parallel\:}^{2}{{\upsigma\:}}^{2}+\:{a}_{\mathrm{m}{\prime\:}}(1-\:{a}_{\mathrm{m}{\prime\:}}){\mathrm{P}}_{\mathrm{s}}\:{\sum\:}_{\mathrm{i}=1}^{\mathrm{K}}{\:\parallel\:\:{\mathbf{g}}_{\mathrm{k}}\:\parallel\:}^{2}\{\:{\sum\:}_{\mathrm{m}=1}^{\mathrm{M}}{\:\parallel\:\:{\mathbf{g}}_{\mathrm{i}}\:\parallel\:}^{2}+\:{{\upsigma\:}}^{2}\}$$

and

51 $$\:{\mathrm{Q}}_{\mathrm{k}\mathrm{i}}\triangleq\:{\sum\:}_{\mathrm{m}=1}^{\mathrm{M}}{\left|{\mathbf{g}}_{\mathrm{k}\mathrm{m}}^{\mathrm{*}}\:\:{\mathbf{g}}_{\mathrm{m}\mathrm{i}}\right|}^{2}$$

52 $$\:{\mathrm{I}}_{\mathrm{k}}={\:{a}_{m{\prime\:}}^{2}\:\:{{\upsigma\:}}^{2}\parallel\:\:{\mathbf{g}}_{\mathrm{k}}\:\parallel\:}^{2}+{a}_{\mathrm{m}{\prime\:}}\:(1-\:{a}_{\mathrm{m}{\prime\:}})\:{\mathrm{P}}_{\mathrm{s}}{\sum\:}_{\mathrm{i}=1}^{\mathrm{K}}{\mathrm{Q}}_{\mathrm{k}\mathrm{i}\:.}$$

#### MRC-GSIC receivers with imperfect CSI

The data transmission follows the training phase. Let $\:{\widehat{{\upgamma\:}}}_{k}$ denote the SINR with imperfect CSI. The uplink gross transmission rate $\:{\stackrel{-}{\mathrm{R}}}_{\mathrm{k}}$ (in bit/s) For UE *k* with bandwidth *B* is

53 $$\:{\stackrel{-}{\mathrm{R}}}_{\mathrm{k}}=\:\mathrm{B}\:{\stackrel{-}{\mathrm{S}\mathrm{E}}}_{\mathrm{k}}$$

where $\:{\stackrel{-}{\mathrm{S}\mathrm{E}}}_{\mathrm{k}}$ denotes the uplink gross spectral efficiency (SE) For UE *k* and is defined.

as

54 $$\:{\stackrel{-}{\mathrm{S}\mathrm{E}}}_{\mathrm{k}}\:=\:\mathrm{l}\mathrm{o}\mathrm{g}(1\:+\:{\widehat{{\upgamma\:}}}_{k}\:)$$

We now discuss SINR with imperfect CSI as $\:{\widehat{{\upgamma\:}}}_{k}$ The SINR $\:{\widehat{{\upgamma\:}}}_{k}$ with MRC-GSIC receivers, it can be expressed as:

55 $$\:{\widehat{{\upgamma\:}}}_{k}=\frac{{a}_{m{\prime\:}}^{2}\:{\mathrm{P}}_{\mathrm{k}}\:{\Vert\:{\:\widehat{\mathbf{g}}}_{\mathrm{k}}\Vert\:}^{4}}{{\:\widehat{\mathrm{I}}}_{\mathrm{k}}}$$

At the BS, linear receiver filters are employed to estimate the transmitted signals for recovering data. Assuming imperfect CSI, the estimated signal for UE *k* is $\:{\widehat{\mathrm{s}}}_{\mathrm{n}}\:=\:\:{{\widehat{\mathbf{g}}}_{\mathrm{k}}^{\mathrm{H}}\:\widehat{y}}_{\mathrm{q}}$, then it can be written as:

56 $$\:{\widehat{\mathrm{s}}}_{\mathrm{k}}=\:{\widehat{\mathbf{g}}}_{\mathrm{k}}^{\mathrm{H}}\:({a}_{\mathrm{m}{\prime\:}}\:\sqrt{{\mathbf{p}}_{\mathbf{s}}\:}\:{{\widehat{\mathrm{g}}}_{\mathrm{k}}\:\mathrm{s}}_{\mathrm{k}}+{a}_{\mathrm{m}{\prime\:}}\:\sqrt{{\mathbf{p}}_{\mathbf{s}}\:}\:{\sum\:}_{\mathrm{i}=1\:,\mathrm{i}\ne\:\mathrm{k}}^{\mathrm{K}}\:{\widehat{\mathrm{g}}}_{\mathrm{k}}{\mathrm{s}}_{\mathrm{k}}+{a}_{\mathrm{m}{\prime\:}}\:\mathbf{v}+{\:\mathbf{v}}_{\mathrm{q}}$$

Let $\:{\mathbf{e}}_{\mathrm{k}}={\mathbf{g}}_{\mathrm{k}}-{\widehat{\mathbf{g}}}_{\mathrm{k}}\:\:$denote the channel estimation error, where $\:{\mathbf{g}}_{\mathrm{k}}$ is the estimated channel vector and $\:{\mathbf{g}}_{\mathrm{k}}$​ is the true channel vector. Then, after linear reception, we have:

57 $$\:{\widehat{\mathrm{s}}}_{\mathrm{k}}={\widehat{\mathbf{g}}}_{\mathrm{k}}^{\mathrm{H}}({a}_{\mathrm{m}{\prime\:}}\sqrt{{\mathrm{P}}_{\mathrm{s}}}\:{[\widehat{\mathbf{g}}+\widehat{\mathbf{e}}\:]\:\mathrm{s}}_{\mathrm{k}}\:+{a}_{\mathrm{m}{\prime\:}}{\sum\:}_{\begin{array}{c}i=\left({g}_{n}-1\right)G+1\:\\\:,i\ne\:k\end{array}}^{\mathrm{K}}{\widehat{\mathbf{g}}}_{\mathrm{i}}{\mathrm{s}}_{\mathrm{i}}+{a}_{\mathrm{m}{\prime\:}}\:\mathbf{v}+{\mathbf{v}}_{\mathrm{q}}$$

Expanding Eq. (57), we get:

58 $$\:{\widehat{\mathrm{s}}}_{\mathrm{k}}\:=\:{a}_{\mathrm{m}{\prime\:}}\:\sqrt{{\mathrm{P}}_{\mathrm{s}}}\:{\Vert\:{\widehat{\mathbf{g}}}_{\mathrm{k}}\Vert\:}^{2}\:{\mathrm{s}}_{\mathrm{k}}\:+\:{a}_{\mathrm{m}{\prime\:}}\:\sqrt{{\mathrm{P}}_{\mathrm{s}}}{\sum\:}_{\mathrm{i}=1}^{\mathrm{K}}{\widehat{\mathbf{g}}}_{\mathrm{k}}^{\mathrm{H}}{\widehat{e}}_{\mathrm{i}}{\mathrm{s}}_{\mathrm{k}}+\:{a}_{\mathrm{m}{\prime\:}}\:{\sum\:}_{\begin{array}{c}i=\left({g}_{n}-1\right)G+1\\\:\:,i\ne\:k\end{array}}^{\mathrm{K}}{{\widehat{\mathbf{g}}}_{\mathrm{k}}^{\mathrm{H}}\:\widehat{\mathbf{g}}}_{\mathrm{i}}\:{\mathrm{s}}_{\mathrm{i}}+{a}_{\mathrm{m}{\prime\:}}\:{\widehat{\mathbf{g}}}_{\mathrm{k}}^{\mathrm{H}}\:\mathbf{v}+\:{\widehat{\mathbf{g}}}_{\mathrm{k}}^{\mathrm{H}}\:{{\:\mathbf{R}}_{\mathbf{v}}}_{\mathrm{q}}{\widehat{\mathbf{g}}}_{\mathrm{k}}$$

where $\:{I}_{k}\:$denotes the distortion-plus-noise power, expressed as:

59 $${{{\hat I}}_{\mathrm{k}}} = a_{m'}^2{\left\| {{{{\hat{\mathbf g}}}_{\mathrm{k}}}} \right\|^2}{}{{{\sigma }}^2} + a_{m'}^2{{\mathrm{P}}_{\mathrm{s}}}\mathop \sum \limits_{\begin{matrix} {i = \left( {{{\mathrm{g}}_{\mathrm{n}}} - 1} \right)G + 1\;} \\ {,i \ne k} \\ \end{matrix} }^{\mathrm{K}} \mid {\hat{\mathbf g}}_{\mathrm{k}}^{\mathrm{H}}{{\hat{\mathbf g}}_i}{|^2} + a_{m'}^2{\left\| {{{{\hat{\mathbf g}}}_{\mathrm{k}}}} \right\|^2}\mathop \sum \limits_{{\mathrm{i}} = 1}^{\mathrm{K}} {{{\sigma }}^2}_{{\mathrm{ei}}}{{\mathrm{P}}_{\mathrm{i}}} + {\hat{\mathbf g}}_{\mathrm{k}}^{\mathrm{H}}\;{{\bf{R}}_{\bf{v}}}_{\mathrm{q}}{{\hat{\mathbf g}}_{\mathrm{k}}}$$

The first term on the right-hand side (RHS) of Eq. (59) represents the channel noise power, the second term represents the inter-UE interference power (IUI), the third term corresponds to the ADC quantization noise power, and the last term accounts for the channel estimation error. Furthermore, for MRC–GSIC receivers, the ADC quantization noise power can be approximated as:

60 $$\:{\widehat{\mathbf{g}}}_{\mathrm{k}}^{\mathrm{H}}{{\:\mathbf{R}}_{\mathbf{v}}}_{\mathrm{q}}{\widehat{\mathbf{g}}}_{\mathrm{k}}=\:{a}_{\mathrm{m}{\prime\:}}(1\:-\:{a}_{\mathrm{m}{\prime\:}})\:\:\mathrm{d}\mathrm{i}\mathrm{a}\mathrm{g}\:\:{\widehat{\mathbf{g}}}_{\mathrm{k}}^{\mathrm{H}}\:{\mathrm{p}}_{\mathrm{s}}{\left[\right(\:\widehat{\mathbf{g}}}_{\mathrm{k}}+{\widehat{\mathbf{e}}}_{\mathrm{k}}\left){{(\:\widehat{\mathbf{g}}}_{\mathrm{k}}+{\widehat{\mathbf{e}}}_{\mathrm{k}})}^{\mathbf{H}}\right]+{{\upsigma\:}}^{2}\}{\mathbf{g}}_{\mathrm{k}}$$

61 $$\:{\widehat{\mathbf{g}}}_{\mathrm{k}}^{\mathrm{H}}\:{{\mathbf{R}}_{\mathbf{v}}}_{\mathrm{q}}{\widehat{\mathbf{g}}}_{\mathrm{k}}={a}_{\mathrm{m}{\prime\:}}(1\:-\:{a}_{\mathrm{m}{\prime\:}})\{{\sum\:}_{\mathrm{i}=1}^{\mathrm{K}}{\Vert\:\widehat{\mathbf{g}}}_{\mathrm{k}}\Vert\:\:{\mathrm{p}}_{\mathrm{s}}[{\sum\:}_{\mathrm{m}=1}^{\mathrm{M}}{{\Vert\:\widehat{\mathbf{g}}}_{\mathrm{m}\mathrm{i}}\Vert\:}^{2}+\Vert\:{{\widehat{\mathbf{e}}}_{\mathrm{k}}\Vert\:}^{2}+\:{\:\widehat{\mathbf{g}}}_{\mathrm{k}\:}{\widehat{\mathbf{e}}}_{\mathrm{k}}^{\mathrm{H}}+\:{\widehat{\mathbf{g}}}_{\mathrm{k}}^{\mathrm{H}}{\widehat{\mathbf{e}}}_{\mathrm{k}}]+{{\upsigma\:}}^{2}\}$$

Here, we use the fact that the estimated channel $\:{\:\mathbf{g}}_{\mathrm{k}}\:$and the channel estimation error $\:{\:\mathbf{e}}_{\mathrm{k}}$ are uncorrelated:$\:\:{\:\mathbb{E}\{\widehat{\mathbf{g}}}_{\mathrm{k}}\:{\widehat{\mathbf{e}}}_{\mathrm{k}}^{\mathrm{H}}\}=\:0$,$\:{\:\mathbb{E}\{\widehat{\mathbf{e}}}_{\mathrm{k}}\:{\widehat{\mathbf{g}}}_{\mathrm{k}}^{\mathrm{H}}\}=0$, $\:{\:\mathbb{E}\{\Vert\:\widehat{\mathbf{e}}}_{\mathrm{k}}\Vert\:\}={{{\upsigma\:}}^{2}}_{\mathrm{e}\mathrm{i}}$

62 $$\:{\widehat{\mathbf{g}}}_{\mathrm{k}}^{\mathrm{H}}\:{{\mathbf{R}}_{\mathbf{v}}}_{\mathrm{q}}{\widehat{\mathbf{g}}}_{\mathrm{k}}={a}_{\mathrm{m}{\prime\:}}\:(1\:-\:\:{a}_{\mathrm{m}{\prime\:}})\:{\sum\:}_{\mathrm{i}=1}^{\mathrm{K}}{{\Vert\:\widehat{\mathbf{g}}}_{\mathrm{k}}\Vert\:}^{2}\left\{{\mathrm{P}}_{\mathrm{s}}\:\right[\:{\sum\:}_{\mathrm{m}=1}^{\mathrm{M}}{{\Vert\:\widehat{\mathbf{g}}}_{\mathrm{m}\mathrm{i}}\Vert\:}^{2}+\:{{{\upsigma\:}}^{2}}_{\mathrm{e}\mathrm{i}}]+\:{{\upsigma\:}}^{2}\}$$

Substituting the approximations into Eq. (59), $\:{\:\widehat{\mathrm{I}}}_{\mathrm{k}}$can be rewritten as:

63 $${{{\hat {I}}}_{\mathrm{k}}}=a_{{m'}}^{2}{{~}}{\left\| {{{{\mathbf{\hat {g}}}}_{\mathrm{k}}}} \right\|^2}\left. {{{{\boldsymbol{\upsigma}}}^2}+{a_{{{m^{\prime}}}}}{{~}}\left( {1{{~}} - {a_{{{m^{\prime}}}}}} \right)} \right||{{~}}\left\| {{{{\hat{\mathbf {g}}}}_{\mathrm{k}}}} \right\|{{~}}{{{\boldsymbol{\upsigma}}}^2}+{{~}}a_{{m'}}^{2}\mathop \sum \limits_{{\begin{array}{*{20}{c}} {i=\left( {{g_n} - 1} \right)G+1} \\ {~,i \ne k} \end{array}}}^{{\mathrm{K}}} {\left| {{\hat{\mathbf {g}}}_{{\mathrm{k}}}^{H}~{{{\hat{\mathbf {g}}}}_{\mathrm{i}}}} \right|^2}{{\mathrm{P}}_{\mathrm{i}}}+a_{{m}}^{\prime 2}{\left\| {{{{\hat{\mathbf {g}}}}_{\mathrm{k}}}} \right\|^2}\mathop \sum \limits_{{{\mathrm{i}}=1}}^{{\mathrm{K}}} {{{\boldsymbol{\upsigma}}}^2}_{{{\mathrm{ei}}}}{{\mathrm{P}}_{\mathrm{i}}}{{~}}$$

and

64 $$\:{\:\widehat{\mathrm{Q}}}_{\mathrm{k}\mathrm{i}}\triangleq\:{\sum\:}_{\mathrm{m}=1}^{\mathrm{M}}\left|{{\widehat{\mathbf{g}}}_{\mathrm{k},\mathrm{m}}^{\mathrm{*}}|}^{2}\right(\left|{\:\widehat{\mathbf{g}\:}}_{\mathrm{m},\mathrm{i}}\right|+{{{\upsigma\:}}^{2}}_{\mathrm{e}\mathrm{i}})$$

65 $${{{\hat {I}}}_{\mathrm{k}}}=\underbrace {{a_{{m \prime}}^{2}~{{\left\| {{{\widehat {{{\mathbf{g}}~}}}_{\mathrm{k}}}} \right\|}^2}{{~}}{{{\boldsymbol{\upsigma}}}^2}}}_{{{\text{channel noise}},}}+\underbrace {{{a_{{\mathrm{m}}\prime}}\left( {1 - {{~}}{a_{{{m \prime}}}}} \right){{~}}\mathop \sum \limits_{{{\mathrm{i}}=1}}^{{\mathrm{N}}} {{~}}{{{{\hat {Q}}}}_{{\mathrm{ki}}}}{{\mathrm{p}}_{\mathrm{i}}}}}_{{{\text{ADC quantization noise}}}}+\underbrace {{a_{{m\prime}}^{2}{{~}}\mathop \sum \limits_{{\begin{array}{*{20}{c}} {i=\left( {{g_n} - 1} \right)G+1~} \\ {,i \ne k} \end{array}}}^{{\mathrm{K}}} {{\left| {{\hat{\mathbf {g}}}_{{\mathrm{k}}}^{{\mathrm{H}}}~{{{\hat{\mathbf {g}}}}_{\mathrm{i}}}} \right|}^2}{\mathrm{pi}}}}_{{{\mathrm{inter}} - {\text{UE interference}}}}+\underbrace {{a_{{m\prime }}^{2}{{\left\| {{{{\hat{\mathbf {g}}}}_{\mathrm{k}}}} \right\|}^2}\mathop \sum \limits_{{{\mathrm{i}}=1}}^{{\mathrm{K}}} {{{\boldsymbol{\upsigma}}}^2}_{{{\mathrm{ei}}}}{{\mathrm{P}}_{\mathrm{i}}}}}_{{{\text{channel estimation error}}}}$$

#### ZF-GSIC receivers with imperfect CSI

Let $\:{\widehat{{\upgamma\:}}}_{k}$ denote the SINR with imperfect CSI. For MRC-GSIC receivers, the SINR is given by:

66 $$\:{\widehat{{\upgamma\:}}}_{k}=\frac{{a}_{m{\prime\:}}^{2}\:{\mathrm{P}}_{\mathrm{k}}\:}{{\:\widehat{\mathrm{I}}}_{\mathrm{k}}}$$

ZF receivers generally outperform MRC in terms of interference suppression. When ZF receivers are used, the filter for UE *k* is given by:

67 $$\:{\widehat{\mathrm{s}}}_{\mathrm{n}}\:=\:{\widehat{\mathbf{g}}}_{\mathrm{k}}^{\mathrm{H}}\:{\mathbf{y}}_{\mathrm{q}}$$

68 $$\:{\widehat{\mathrm{s}}}_{\mathrm{k}}=\:{\widehat{\mathbf{g}}}_{\mathrm{k}}^{\mathrm{H}}\:({a}_{\mathrm{m}{\prime\:}}\sqrt{{\mathrm{p}}_{\mathrm{s}}}\:{{\widehat{\mathbf{g}}}_{\mathrm{k}}\:\mathrm{s}}_{\mathrm{k}}+{a}_{\mathrm{m}{\prime\:}}\:\mathbf{v}\:+{\:\mathrm{n}}_{\mathrm{v}})$$

69 $$\:{\widehat{\mathrm{s}}}_{\mathrm{k}}\:=\:{\widehat{\mathbf{g}}}_{\mathrm{k}}^{\mathrm{H}}\:({a}_{\mathrm{m}{\prime\:}}\sqrt{{\mathrm{P}}_{\mathrm{s}}}\:{[\widehat{\mathbf{g}}\:+\:\widehat{\mathbf{e}}]\:\mathrm{s}}_{\mathrm{k}}\:+{a}_{\mathrm{m}{\prime\:}}\:\mathbf{v}\:+{\mathbf{v}}_{\mathrm{q}})$$

70 $$\:{\widehat{\mathrm{s}}}_{\mathrm{k}}=\:{a}_{\mathrm{m}{\prime\:}}\:\sqrt{{\mathrm{P}}_{\mathrm{s}}}\:{\Vert\:{\widehat{\mathbf{g}}}_{\mathrm{k}}\Vert\:}^{2}\:{\mathrm{s}}_{\mathrm{k}}\:+\:{a}_{\mathrm{m}{\prime\:}}\:\sqrt{{\mathrm{p}}_{\mathrm{s}}}{\sum\:}_{\mathrm{i}=1}^{\mathrm{K}}{\widehat{\mathbf{g}}}_{\mathrm{k}}{{\mathrm{e}}_{\mathrm{i}}\:\mathrm{s}}_{\mathrm{i}}+\:{a}_{\mathrm{m}{\prime\:}}\:{\widehat{\mathbf{g}}}_{\mathrm{k}}^{\mathrm{H}}\:\:\mathbf{v}+\:{\widehat{\mathbf{g}}}_{\mathrm{k}}^{\mathrm{H}}{{\:\mathbf{R}}_{\mathbf{v}}}_{\mathrm{q}}{\widehat{\mathbf{g}}}_{\mathrm{k}}\:$$

Where$\:{\:\widehat{\mathrm{I}}}_{\mathrm{k}}\:$represents the power of the errors due to imperfect channel estimation, the channel noise, ADC quantization noise is calculated as follows:

71 $$\:{\widehat{\mathrm{I}}}_{\mathrm{k}}=\Vert\:{\:\widehat{\mathbf{g}}}_{\mathrm{k}}{\Vert\:}^{2}\:{{\upsigma\:}}^{2}+{a}_{m{\prime\:}}^{2}{\Vert\:{\:\widehat{\mathbf{g}\:}}_{\mathrm{k}}\Vert\:}^{2}{\sum\:}_{\mathrm{i}=1}^{\mathrm{K}}{{{\upsigma\:}}^{2}}_{\mathrm{e}\mathrm{i}}\:{\mathrm{p}}_{\mathrm{i}}+\:{\widehat{\mathbf{g}}}_{\mathrm{k}}^{\mathrm{H}}{{\:\mathbf{R}}_{\mathbf{v}}}_{\mathrm{q}}{\widehat{\mathbf{g}}}_{\mathrm{k}}$$

72 $$\:{\widehat{\mathrm{I}}}_{\mathrm{k}}={a}_{m{\prime\:}}^{2}\:\Vert\:{\:\widehat{\mathbf{g}}}_{\mathrm{k}}{\Vert\:}^{2}\:{{\upsigma\:}}^{2}+{a}_{m{\prime\:}}^{2}{\Vert\:{\:\widehat{\mathbf{g}\:}}_{\mathrm{k}}\Vert\:}^{2}{\sum\:}_{\mathrm{i}=1}^{\mathrm{K}}{{{\upsigma\:}}^{2}}_{\mathrm{e}\mathrm{i}}\:{\mathrm{p}}_{\mathrm{i}}+\:{a}_{\mathrm{m}{\prime\:}}(1-{a}_{\mathrm{m}{\prime\:}})\:{\sum\:}_{\mathrm{i}=1}^{\mathrm{K}}{{\Vert\:\widehat{\mathbf{g}}}_{\mathrm{k}}\Vert\:}^{2}\{{\mathrm{P}}_{\mathrm{s}}{\sum\:}_{\mathrm{m}=1}^{\mathrm{M}}{{\Vert\:\widehat{\mathbf{g}}}_{\mathrm{m},\mathrm{i}}\Vert\:}^{2}+{{{\upsigma\:}}^{2}}_{\mathrm{e}\mathrm{i}}]+{{\upsigma\:}}^{2}\}$$

Where

73 $$\:{\:\widehat{\mathrm{Q}}}_{\mathrm{k}\mathrm{i}}\triangleq\:{\sum\:}_{\mathrm{m}=1}^{\mathrm{M}}\left|{{\widehat{\mathbf{g}}}_{\mathrm{k},\mathrm{m}}^{\mathrm{*}}|}^{2}\right(\left|{\:\widehat{\mathbf{g}\:}}_{\mathrm{m},\mathrm{i}}\right|+{{{\upsigma\:}}^{2}}_{\mathrm{e}\mathrm{i}})$$

74 $$\:{{\widehat{\mathrm{I}}}_{\mathrm{k}}=a}_{m{\prime\:}}^{2}\:{\Vert\:{\:\widehat{\mathrm{g}\:}}_{\mathrm{k}}\Vert\:}^{2}\:{{\upsigma\:}}^{2}+\:{a}_{\mathrm{m}{\prime\:}}(1-\:{a}_{\mathrm{m}{\prime\:}})\:{\sum\:}_{\mathrm{i}=1}^{\mathrm{K}}{\:\widehat{\mathrm{Q}}}_{\mathrm{k}\mathrm{i}}{\mathrm{P}}_{\mathrm{i}}+{a}_{m{\prime\:}}^{2}{\Vert\:{\:\widehat{\mathbf{g}\:}}_{\mathrm{k}}\Vert\:}^{2}{\sum\:}_{\mathrm{i}=1}^{\mathrm{K}}{{{\upsigma\:}}^{2}}_{\mathrm{e}\mathrm{i}}\:{\mathrm{P}}_{\mathrm{i}\:.}$$

## Asymptotic performance and computational complexity analysis

In this section, we discuss the asymptotic analysis of SINR using random matrix theory under the assumption of perfect and imperfect CSI For massive MIMO systems, which provides insights into the effect of Rician $\:\mathcal{K}\:-$Factor, $\:{\Sigma\:}{\Delta\:}-$ ADC resolution, and the number of BS antennas on the SE. In addition, we investigate the power scaling behavior for the system with MRC-GSIC and ZF-GSIC receivers under imperfect CSI, which provides insight into the performance when the number of antennas increases. following the same consideration in^30–32^, we assume, $\:{P}_{s}={P}_{k},\forall\:k\:=1\dots\:K\:$in the following analysis. We will show the approximation of power allocation for a given QoS in the next section.

### Approximation of the transmission rate

In this section, we first derive an approximation of the transmission rate, which will be useful for the subsequent asymptotic analysis. Our focus is on systems employing MRC-GSIC and ZF-GSIC receivers. For the approximations related to ZF-GSIC systems in the asymptotic analysis, we refer to^27^.

#### MRC-GSIC receivers with perfect CSI

Next, we derive asymptotic approximations of the SINR for MRC and ZF receivers under perfect CSI. This analysis provides insights into the effects of the $\:\mathcal{K}$-factor, ΣΔ-ADC resolution, and the number of BS antennas on SE. As discussed in Sect.  1, the estimated Rayleigh fading channel vectors for different $\:[\mathbf{G}{]}_{m}$are mutually independent, and their entries can be modeled as i.i.d. random variables (RVs). For a given UE $\:k$, the entries of $\:{\:\left[\mathbf{G}\right]}_{\mathrm{m}\mathrm{k}}\approx\:CN(0,\:{\beta\:}_{k})$can be expressed as:

75 $$\:{\left[\mathbf{G}\right]}_{\mathrm{m}\mathrm{k}}=\sqrt{\frac{{\mathcal{K}}_{k}{{\upbeta\:}}_{\mathrm{k}}}{{\mathcal{K}}_{k}+\:1}}\:{{\upsigma\:}}_{\mathrm{m}\mathrm{k}\:}+\sqrt{\frac{1}{{\mathcal{K}}_{k}+\:1}}{\mathrm{q}}_{\mathrm{m}\mathrm{k}}$$

where $\:{\:{{\upsigma\:}}_{\mathrm{m}\mathrm{k}\:}=\mathrm{e}}^{-\mathrm{j}(\mathrm{m}-1){\uppi\:}\:\mathrm{s}\mathrm{i}\mathrm{n}\:\left({\Theta}_{\mathrm{k}}\right)}={{\upsigma\:}}_{\mathrm{m}\mathrm{k}}^{C}-J{{\upsigma\:}}_{\mathrm{m}\mathrm{k}}^{S}$and $\:{\mathrm{q}}_{\mathrm{m}\mathrm{k}}={\mathrm{q}}_{\mathrm{m}\mathrm{k}}^{c}+J{\mathrm{q}}_{\mathrm{m}\mathrm{k}}^{s}$ with zero mean and variance of $\:\frac{1}{2}$ for independent real and imaginary parts $\:{\mathrm{q}}_{\mathrm{m}\mathrm{k}}^{\mathrm{c}}$ and $\:{\mathrm{q}}_{\mathrm{m}\mathrm{k}}^{\mathrm{s}}$. In the following, we consider large-system limits to derive asymptotic approximations of the SE. $\:{\Vert\:\:{\mathbf{g}}_{\mathrm{k}}\:\Vert\:}^{2}\:$can be written as:

76 $$\:{\Vert\:{\mathbf{g}}_{\mathrm{k}}\Vert\:}^{2}=\sqrt{\frac{{\mathrm{K}}_{\mathrm{k}}{{\upbeta\:}}_{\mathrm{k}}}{{\mathrm{K}}_{\mathrm{k}}+\:1}}{{\upsigma\:}}_{\mathrm{m}\mathrm{n}}+\sqrt{\frac{1}{{\mathrm{K}}_{\mathrm{k}}+\:1}}\:{\mathrm{q}}_{\mathrm{m}}\left)\right(\sqrt{\frac{{\mathrm{K}}_{\mathrm{k}}{{\upbeta\:}}_{\mathrm{k}}}{{\mathrm{K}}_{\mathrm{k}}+\:1}}{{\upsigma\:}}_{\mathrm{m}\mathrm{k}}{+\sqrt{\frac{1}{{\mathrm{K}}_{\mathrm{k}}+\:1}}\:{\mathrm{q}}_{\mathrm{m}\mathrm{k}})}^{\mathrm{H}}\:\:$$

Equation (76) can be expanded to:

$$\:{\Vert\:{\mathbf{g}}_{\mathrm{k}}\:\Vert\:}^{2}=\frac{1}{\sqrt{({\mathrm{K}}_{\mathrm{k}}+\:1){(\mathrm{K}}_{\mathrm{k}}+\:1)}}{\sum\:}_{\mathrm{m}=1}^{\mathrm{M}}\mathbb{E}\{{\mathrm{K}}_{\mathrm{k}}{{{{\upbeta\:}}_{\mathrm{k}}\:{\upsigma\:}}_{\mathrm{m}\mathrm{k}}}^{\mathrm{*}}{{\upsigma\:}}_{\mathrm{m}\mathrm{n}}+\sqrt{{\mathrm{K}}_{\mathrm{n}}}{{{\upsigma\:}}_{\mathrm{m}\mathrm{n}}}^{\mathrm{*}}{\mathrm{q}}_{\mathrm{m}\mathrm{n}}+\sqrt{{\mathrm{K}}_{\mathrm{n}}}{{{\upbeta\:}}_{\mathrm{k}}{\upsigma\:}}_{\mathrm{m}\mathrm{n}}{{\mathrm{q}}_{\mathrm{m}\mathrm{n}}}^{\mathrm{*}}+\:{{\mathrm{q}}_{\mathrm{m}\mathrm{n}}}^{\mathrm{*}}{\mathrm{q}}_{\mathrm{m}\mathrm{n}}\:\}$$

since$\:\:{\sigma\:}_{mn}\:$is uncorrelated with $\:{q}_{mn}$.

which yields:

77 $$\:{\Vert\:{\mathbf{g}}_{\mathrm{k}}\:\Vert\:}^{2}=\frac{1}{{\mathrm{K}}_{\mathrm{k}}+\:1}{\sum\:}_{\mathrm{m}=1}^{\mathrm{M}}{(\mathrm{K}}_{\mathrm{n}}\:{{\upbeta\:}}_{\mathrm{k}}\:+{{\upbeta\:}}_{\mathrm{k}})=\frac{{\mathrm{M}{{\upbeta\:}}_{\mathrm{k}}\:(\mathrm{K}}_{\mathrm{K}}+\:1)}{{\mathrm{K}}_{\mathrm{K}}+\:1}\:$$

$\:{\Vert\:{\mathbf{g}}_{\mathrm{k}}\:\Vert\:}^{4}\:$can be written as:

78 $$\:{\Vert\:{\mathbf{g}}_{\mathrm{k}}\:\Vert\:}^{4}=\frac{{\mathrm{M}{{\upbeta\:}}_{\mathrm{k}}}^{2}(2{\mathrm{K}}_{\mathrm{K}}+2\mathrm{M}{\mathrm{K}}_{\mathrm{K}}+\mathrm{M}{{\mathrm{K}}_{\mathrm{K}}}^{2}+\mathrm{M}+1}{{(\mathrm{K}}_{\mathrm{K}}\:{+\:1)}^{2}}\:$$

and the cross-term with another user $\:i$ is $\:{\Vert\:{\mathbf{g}}_{\mathrm{k}}\:{\mathbf{g}}_{\mathrm{i}}\:\Vert\:}^{2}$ can be written as:

79 $$\:{\Vert\:{\mathbf{g}}_{\mathrm{k}}\:{\mathbf{g}}_{\mathrm{i}}\:\Vert\:}^{2}=\frac{{{\upbeta\:}}_{\mathrm{k}}{{\upbeta\:}}_{\mathrm{I}}{{{(\mathrm{K}}_{\mathrm{k}}{\mathrm{K}}_{\mathrm{i}}{\Phi\:}}_{\mathrm{k}\mathrm{i}}}^{2}+3\mathrm{M})}{{{(\mathrm{K}}_{\mathrm{k}}+\:1\left)\right(\mathrm{K}}_{\mathrm{k}}+\:1)}\:$$

Where $\:{\varPhi\:}_{ki}\:$is defined as:

80 $$\:{{\Phi\:}}_{\mathrm{k}\mathrm{i}}=\frac{\mathrm{sin}\left(\frac{\mathrm{M}{\uppi\:}}{2}\left(\mathrm{s}\mathrm{i}\mathrm{n}\right({{\uptheta\:}}_{\mathrm{K}})-\mathrm{s}\mathrm{i}\mathrm{n}({{\uptheta\:}}_{\mathrm{i}})\right)}{\mathrm{s}\mathrm{i}\mathrm{n}\left(\frac{{\uppi\:}}{2}\left(\mathrm{s}\mathrm{i}\mathrm{n}\right({{\uptheta\:}}_{\mathrm{K}})-\mathrm{s}\mathrm{i}\mathrm{n}({{\uptheta\:}}_{\mathrm{i}})\right)}\:$$

The interference-plus-noise power for perfect ($\:{\mathrm{T}}_{\mathrm{k}}^{\mathrm{p}\mathrm{e}\mathrm{r}}$) channel estimation can be approximated as:

81 $$\:{\mathrm{T}}_{\mathrm{k}}^{\mathrm{p}\mathrm{e}\mathrm{r}}=\mathrm{M}{{\upbeta\:}}_{\mathrm{k}}[{a}_{m{\prime\:}}^{2}\:{{\upsigma\:}}^{2}+{a}_{\mathrm{m}{\prime\:}}(1-{a}_{\mathrm{m}{\prime\:}}\left)\right({\mathrm{P}}_{\mathrm{s}}{\sum\:}_{\begin{array}{c}i=\left({g}_{n}-1\right)G+1\:\\\:,i\ne\:k\end{array}}^{\mathrm{K}}{{\upbeta\:}}_{\mathrm{k}}+{{\upsigma\:}}^{2}]{+{a}_{m{\prime\:}}^{2}\mathrm{P}}_{\mathrm{s}}\frac{{{{\upbeta\:}}_{\mathrm{i}}{\upbeta\:}}_{\mathrm{k}}({{{\mathrm{K}}_{\mathrm{k}}{\mathrm{K}}_{\mathrm{i}}{\varnothing}}_{\mathrm{k}\mathrm{i}}}^{2}+3\mathrm{M})}{{{(\mathrm{K}}_{\mathrm{k}}+\:1\left)\right(\mathrm{K}}_{\mathrm{k}}+\:1)}$$

82 $$\:{\mathrm{T}}_{\mathrm{k}}^{\mathrm{p}\mathrm{e}\mathrm{r}}=\mathrm{M}{{\upbeta\:}}_{\mathrm{k}}[{a}_{\mathrm{m}{\prime\:}}\:{{\upsigma\:}}^{2}+\:{a}_{\mathrm{m}{\prime\:}}\:(1\:-\:{a}_{\mathrm{m}{\prime\:}}\left)\right({\mathrm{P}}_{\mathrm{s}}{\sum\:}_{\begin{array}{c}i=\left({g}_{n}-1\right)G+1\:\\\:,i\ne\:k\end{array}}^{\mathrm{K}}{{\upbeta\:}}_{\mathrm{k}}\left)\right]+{a}_{m{\prime\:}}^{2}\:{\mathrm{P}}_{\mathrm{s}}\frac{{{{\upbeta\:}}_{\mathrm{i}}{\upbeta\:}}_{\mathrm{k}}({(\mathrm{K}}_{\mathrm{k}}{\mathrm{K}}_{\mathrm{i}}{{{\Phi\:}}_{\mathrm{k}\mathrm{i}}}^{2}+3\mathrm{M})}{{{(\mathrm{K}}_{\mathrm{k}}+\:1\left)\right(\mathrm{K}}_{\mathrm{k}}+\:1)}\:$$

Finally, substituting the approximations into the SINR expression for MRC-GSIC receivers with perfect CSI gives

83 $$\:{{\upgamma\:}}_{\mathrm{k}}\approx\:\frac{{a}_{m{\prime\:}}^{2}{\mathrm{P}}_{\mathrm{s}}\frac{\mathrm{M}{{\upbeta\:}}_{\mathrm{k}}}{{(\mathrm{K}}_{\mathrm{k}}\:{+\:1)}^{2}}(2{\mathrm{K}}_{\mathrm{k}}+2{\mathrm{M}\mathrm{K}}_{\mathrm{k}}+\mathrm{M}{\mathrm{K}}_{\mathrm{k}}^{2}+\mathrm{M}+1)}{{\mathrm{T}}_{\mathrm{k}}^{\mathrm{p}\mathrm{e}\mathrm{f}}}\:$$

#### ZF-GSIC receivers with perfect CSI

From Eq. (39), the distortion-plus-noise power UE *k* consists of two components: the channel noise power $\:{a}_{m{\prime\:}}^{2}\:{{\Vert\:\mathrm{g}}_{\mathrm{k}}\Vert\:}^{2}\:{{\upsigma\:}}^{2}$ and the ADC quantization noise power$\:\:{\mathbf{g}}_{\mathrm{k}}^{\mathrm{H}}{{\:\mathbf{R}}_{\mathbf{v}}}_{\mathrm{q}}{\:\mathbf{g}}_{\mathbf{k}}\:$where $\:{\parallel\:\:{\mathbf{g}}_{\mathrm{k}}\:\:\parallel\:}^{2}={\left[{\left({\mathbf{G}}^{\mathrm{H}}\:\mathbf{G}\:\right)}^{-1}\right]}_{\mathrm{k},\mathrm{k}}\:=\mathbb{E}{\left[{\left({\mathbf{G}}^{\mathrm{H}}\:\mathbf{G}\right)}^{-1}\right]}_{\mathrm{k},\mathrm{k}}$, the expectation is taken with respect to the small-scale fading. From [15, Theorem 4], $\:{\mathbf{G}}^{\mathrm{H}}\:\mathbf{G}$ follows a non-central Wishart distribution, and the squared Euclidean norm of UE *k*’s filter satisfies

84 $$\:{\parallel\:\:{\mathbf{g}}_{\mathrm{k}}\:\:\parallel\:}^{2}=\frac{{\left[{\sum\:}^{-1}\right]}_{\mathrm{k},\mathrm{k}}}{{{\upbeta\:}}_{\mathrm{k}}\:(\mathrm{M}-\mathrm{K}\:+\:(\left({\mathrm{v}}_{\mathrm{k}}-1\right)\mathrm{G})}\:$$

where

85 $$\:\sum\:={\:(\boldsymbol{\Theta\:}+\:{\mathbf{I}}_{\mathrm{k}})}^{-1}+\frac{1}{\mathrm{M}}{\:\left[\boldsymbol{\Theta\:}\:\right(\boldsymbol{\Theta\:}+\:{\mathbf{I}}_{\mathrm{k}})}^{-1}{]}^{1/2}{\mathbf{G}}^{\mathrm{H}}\:\mathbf{G}\:\:{\left[\right(\boldsymbol{\Theta\:}\:+{\mathbf{I}}_{\mathrm{k}})}^{-1}{]}^{1/2}\:$$

Finally, substituting the expressions for channel noise and ADC quantization noise, the distortion-plus-noise power for the ZF receiver can be approximated, and the corresponding SINR for UE *k* is given by:

86 $$\:{{\upgamma\:}}_{\mathrm{k}}=\frac{{{\mathrm{P}}_{\mathrm{s}}\:{\upbeta\:}}_{\mathrm{k}}\:(\mathrm{M}-\mathrm{K}\:+\:(\left({g}_{n}-1\right)\:\mathrm{G})}{(\:\frac{{{\upsigma\:}}^{2}}{{a}_{\mathrm{m}{\prime\:}}}+(\:\frac{1}{{a}_{\mathrm{m}{\prime\:}}}-1\left)\:{\mathrm{P}}_{\mathrm{s}}\sum\:_{\mathrm{k}=1}^{\mathrm{K}}{\:{\upbeta\:}}_{\mathrm{i}}\right){\left[{\sum\:}^{-1}\right]}_{\mathrm{k},\mathrm{k}}\:}\:$$

#### MRC-GSIC receivers with imperfect CSI

Next, we derive asymptotic approximations of the SINR for MRC and ZF receivers under imperfect CSI. This provides insights into the effects of the $\:\mathcal{K}$-factor, ADC resolution, and the number of BS antennas on SE. As discussed in Sect.  1, the estimated Rayleigh fading channel vectors $\:[\mathbf{G}{]}_{w}$are mutually independent, and their entries can be modeled as i.i.d. RVs. For a given UE $\:k$, the entries of $\:[\mathbf{G}{]}_{w,k}$,$\:{\:\left[\mathbf{G}\right]}_{\mathrm{m}\mathrm{k}}\approx\:\mathrm{C}\mathrm{N}(0,\:{{\upbeta\:}}_{\mathrm{k}}{{\upeta\:}}_{\mathrm{k}\:})$ can be expressed as:

87 $$\:{\left[\mathbf{G}\right]}_{\mathrm{m}\mathrm{k}}=\sqrt{\frac{{\mathcal{K}}_{\mathrm{k}}{{\upbeta\:}}_{\mathrm{k}}}{{\mathcal{K}}_{\mathrm{k}}+\:1}}\:{{\upsigma\:}}_{\mathrm{m}\mathrm{k}\:}+\sqrt{\frac{1}{{\mathcal{K}}_{\mathrm{k}}+\:1}}{\mathrm{q}}_{\mathrm{m}\mathrm{k}}\:\:\:$$

where$\:{\:{{\upsigma\:}}_{\mathrm{m}\mathrm{k}\:}=\mathrm{e}}^{-\mathrm{j}(\mathrm{m}-1){\uppi\:}\:\mathrm{s}\mathrm{i}\mathrm{n}\:\left({\Theta}_{\mathrm{k}}\right)}={{\upsigma\:}}_{\mathrm{m}\mathrm{k}}^{C}-J{{\upsigma\:}}_{\mathrm{m}\mathrm{k}}^{S}$ and $\:{\mathrm{q}}_{\mathrm{m}\mathrm{k}}={\mathrm{q}}_{\mathrm{m}\mathrm{k}}^{c}+J{\mathrm{q}}_{\mathrm{m}\mathrm{k}}^{s}$with zero mean and variance of $\:\frac{{\beta\:}_{k}{\eta\:}_{k\:}}{2}\:$for independent real and imaginary parts $\:{\mathrm{q}}_{\mathrm{m}\mathrm{k}}^{\mathrm{c}}$ and $\:{q}_{mk}^{s}$. In the following, we assume large systems and derive asymptotic approximations of the SE. Moreover, $\:{\Vert\:\:{\mathbf{g}}_{\mathrm{k}\:}\Vert\:}^{2}\:$can be written as:

88 $$\:{\Vert\:{\widehat{\mathbf{g}}}_{\mathrm{k}}\Vert\:}^{2}=\frac{1}{{\mathrm{K}}_{\mathrm{k}}+\:1}\:{\sum\:}_{\mathrm{m}=1}^{\mathrm{M}}{(\mathcal{K}}_{\mathrm{k}}{{\upbeta\:}}_{\mathrm{k}}+{{\upeta\:}}_{\mathrm{k}\:}{{\upbeta\:}}_{\mathrm{k}})=\frac{{\mathrm{M}{{\upbeta\:}}_{\mathrm{k}}(\mathcal{K}}_{\mathrm{k}}+{{\upeta\:}}_{\mathrm{k}\:})}{{\mathcal{K}}_{\mathrm{k}}+\:1}$$

and $\:{\Vert\:{\widehat{\mathbf{g}}}_{\mathrm{k}}\:\Vert\:}^{4}\:$can be written as

89 $$\:{\Vert\:\:{\widehat{\mathbf{g}}}_{\mathrm{k}}\Vert\:}^{4}=\frac{\mathrm{M}{{\upbeta\:}}_{\mathrm{k}}^{2}(\mathrm{M}+1){{\upeta\:}}_{\mathrm{k}}^{2}+2\mathrm{M}{\mathrm{K}}_{\mathrm{k}}{{\upeta\:}}_{\mathrm{k}\:}+\mathrm{M}{\mathcal{K}}_{\mathrm{k}}^{2}+2{\mathcal{K}}_{\mathrm{k}}{{\upeta\:}}_{\mathrm{k}\:}}{{(\mathcal{K}}_{\mathrm{k}}\:{+\:1)}^{2}}\:$$

Then, the cross-term with another user $\:i$ is $\:{\Vert\:{\widehat{\mathbf{g}}}_{\mathrm{k}}\:{\widehat{\mathbf{g}}}_{\mathrm{i}}\:\Vert\:}^{2}$ can be presented as:

90 $$\:{\Vert\:{\widehat{\:\mathbf{g}}}_{\mathrm{k}}{\widehat{\mathbf{g}}}_{\mathrm{i}}\:\Vert\:}^{2}=\frac{{{{{{\upbeta\:}}_{\mathrm{i}}{\upbeta\:}}_{\mathrm{k}}(\mathcal{K}}_{\mathrm{k}}\mathcal{K}}_{\mathrm{i}}{{\varnothing}}_{\mathrm{k}\mathrm{i}}^{2}+{\mathrm{M}\mathcal{K}}_{\mathrm{k}}{{\upeta\:}}_{\mathrm{i}}+\mathrm{M}{\mathcal{K}}_{\mathrm{i}}{{\upeta\:}}_{\mathrm{k}\:}+\mathrm{M}{{\upxi\:}}_{\:\mathrm{i}}{{\upeta\:}}_{\mathrm{k}\:})}{{{(\mathcal{K}}_{\mathrm{k}}+\:1\left)\right(\mathcal{K}}_{\mathrm{i}}+\:1)}\:$$

For the ADC quantization noise in Eq. (61), the following approximation is accurate for large-scale MIMO systems:

91 $$\:{\widehat{\mathbf{g}}}_{\mathrm{k}}^{\mathrm{H}}{{\mathbf{R}}_{\mathbf{v}}}_{\mathrm{q}}{\widehat{\mathbf{g}\:}}_{\mathrm{k}}={a}_{\mathrm{m}{\prime\:}}(1-{a}_{\mathrm{m}{\prime\:}})\:({\mathrm{P}}_{\mathrm{s}}{\sum\:}_{\mathrm{k}=1}^{\mathrm{K}}{{\upbeta\:}}_{\mathrm{k}}+{{\upsigma\:}}^{2})\frac{{\mathrm{M}{{\upbeta\:}}_{\mathrm{k}}(\mathcal{K}}_{\mathrm{k}}+{{\upeta\:}}_{\mathrm{k}})\:\:\:\:\:\:\:\:}{{\mathcal{K}}_{\mathrm{k}}+\:1}$$

Substituting the above approximations into Eq. (82), the interference-plus-noise power $\:{\mathrm{T}}_{\mathrm{k}}^{\mathrm{i}\mathrm{m}\mathrm{p}\mathrm{e}\mathrm{r}}$ can be approximated as.

  

92 $$\begin{aligned}{\mathrm{T}}_{\mathrm{k}}^{\mathrm{i}\mathrm{m}\mathrm{p}\mathrm{e}\mathrm{r}}&=\frac{{\mathrm{M}{{\upbeta\:}}_{\mathrm{k}}(\mathcal{K}}_{\mathrm{k}}+{{\upeta\:}}_{\mathrm{k}\:})}{{\mathcal{K}}_{\mathrm{k}}+\:1}[{a}_{m{\prime\:}}^{2}\:+\:{a}_{\mathrm{m}{\prime\:}}(1\:-\:{a}_{\mathrm{m}{\prime\:}}\left){\mathrm{P}}_{\mathrm{s}}\right({\sum\:}_{k=1}^{K}{\beta\:}_{k}+\:{{\upsigma\:}}^{2}\:)\nonumber\\&+{\mathrm{P}}_{\mathrm{s}}\:\:{\sum\:}_{\mathrm{i}=1}^{K}{{{\upsigma\:}}^{2}}_{\mathrm{e}\mathrm{i}}]\:\:+{\mathrm{P}}_{\mathrm{s}}\:{a}_{m{\prime\:}}^{2}{\sum\:}_{\begin{array}{c}i=\left({g}_{n}-1\right)G+1\:\\\:,i\ne\:k\end{array}}^{K}\frac{{{{{{\upbeta\:}}_{\mathrm{i}}{\upbeta\:}}_{\mathrm{k}}(\mathcal{K}}_{\mathrm{k}}\mathcal{K}}_{\mathrm{i}}{{\Phi\:}}_{\mathrm{k}\mathrm{i}}+{\mathrm{M}\mathcal{K}}_{\mathrm{k}}{{\upeta\:}}_{\mathrm{i}\:}+\mathrm{M}{\mathcal{K}}_{\mathrm{i}}{{\upeta\:}}_{\mathrm{k}\:}+\mathrm{M}{{\upeta\:}}_{\mathrm{i}\:}{{\upeta\:}}_{\mathrm{k}\:})}{{{(\mathcal{K}}_{\mathrm{k}}+\:1\left)\right(\mathcal{K}}_{\mathrm{i}}+\:1)}\end{aligned}$$

Then, the asymptotic SINR for MRC-GSIC receivers with imperfect CSI can be approximated as:

93 $$\:{\widehat{{\upgamma\:}}}_{\mathrm{k}}=\frac{{\:\mathrm{a}}^{2}{\mathrm{P}}_{\mathrm{s}}\frac{{\mathrm{M}{\upbeta\:}}_{\mathrm{k}}^{2}}{{({\mathcal{K}}_{\mathrm{k}}+1)}^{2}}(2{\mathcal{K}}_{\mathrm{k}}{{\upeta\:}}_{\mathrm{k}\:}+2{\mathrm{M}\mathcal{K}}_{\mathrm{k}}{{\upeta\:}}_{\mathrm{k}\:}+\mathrm{M}{\mathcal{K}}_{\mathrm{k}}^{2}+\left(\mathrm{M}+1\right){{\upeta\:}}_{\mathrm{k}}^{2})}{{\mathrm{T}}_{\mathrm{k}}^{\mathrm{i}\mathrm{m}\mathrm{p}\mathrm{e}\mathrm{r}}\:\:}\:$$

#### ZF-GSIC receivers with imperfect CSI

From Eq. (71), the distortion-plus-noise power for ZF-GSIC receivers with imperfect CSI consists of three components: the channel noise power $\:{a}_{m{\prime\:}}^{2}{{\Vert\:\mathbf{g}}_{\mathrm{k}}\Vert\:}^{2}\:{{\upsigma\:}}^{2}$ and the ADC quantization noise power $\:\:{\widehat{\mathbf{g}}}_{\mathrm{k}}^{\mathrm{H}}{{\:\mathbf{R}}_{\mathbf{v}}}_{\mathrm{q}}{\:\widehat{\mathbf{g}}}_{\mathrm{k}}\:$and the final part IUI $\:\:{{a}_{m{\prime\:}}^{2}\parallel\:{\widehat{\mathbf{g}}}_{\mathrm{k}}\parallel\:}^{2}{\sum\:}_{\mathrm{i}=1}^{\mathrm{K}}{{{\upsigma\:}}^{2}}_{\mathrm{e}\mathrm{i}}\:{\mathrm{P}}_{\mathrm{i}}$ where

94 $$\:{\parallel\:\:{\widehat{\mathbf{g}}}_{\mathbf{k}}\:\:\parallel\:}^{2}={\left[{\left({\widehat{\mathbf{G}}}^{\mathrm{H}}\:\widehat{\mathbf{G}}\right)}^{-1}\right]}_{\mathrm{k},\mathrm{k}}\:=\mathbb{E}\:{\left[{\left({\widehat{\mathbf{G}}}^{\mathrm{H}}\:\widehat{\mathbf{G}}\right)}^{-1}\right]}_{\mathrm{k},\mathrm{k}}\:$$

where the expectation is taken with respect to the small-scale fading. According to [^15^, Theorem 4], $\:{\mathbf{G}}^{\mathrm{H}}\:\mathbf{G}$ follows a non-central Wishart distribution and the squared Euclidean norm of UE *k*’s filter can be approximated as

95 $$\:{\parallel\:\:{\:\widehat{\mathbf{g}\:}}_{\mathrm{k}}\:\:\parallel\:}^{2}=\frac{{\left[{\sum\:}^{-1}\right]}_{\mathrm{k},\mathrm{k}}}{{{\upbeta\:}}_{\mathrm{k}}\:(\mathrm{M}-\mathrm{K}\:+\:(\left({\mathrm{g}}_{\mathrm{n}}-1\right)\mathrm{G})}\:$$

Where

96 $$\:\widehat{\sum\:}={\:\mathbb{E}(\boldsymbol{\Theta\:}+\:{\mathbf{I}}_{\mathrm{k}})}^{-1}+\frac{1}{\mathrm{M}}{\:\left[\boldsymbol{\Theta\:}\:\right(\boldsymbol{\Theta\:}+\:{\mathrm{I}}_{\mathrm{k}})}^{-1}{]}^{1/2}{\widehat{\mathbf{G}}}^{\mathrm{H}}\:\widehat{\mathbf{G}}\:\:{\left[\right(\boldsymbol{\Theta\:}\:+{\mathbf{I}}_{\mathrm{k}})}^{-1}{]}^{1/2}\:$$

and $\:\mathbb{E}\:\:\mathrm{i}\mathrm{s}\:\mathrm{a}\:K\:\times\:K\:$diagonal matrix with$\:{\left[\mathbb{E}\right]}_{k,k\:}={{\upeta\:}}_{\mathrm{k}\:}$where $\:{{\upeta\:}}_{\mathrm{k}\:}$is defined in Eq. (9). The corresponding asymptotic SINR for UE *k* can be approximated as:

97 $$\:{{\upgamma\:}}_{\mathrm{k}}=\frac{{{\upbeta\:}}_{\mathrm{k}}\:(\mathrm{M}-\mathrm{K}\:+\:\left({g}_{n}-1\right)\:\mathrm{G})}{({a}_{\mathrm{m}{\prime\:}}{{\upsigma\:}}^{2}+{a}_{\mathrm{m}{\prime\:}}\:(1-{a}_{\mathrm{m}{\prime\:}}\:)\:{\mathrm{P}}_{\mathrm{s}}\sum\:_{\mathrm{n}=1}^{\mathrm{K}}{\:{\upbeta\:}}_{\mathrm{n}}+\mathrm{a}\:{\mathrm{P}}_{\mathrm{s}}\sum\:_{\mathrm{i}=1}^{\mathrm{K}}{{{\upsigma\:}}^{2}}_{{\mathrm{e}}_{\mathrm{i}}}){\left[{\sum\:}^{-1}\right]}_{\mathrm{k},\mathrm{k}}}$$

### Asymptotic analysis

In this section, we derive the power scaling laws for MRC-GSIC and ZF-GSIC receivers under imperfect CSI to gain insight into the performance when the number of antennas increases.

#### MRC-GSIC receivers

Assume that the number of antennas is very large, i.e.$\:M\:\to\:\infty\:.\:$ Multiply $\:\frac{1}{{M}^{2}}$to the numerator and denominator of denominator of Eq. (23) and let $\:{P}_{s}=\frac{{E}_{s}}{{M}^{v}}$ where $\:\nu\:>0$ and $\:{E}_{s}$is a fixed value. We have

98 $$\:{a}_{m{\prime\:}}^{2}\frac{{\mathrm{E}}_{\mathrm{s}}}{{\mathrm{M}}^{\mathrm{v}+2}}{\Vert\:{\:\widehat{\mathbf{g}\:}}_{\mathrm{k}}{\:\widehat{\mathbf{g}\:}}_{\mathrm{i}}\Vert\:}^{2}={a}_{m{\prime\:}}^{2}\:\frac{{\mathrm{E}}_{\mathrm{s}}}{{\mathrm{M}}^{\mathrm{v}}}{\Vert\:\frac{1}{\mathrm{M}}{\:\widehat{\mathbf{g}}}_{\mathrm{k}}{\:\widehat{\mathbf{g}}}_{\mathrm{i}}\Vert\:}^{2}\:\underrightarrow{\mathrm{a}.\mathrm{s}.}0\:$$

Following Eq. (59), removing the relevant zero items in Eq. (43), it then reduces to

99 $$\:{\widehat{\mathrm{I}}}_{\mathrm{k}}\:=\:{a}_{{\mathrm{m}}^{{\prime\:}}}\Vert\:{{\:\widehat{\mathbf{g}}}_{\mathrm{k}}\Vert\:}^{2}\:{{\upsigma\:}}^{2}\:\:+\:{a}_{{\mathrm{m}}^{{\prime\:}}}\left(1-{a}_{{\mathrm{m}}^{{\prime\:}}}\right){\:\mathrm{P}}_{\mathrm{s}}\:{\sum\:}_{\mathrm{i}=1}^{\mathrm{K}}{\:\widehat{\mathrm{Q}}}_{\mathrm{k}\mathrm{i}}+{a}_{{m}^{{\prime\:}}}^{2}\:{\mathrm{p}}_{\mathrm{s}}{\sum\:}_{\begin{array}{c}i=\left({g}_{n}-1\right)G+1\:\\\:,i\ne\:n\end{array}}^{\mathrm{K}}{\mid\:{\widehat{\mathbf{g}}}_{\mathrm{k}}^{\mathrm{H}}{\:\widehat{\mathbf{g}\:}}_{\mathrm{i}}|}^{2}+{a}_{{m}^{{\prime\:}}}^{2}\Vert\:{{\:\widehat{\mathbf{g}}}_{\mathrm{k}}\Vert\:}^{2}{\mathrm{P}}_{\mathrm{s}}{\sum\:}_{\mathrm{i}=1}^{\mathrm{K}}{\sigma\:}^{2}\:{e}_{i}\:$$

100 $$\:{\widehat{\mathrm{I}}}_{\mathrm{k}}={a}_{{\mathrm{m}}^{{\prime\:}}}\Vert\:{\:\widehat{\mathbf{g}}}_{\mathrm{k}}{\Vert\:{\upsigma\:}}^{2}+{a}_{{\mathrm{m}}^{{\prime\:}}}\left(1-{a}_{{\mathrm{m}}^{{\prime\:}}}\right)\frac{{\mathrm{E}}_{\mathrm{u}}}{{\mathrm{M}}^{\mathrm{v}}}{\sum\:}_{\mathrm{i}=1}^{\mathrm{K}}{\:\widehat{\mathrm{Q}}}_{\mathrm{K}\mathrm{i}}+\:{a}_{{m}^{{\prime\:}}}^{2}\frac{{\mathrm{E}}_{\mathrm{s}}}{{\mathrm{M}}^{\mathrm{v}}}{\sum\:}_{\begin{array}{c}i=\left({\mathrm{g}}_{\mathrm{k}}-1\right)G+1\:\\\:,i\ne\:n\end{array}}^{\mathrm{K}}{\mid\:{\widehat{\mathbf{g}}}_{\mathrm{k}}^{\mathrm{H}}{\:\widehat{\mathbf{g}\:}}_{\mathrm{i}}|}^{2}+{a}_{m{\prime\:}}^{2}{\Vert\:{\:\widehat{\mathbf{g}\:}}_{\mathrm{k}}\Vert\:}^{2}\frac{{\mathrm{E}}_{\mathrm{s}}}{{\mathrm{M}}^{\mathrm{v}}}{\sum\:}_{\mathrm{i}=1}^{\mathrm{K}}{\sigma\:}^{2}\:{e}_{i}$$

101 $$\:{\widehat{\mathrm{I}}}_{\mathrm{k}}\:\underrightarrow{M\to\:{\infty\:}}\:{a}_{\mathrm{m}{\prime\:}}\:\Vert\:{{\widehat{\mathbf{g}}}_{\mathrm{k}}\Vert\:}^{2}\:{{\upsigma\:}}^{2}$$

Substituting the approximations into Eq. (24), we can approximate $\:{\widehat{{\upgamma\:}}}_{\mathrm{k}}\:$as follows:

102 $$\:{\widehat{{\upgamma\:}}}_{\mathrm{k}}=\frac{{a}_{m{\prime\:}}^{2}\frac{{\mathrm{E}}_{\mathrm{s}}}{{\mathrm{M}}^{\mathrm{v}}}{\Vert\:{\:\widehat{\mathbf{g}\:}}_{\mathrm{k}}\Vert\:}^{4}}{{a}_{\mathrm{m}{\prime\:}}\:\Vert\:{{\:\widehat{\mathbf{g}\:}}_{\mathrm{k}}\Vert\:}^{2}\:{{\upsigma\:}}^{2}\:\:}=\frac{{a}_{m{\prime\:}}^{2}\frac{{\mathrm{E}}_{\mathrm{s}}}{{\mathrm{M}}^{\mathrm{v}}}{\Vert\:{\widehat{\mathbf{g}}}_{\mathrm{k}}\Vert\:}^{2}}{\mathrm{a}\:{{\upsigma\:}}^{2}}$$

103 $$\:{\widehat{{\upgamma\:}}}_{\mathrm{k}}=\frac{{a}_{\mathrm{m}{\prime\:}}\:{\mathrm{E}}_{\mathrm{s}}{\Vert\:\frac{1}{\mathrm{M}}{\widehat{\mathbf{g}}}_{\mathrm{k}}\Vert\:}^{2}}{{{{\upsigma\:}}^{2}\mathrm{M}}^{\mathrm{v}-1\:}}\:$$

when M is large, $\:\Vert\:{{\widehat{\mathbf{g}}}_{\mathrm{k}}\Vert\:}^{2}$ can be approximated by an identity matrix. Following this, substituting Eq. (55) into Eq. (65) leads to:

104 $$\:{\widehat{{\upgamma\:}}}_{\mathrm{k}}\underrightarrow{M\to\:{\infty\:}}\:\frac{{a}_{\mathrm{m}{\prime\:}}\:{\mathrm{E}}_{\mathrm{s}}}{{{{\upsigma\:}}^{2}\mathrm{M}}^{\mathrm{v}-1\:}}\:\left\{\frac{{{{\upbeta\:}}_{\mathrm{k}}\mathcal{K}}_{\mathrm{k}}}{{\mathcal{K}}_{\mathrm{k}}+\:1}\:+\frac{{{{\upbeta\:}}_{\mathrm{k}}{\upeta\:}}_{\mathrm{k}\:}}{{\mathcal{K}}_{\mathrm{k}}+\:1}\right\},\approx\:\frac{{{a}_{\mathrm{m}{\prime\:}}\:{\mathrm{E}}_{\mathrm{s}}{\upbeta\:}}_{\mathrm{k}}{\mathcal{K}}_{\mathrm{k}}}{{\mathrm{M}}^{\mathrm{v}-1\:}{{\upsigma\:}}^{2}({\mathcal{K}}_{\mathrm{k}}+\:1)}+\frac{{{a}_{\mathrm{m}{\prime\:}}\:{\mathrm{E}}_{\mathrm{s}}\:{\upbeta\:}}_{\mathrm{k}}{{\upeta\:}}_{\mathrm{k}\:}}{{\mathrm{M}}^{\mathrm{v}-1\:}{{\upsigma\:}}^{2}({\mathcal{K}}_{\mathrm{k}}+\:1)}$$

Substituting Eq. (39) into Eq. (23), we can obtain an approximation of the SINR$\:{\widehat{\:{\upgamma\:}}}_{\mathrm{k}}\:$for the system of MRC-GSIC receivers with imperfect CSI is given by:

105 $$\:{\widehat{{\upgamma\:}}}_{\mathrm{k}}\approx\:\frac{{{a}_{\mathrm{m}{\prime\:}}\:{\mathrm{E}}_{\mathrm{s}}{\upbeta\:}}_{\mathrm{k}}{\mathcal{K}}_{\mathrm{k}}\:}{{{\:\mathrm{M}}^{\mathrm{v}-1\:}}^{\:}{{\upsigma\:}}^{2}({\mathcal{K}}_{\mathrm{k}}+\:1)}\:+\frac{{\mathrm{a}\:{\mathrm{E}}_{\mathrm{s}}{\upbeta\:}}_{\mathrm{k}}{{\upeta\:}}_{\mathrm{k}\:}}{{{{\upsigma\:}}^{2}\mathrm{M}}^{2\mathrm{v}-1\:}({\mathcal{K}}_{\mathrm{k}}+\:1)(\mathrm{L}\frac{{\mathrm{E}}_{\mathrm{s}}}{{\mathrm{M}}^{\mathrm{v}}}+\frac{{{\upsigma\:}}^{2}}{{a}_{\mathrm{m}{\prime\:}}}\:+(\frac{1}{{a}_{\mathrm{m}{\prime\:}}}-1)\frac{{\mathrm{E}}_{\mathrm{s}}}{{\mathrm{M}}^{\mathrm{v}}}{\sum\:}_{\mathrm{i}=1}^{\mathrm{K}}{{\upbeta\:}}_{\mathrm{i}})}$$

106 $$\:{\widehat{{\upgamma\:}}}_{\mathrm{k}}\underrightarrow{M\to\:{\infty\:}}\frac{{{a}_{\mathrm{m}{\prime\:}}{\mathrm{E}}_{\mathrm{s}}{\upbeta\:}}_{\mathrm{k}}{\mathcal{K}}_{\mathrm{k}}}{{\mathrm{M}}^{\mathrm{v}-1\:}{{\upsigma\:}}^{2}({\mathcal{K}}_{\mathrm{k}}+\:1)}+\frac{{a}_{\mathrm{m}{\prime\:}}{\mathrm{E}}_{\mathrm{s}}^{2}\:{{\upbeta\:}}_{\mathrm{k}}^{2}\mathrm{L}}{{\mathrm{M}}^{2\mathrm{v}-1\:}\frac{{{\upsigma\:}}^{4}}{{a}_{{\mathrm{m}}^{{\prime\:}}}}({\mathcal{K}}_{\mathrm{k}}+\:1)}\:$$

The power-scaling behavior for the MRC-GSIC receivers can now be discussed from Eq. (45). For Rayleigh fading, i.e. $\:{\mathcal{K}}_{k}=\:0$, when $\:\nu\:\:=\:0.5$ the SINR tends to be a constant value.

107 $$\:{\widehat{{\upgamma\:}}}_{\mathrm{k}}\underrightarrow{M\to\:{\infty\:}}\frac{{\mathrm{a}}^{2}{\:{\mathrm{E}}_{\mathrm{s}}}^{2}{{{\upbeta\:}}_{\mathrm{k}}}^{2}\mathrm{L}}{{{\upsigma\:}}^{4}}\:$$

For Rician fading, i.e. $\:{\mathcal{K}}_{n}\ne\:\:0$,when $\:\nu\:\:=\:1$the SINR tends to:

108 $$\:{\widehat{{\upgamma\:}}}_{\mathrm{k}}\underrightarrow{M\to\:{\infty\:}}\frac{{\mathrm{a}\:{\mathrm{E}}_{\mathrm{s}}{\upbeta\:}}_{\mathrm{k}}{\mathcal{K}}_{\mathrm{k}}}{{{\upsigma\:}}^{2}(\mathcal{K}+\:1)}\:$$

We next consider the LoS dominating the channel fading i.e. Very strong LoS paths exist in the transmission. Assuming $\:{K}_{i}\:=\:{K}_{k}$ and $\:{K}_{k}\to\:\infty\:\:$Eq. (43) tends to

109 $$\:{\widehat{{\upgamma\:}}}_{\mathrm{k}}\underrightarrow{M\to\:{\infty\:}}\frac{{\:\mathrm{P}}_{\mathrm{s}}\:{\mathrm{M}}^{2}{{\upbeta\:}}_{\mathrm{k}}}{\mathrm{M}(\frac{{{\upsigma\:}}^{2}}{{a}_{\mathrm{m}{\prime\:}}}+\frac{(1-{a}_{\mathrm{m}{\prime\:}})}{{a}_{\mathrm{m}{\prime\:}}}\:{\mathrm{P}}_{\mathrm{s}}\:{\sum\:}_{\mathrm{i}=1}^{\mathrm{K}}{{\upbeta\:}}_{\mathrm{i}})+{\mathrm{P}}_{\mathrm{s}}{\sum\:}_{\begin{array}{c}i=\left({g}_{n}-1\right)G+1\:\\\:,i\ne\:k\end{array}}^{\mathrm{K}}{{\upbeta\:}}_{\mathrm{i}}{{\Phi\:}}_{\mathrm{k}\mathrm{i}}^{2}}\:$$

where $\:{{\Phi\:}}_{ki}$ is defined in Eq. (80).

#### ZF-GSIC receivers

We next derive the power scaling law for the ZF receivers under imperfect CSI, following the same treatments as for the MRC receivers. Assuming M → ∞ and removing the zero items when M → ∞, (46) then becomes

110 $$\:{\widehat{\mathrm{I}}}_{\mathrm{k}}\:={a}_{\mathrm{m}{\prime\:}}\:\Vert\:{{\:\widehat{\mathbf{g}}}_{\mathrm{k}}\Vert\:}^{2}\:{{\upsigma\:}}^{2}\:+{a}_{\mathrm{m}{\prime\:}}(1-{a}_{\mathrm{m}{\prime\:}})\frac{{\mathrm{E}}_{\mathrm{u}}}{{\mathrm{M}}^{\mathrm{v}}}{\sum\:}_{\mathrm{i}=1}^{\mathrm{K}}{\widehat{\mathrm{Q}}}_{\mathrm{k}\mathrm{i}}$$

111 $$\:{\widehat{\mathrm{I}}}_{\mathrm{k}}\:\underrightarrow{M\to\:{\infty\:}}\:{a}_{\mathrm{m}{\prime\:}}\:\Vert\:{{\:\widehat{\mathrm{g}}}_{\mathrm{k}}\Vert\:}^{2}\:{{\upsigma\:}}^{2}\:$$

112 $$\:{\widehat{{\upgamma\:}}}_{\mathrm{k}}=\frac{{a}_{m{\prime\:}}^{2}\:{\mathrm{P}}_{s}}{{a}_{\mathrm{m}{\prime\:}}\:\Vert\:{{\:\widehat{\mathrm{g}\:}}_{\mathrm{k}}\Vert\:}^{2}{{\upsigma\:}}^{2}\:}=\frac{\:{\mathrm{E}}_{\mathrm{s}}}{{\frac{1}{{a}_{\mathrm{m}{\prime\:}}}\mathrm{M}}^{\mathrm{v}\:}\Vert\:{{\:\widehat{\mathrm{g}\:}}_{\mathrm{k}}\Vert\:}^{2}{{\upsigma\:}}^{2}}\:$$

when M is large, $\:\Vert\:{{\widehat{\mathbf{g}}}_{\mathrm{k}}\Vert\:}^{2}$ can be approximated by an identity matrix. Following this, substituting Eq. (55) into Eq. (65) leads to

113 $$\:{\widehat{{\upgamma\:}}}_{\mathrm{k}}\underrightarrow{M\to\:{\infty\:}}\:\frac{{a}_{{\mathrm{m}}^{{\prime\:}}}\:\:{\mathrm{E}}_{\mathrm{s}}\left(\mathrm{M}-\mathrm{K}+\left({\mathrm{g}}_{\mathrm{k}}-1\right)\mathrm{G}\right)}{{\mathrm{M}}^{\mathrm{v}\:}\Vert\:{{\:\widehat{\mathrm{g}\:}}_{\mathrm{k}}\Vert\:}^{2}{{\upsigma\:}}^{2}}$$

114 $$\:{\widehat{{\upgamma\:}}}_{\mathrm{k}}\approx\:\frac{{a}_{{\mathrm{m}}^{{\prime\:}}}{\mathrm{E}}_{\mathrm{s}}\left(1-\frac{(\mathrm{M}-\mathrm{K}+\left({\mathrm{g}}_{\mathrm{k}}-1\right)\mathrm{G}}{\mathrm{M}}\right)}{{\mathrm{M}}^{\mathrm{v}-1\:}{{\upsigma\:}}^{2}}\left\{\frac{{{{\upbeta\:}}_{\mathrm{k}}\mathcal{K}}_{\mathrm{k}}}{{\mathcal{K}}_{\mathrm{k}}+1}+\frac{{{{\upbeta\:}}_{\mathrm{k}}{\upeta\:}}_{\mathrm{k}\:}}{{\mathcal{K}}_{\mathrm{k}}+1}\right\}$$

115 $$\:{\widehat{{\upgamma\:}}}_{\mathrm{k}}\approx\:\frac{{{a}_{\mathrm{m}{\prime\:}}{\mathrm{E}}_{\mathrm{s}}{\upbeta\:}}_{\mathrm{k}}{\mathcal{K}}_{\mathrm{k}}\:}{{{\:\mathrm{M}}^{\mathrm{v}-1\:}}^{\:}{{\upsigma\:}}^{2}({\mathcal{K}}_{\mathrm{k}}+\:1)}\:+\frac{{{a}_{\mathrm{m}{\prime\:}}\:{\mathrm{E}}_{\mathrm{s}}{\upbeta\:}}_{\mathrm{k}}{{\upeta\:}}_{\mathrm{k}\:}}{{\mathrm{M}}^{\mathrm{v}-1\:}{{\upsigma\:}}^{2}({\mathcal{K}}_{\mathrm{k}}+1)({\frac{{\mathrm{E}}_{\mathrm{s}}}{{\mathrm{M}}^{\mathrm{v}}}\mathrm{L}\:{\upbeta\:}}_{\mathrm{k}}+\frac{{{\upsigma\:}}^{2}}{{a}_{\mathrm{m}{\prime\:}}}+\frac{(1-{a}_{\mathrm{m}{\prime\:}})}{{a}_{\mathrm{m}{\prime\:}}}\frac{{\mathrm{E}}_{\mathrm{s}}}{{\mathrm{M}}^{\mathrm{v}}}{\sum\:}_{\mathrm{i}=1}^{\mathrm{K}}{{\upbeta\:}}_{\mathrm{i}})}$$

Substituting Eq. (39) into Eq. (23), we can obtain an approximation of the SINR$\:\:{\widehat{{\upgamma\:}}}_{\mathrm{k}}\:$for the system of MRC-GSIC receivers with imperfect CSI:

116 $$\:{\widehat{{\upgamma\:}}}_{\mathrm{k}}\approx\:\frac{{{a}_{\mathrm{m}{\prime\:}}\:{\mathrm{E}}_{\mathrm{s}}{\upbeta\:}}_{\mathrm{k}}{\mathcal{K}}_{\mathrm{k}}\:}{{{\:\mathrm{M}}^{\mathrm{v}-1\:}}^{\:}{{\upsigma\:}}^{2}({\mathcal{K}}_{\mathrm{k}}+\:1)}\:+\frac{{{a}_{\mathrm{m}{\prime\:}}\:{\mathrm{E}}_{\mathrm{s}}{\upbeta\:}}_{\mathrm{k}}{{\upeta\:}}_{\mathrm{k}\:}}{{\mathrm{M}}^{\mathrm{v}-1\:}{{\upsigma\:}}^{2}({\mathcal{K}}_{\mathrm{k}}+\:1)({\frac{{\mathrm{E}}_{\mathrm{s}}}{{\mathrm{M}}^{\mathrm{v}}}\mathrm{L}\:{\upbeta\:}}_{\mathrm{k}}+\frac{{{\upsigma\:}}^{2}}{{a}_{\mathrm{m}{\prime\:}}}+\frac{(1-{a}_{\mathrm{m}{\prime\:}})}{{a}_{\mathrm{m}{\prime\:}}}\frac{{\mathrm{E}}_{\mathrm{s}}}{{\mathrm{M}}^{\mathrm{v}}}{\sum\:}_{\mathrm{i}=1}^{\mathrm{K}}{{\upbeta\:}}_{\mathrm{i}})}$$

117 $$\:{\widehat{{\upgamma\:}}}_{\mathrm{k}}\approx\:\frac{{{a}_{\mathrm{m}{\prime\:}}\:{\mathrm{E}}_{\mathrm{s}}{\upbeta\:}}_{\mathrm{k}}{\mathcal{K}}_{\mathrm{k}}\:}{{{\:\mathrm{M}}^{\mathrm{v}-1\:}}^{\:}{{\upsigma\:}}^{2}({\mathcal{K}}_{\mathrm{k}}+\:1)}\:+\frac{{{a}_{\mathrm{m}{\prime\:}}\:{\mathrm{E}}_{\mathrm{s}}{\upbeta\:}}_{\mathrm{k}}{{\upeta\:}}_{\mathrm{k}\:}}{{\mathrm{M}}^{\mathrm{v}-1\:}{{\upsigma\:}}^{2}({\mathcal{K}}_{\mathrm{k}}+\:1)({\frac{{\mathrm{E}}_{\mathrm{s}}}{{\mathrm{M}}^{\mathrm{v}}}\mathrm{L}\:{\upbeta\:}}_{\mathrm{k}}+\frac{{{\upsigma\:}}^{2}}{{a}_{\mathrm{m}{\prime\:}}}+\frac{(1-{a}_{\mathrm{m}{\prime\:}})}{{a}_{\mathrm{m}{\prime\:}}}\frac{{\mathrm{E}}_{\mathrm{s}}}{{\mathrm{M}}^{\mathrm{v}}}{\sum\:}_{\mathrm{i}=1}^{\mathrm{K}}{{\upbeta\:}}_{\mathrm{i}})}\:$$

118 $$\:{\widehat{{\upgamma\:}}}_{\mathrm{k}}\underrightarrow{M\to\:{\infty\:}}\frac{{{a}_{\mathrm{m}{\prime\:}}\:{\mathrm{E}}_{\mathrm{s}}{\upbeta\:}}_{\mathrm{k}}{\mathcal{K}}_{\mathrm{k}}\:}{{{\:\mathrm{M}}^{\mathrm{v}-1\:}}^{\:}{{\upsigma\:}}^{2}({\mathcal{K}}_{\mathrm{k}}+\:1)}\:+\frac{{a}_{m{\prime\:}}^{2}{\mathrm{E}}_{s}^{2}\:{{{\upbeta\:}}_{\mathrm{k}}}^{2}\mathrm{L}}{{\mathrm{M}}^{2\mathrm{v}-1\:}{{\upsigma\:}}^{4}({\mathcal{K}}_{\mathrm{k}}+\:1)\:}$$

For Rician fading channels, i.e.$\:\:\:{\mathcal{K}}_{k}\ne\:\:0$, the SINR tends to be

119 $$\:{\widehat{{\upgamma\:}}}_{\mathrm{k}}\underrightarrow{M\to\:{\infty\:}}\frac{{{a}_{\mathrm{m}{\prime\:}}\:{\mathrm{E}}_{\mathrm{s}}{\upbeta\:}}_{\mathrm{k}}{\mathcal{K}}_{\mathrm{k}}\:}{{{\upsigma\:}}^{2}({\mathcal{K}}_{\mathrm{k}}+\:1)}\:$$

when $\:\nu\:\:=\:1$ For Rayleigh fading, i.e. $\:{\mathcal{K}}_{k}\:=\:0$, the SINR tends to be a constant value

120 $$\:{\widehat{{\upgamma\:}}}_{\mathrm{k}}\underrightarrow{M\to\:{\infty\:}}\frac{{{{a}_{m{\prime\:}}^{2}{\mathrm{E}}_{s}^{2}{\upbeta\:}}_{\mathrm{k}}}^{2}\mathrm{L}}{{{\upsigma\:}}^{4}({\mathcal{K}}_{\mathrm{k}}+\:1)\:}\:$$

if $\:\nu\:\:=\:0.5$. When strong LoS paths exist, as $\:{\mathcal{K}}_{k}\to\:\infty\:\:$Eq. tends to be

121 $$\:{\widehat{{\upgamma\:}}}_{\mathrm{k}}\underrightarrow{M\to\:{\infty\:}}\frac{{\:\mathrm{P}}_{\mathrm{s}}{{\upbeta\:}}_{\mathrm{k}}\:(\mathrm{M}-\mathrm{K})}{(\:\frac{{{\upsigma\:}}^{2}}{{a}_{\mathrm{m}{\prime\:}}}+\frac{(1-{a}_{\mathrm{m}{\prime\:}})}{{a}_{\mathrm{m}{\prime\:}}}\:{\mathrm{P}}_{\mathrm{s}}{\sum\:}_{\mathrm{i}=1}^{\mathrm{K}}{{\upbeta\:}}_{\mathrm{i}})+[(\frac{1}{\mathrm{M}}{\mathbf{G}}^{\mathrm{H}}{\mathbf{G})}^{-1}{]}_{\mathrm{k}.\mathrm{k}}\:}\:$$

## Complexity analysis

For the proposed ΣΔ GSIC receiver, the overall signal detection complexity includes two parts: computing the receiver weights and detecting information- carrying symbols for each channel use. The receiver weights are assumed to be computed once per coherent block and then stored and reused. In the detection stage, the computation of $\:{\mathbf{g}}_{\mathrm{k}}^{\mathrm{H}}{\:\mathbf{y}}_{\mathrm{q}}\:$now involves the quantized and spatially shaped received vector. Each computation costs approximately $\:2M\:+{L}_{\boldsymbol{\Sigma\:}\boldsymbol{\Delta\:}}-\:1\:$ flops per UE, where $\:{L}_{\boldsymbol{\Sigma\:}\boldsymbol{\Delta\:}}$​ denotes the number of spatial feedback taps. Hence, the total complexity for UEs *K* is approximately ( $\:2M\:+{L}_{\boldsymbol{\Sigma\:}\boldsymbol{\Delta\:}}$ ) flops. The computation of interference-cancellation terms {$\:{\delta\:}_{ki}{.x}_{i}$} requires about $\:{k}^{2}-\:KG\:$flops. Therefore, the overall symbol detection complexity for GSIC receivers is expressed as:

122 $$\:\mathrm{C}\:\mathrm{s}\mathrm{y}\mathrm{m}\mathrm{b}\mathrm{o}\mathrm{l}\:=(2\mathrm{M}+{L}_{\boldsymbol{\Sigma\:}\boldsymbol{\Delta\:}})K\:+\:{K}^{2}\:-\:\mathrm{K}\mathrm{G}$$

For $\:\boldsymbol{\Sigma\:}\boldsymbol{\Delta\:}$ based linear receivers, only symbol detection is required, with complexity:

123 $$\:\mathrm{C}\:\mathrm{s}\mathrm{y}\mathrm{m}\mathrm{b}\mathrm{o}\mathrm{l}\:=\:(2\mathrm{M}+{L}_{\boldsymbol{\Sigma\:}\boldsymbol{\Delta\:}})K$$

For GSIC receivers, computing $\:{{\mathbf{g}}_{\mathrm{k}}^{\mathrm{H}}\:\mathbf{g}}_{\mathrm{i}}\:$costs to compute the feedback filter coefficients, correlations such as $\:{\mathbf{g}}_{\mathrm{k}}^{\mathrm{H}}{\:\mathbf{y}}_{\mathrm{q}}\:$must be evaluated within each group. Considering the additional ($\:\boldsymbol{\Sigma\:}\boldsymbol{\Delta\:}$) spatial feedback links, this operation costs approximately can be written as:

124 $$\:{\:\:\mathrm{C}}_{\mathrm{F}}\:\approx\:\:(\mathrm{M}+{L}_{\boldsymbol{\Sigma\:}\boldsymbol{\Delta\:}})\:{K}^{2}$$

flops per coherent block. Consequently, for MRC–GSIC receivers, the weight computation complexity is:

125 $$\:{{\mathrm{C}}_{\mathrm{w}\mathrm{m}}^{{\Sigma\:}{\Delta\:}}\:=\mathrm{C}}_{\mathbf{F}\:}$$

For ZF-GSIC receivers:

126 $$\:{\mathrm{C}}_{\mathrm{w}\mathrm{m}}^{{\Sigma\:}{\Delta\:}}\:=\:{C}_{G}^{ZF-GSIC}+{C}_{\mathrm{F}}$$

As noted in^23^, recursive inversion techniques, such as the Sherman–Morrison–Woodbury formula, can significantly reduce computational complexity. In the ZF–GSIC receiver, the dominant computational burden arises from matrix inversion, which typically scales with the cube of the number of users, $\:K^{3}$, for the ungrouped portion. For scalability analysis, the computation of ZF–GSIC weights can be approximated as follows:

127 $$\:{C}_{G}^{ZF-GSIC}=\:{K}^{2}\:(1-\frac{3}{2\:{N}_{G}}+\frac{3}{{2\:N}_{G}^{2}})+{K}^{2}\left(\right(2\mathrm{M}+{L}_{\boldsymbol{\Sigma\:}\boldsymbol{\Delta\:}})+\frac{25}{12{N}_{G}}-\frac{1}{3})\:$$

For the same values of $\:K,\:M$, and $\:G$, incorporating ΣΔ spatial feedback slightly increases the computational complexity due to the $\:{L}_{\varSigma\:\varDelta\:}$ term compared with conventional ADCs. Nevertheless, the resulting hardware power savings and the benefits of quantization noise shaping make GSIC receivers a compelling choice for massive MIMO systems employing low-resolution ADCs.

## MRC-GSIC receiver performance

We analyze the power allocation for systems employing MRC-GSIC, MRC, and MRC-SIC under a given QoS considering both perfect and imperfect CSI.

### With perfect CSI

Given transmission rate $\bar{R}$ the SINR for UE *k* is given by:

128 $$\:{{{\upgamma\:}}_{\mathrm{k}}\:=2}^{\frac{\stackrel{-}{R}}{B}}-1=\frac{{a}_{m{\prime\:}}^{2}{\:\mathrm{P}}_{\mathrm{s}}{\Vert\:{\mathbf{g}}_{\mathrm{k}}\Vert\:}^{4}\:}{{\mathrm{I}}_{\mathrm{k}}}$$

Substituting Eq. (44) into Eq. (128), $\:K$ linear equations with unknowns of $\:{p}_{k}$ can be written for given transmission rate $\bar{R}$ In matrix form to get power allocation as:

129 $$\:\frac{{a}_{m{\prime\:}}^{2}}{{2}^{\frac{\stackrel{-}{\mathrm{R}}}{\mathrm{B}}}\:-1}\mathbf{F}\mathbf{P}={a}_{\mathrm{m}{\prime\:}}\:{{\upsigma\:}}^{2}\mathbf{J}+{a}_{m{\prime\:}}^{2}\mathbf{Z}\:\mathbf{p}+\:{a}_{\mathrm{m}{\prime\:}}(1-{a}_{\mathrm{m}{\prime\:}})\:\mathbf{V}\mathbf{P}$$

the UEs’ power allocation can be obtained as the following:

130 $$\:{\mathbf{P}={a}_{\mathrm{m}{\prime\:}}{\upsigma\:}}^{2}\left[\right(\frac{{a}_{m{\prime\:}}^{2}}{{2}^{\frac{\stackrel{-}{\mathrm{R}}}{\mathrm{B}}}\:-1})\mathbf{F}-{a}_{m{\prime\:}}^{2}\mathbf{Z}-{a}_{\mathrm{m}{\prime\:}}(1-{a}_{\mathrm{m}{\prime\:}})\:\mathbf{V}{]}^{-1}\mathbf{J}$$

With Eqs. (78)–(81), we get the following:

131 $$\:{\mathbf{g}}_{\mathrm{k}}^{\mathrm{H}}{{\:\mathbf{R}}_{\mathbf{v}}}_{\mathrm{q}}{\:\mathrm{g}}_{\mathrm{k}}={a}_{\mathrm{m}{\prime\:}}(1\:-{a}_{\mathrm{m}{\prime\:}}\:)\left({\:\mathrm{P}}_{\mathrm{s}}{\sum\:}_{\mathrm{k}=1}^{\mathrm{K}}{{\upbeta\:}}_{\mathrm{k}}+{{\upsigma\:}}^{2}\right)$$

Thus, the vector and matrix can be approximated by:

132 $$\:{\mathrm{F}}_{\mathrm{k},\mathrm{k}}=\frac{{{\mathrm{M}{\upbeta\:}}_{\mathrm{k}}}^{2}[2{\mathrm{K}}_{\mathrm{k}}+2\mathrm{M}{\mathrm{K}}_{\mathrm{k}}+\mathrm{M}{{\mathrm{K}}_{\mathrm{k}}}^{2}+\mathrm{M}+1)}{{(\mathrm{K}}_{\mathrm{k}}\:{+\:1)}^{2}}\:,\:\:{\mathrm{J}}_{\mathrm{k}}{\approx\:\mathrm{M}{\upbeta\:}}_{\mathrm{k}}$$

$$\:\mathrm{F}\mathrm{o}\mathrm{r}\:\mathrm{k}\:>\:({g}_{n}\:-\:1)\mathrm{G}\:.\mathrm{n}\mathrm{d}\:k\:\ne\:\:i,\:\mathrm{w}\mathrm{e}\:\mathrm{h}\mathrm{a}\mathrm{v}\mathrm{e}:$$

133 $$\:{\mathrm{Z}}_{\mathrm{k}\mathrm{i}}=\frac{{{{\upbeta\:}}_{\mathrm{i}}{\upbeta\:}}_{\mathrm{k}}({\mathrm{K}}_{\mathrm{k}}{\mathrm{K}}_{\mathrm{i}}{{\Phi\:}}_{\mathrm{k}\mathrm{i}}^{2}+3\mathrm{M})}{{{(\mathrm{K}}_{\mathrm{k}}+\:1\left)\right(\mathrm{K}}_{\mathrm{k}}+\:1)}$$

The $\:(\:k,\:i\:)$th entry of V can be approximated as:

134 $$\:{\mathrm{V}}_{\mathrm{n}}{\approx\:\mathrm{M}{\upbeta\:}}_{\mathrm{k}}{{\upbeta\:}}_{\mathrm{i}\:\:}\:\:\:\:\:$$

### With imperfect CSI

Given transmission rate $\bar {R}$, the SINR for UE-*k* is given by:

135 $$\:{{\upgamma\:}}_{\mathrm{k}}={2}^{\frac{\stackrel{-}{R}}{B}}\:-1=\frac{{a}_{m{\prime\:}}^{2}{\:\mathrm{P}}_{\mathrm{s}}{\Vert\:{\:\widehat{\mathbf{g}\:}}_{\mathrm{k}}\Vert\:}^{4}}{{\widehat{\mathrm{I}}}_{\mathrm{k}}}$$

Following Eqs. (64) and (65) with similar treatment to the case under perfect CSI, we have:

136 $$\:\frac{{a}_{m{\prime\:}}^{2}}{{2}^{\frac{\stackrel{-}{R}}{B}}\:-1}\widehat{\mathbf{F}}\widehat{\mathbf{P}}={a}_{\mathrm{m}{\prime\:}}\:{{\upsigma\:}}^{2}\widehat{\mathbf{J}}+{a}_{m{\prime\:}}^{2}\widehat{\mathbf{Z}}\widehat{\mathbf{p}}+\:{a}_{\mathrm{m}{\prime\:}}(1-{a}_{\mathrm{m}{\prime\:}}\:)\widehat{\mathbf{V}}\widehat{\mathbf{P}}+\:{a}_{m{\prime\:}}^{2}\:\widehat{\mathbf{L}}\widehat{\mathbf{P}\:}$$

With the approximation results in Eq. (136) we have the following approximations:

137 $$\:\widehat{\mathbf{P}}={a}_{\mathrm{m}{\prime\:}}{{\upsigma\:}}^{2}\left[\right(\frac{{a}_{m{\prime\:}}^{2}}{{2}^{\frac{\stackrel{-}{\mathrm{R}}}{\mathrm{B}}}\:-1})\widehat{\mathbf{F}}-{a}_{m{\prime\:}}^{2}\widehat{\mathbf{Z}\:}+{a}_{\mathrm{m}{\prime\:}}(1-{a}_{\mathrm{m}{\prime\:}})\:\widehat{\mathbf{V}-{{a}_{m{\prime\:}}^{2}\widehat{\mathbf{L}}]}^{-1}\widehat{\mathbf{J}}\:}$$

With the approximation results in Eqs. (89)–(91), we have the following approximations:

138 $$\:{\widehat{\mathbf{g}}}_{\mathrm{k}}^{\mathrm{H}}{{\:\mathbf{R}}_{\mathbf{v}}}_{\mathrm{q}}{\:\widehat{\mathbf{g}\:}}_{\mathrm{k}}={a}_{\mathrm{m}{\prime\:}}(1-{a}_{\mathrm{m}{\prime\:}})\left({\:\mathrm{P}}_{\mathrm{s}}{\sum\:}_{\mathrm{k}=1}^{\mathrm{K}}{{\upbeta\:}}_{\mathrm{k}}\:{{\upsigma\:}}^{2}\right)\frac{\mathrm{M}{{\upbeta\:}}_{\mathrm{k}}{(\mathcal{K}}_{\mathrm{k}}+{{\upeta\:}}_{\mathrm{k}\:})}{{\mathcal{K}}_{\mathrm{k}}+\:1}$$

139 $$\:{\widehat{\mathrm{F}}}_{\mathrm{k},\mathrm{k}}=\frac{{{\mathrm{M}{\upbeta\:}}_{\mathrm{k}}}^{2}[2{\mathcal{K}}_{\mathrm{k}}{{\upeta\:}}_{\mathrm{k}\:}+2\mathrm{M}{\mathcal{K}}_{\mathrm{k}}{{\upeta\:}}_{\mathrm{k}\:}+\mathrm{M}{{\mathcal{K}}_{\mathrm{k}}}^{2}+(\mathrm{M}+1\left)\:{{{\upeta\:}}_{\mathrm{k}\:}}^{2}\right)}{{(\mathcal{K}}_{\mathrm{k}}\:{+\:1)}^{2}}$$

140 $$\:{\widehat{\mathrm{J}}}_{\mathrm{k}}\approx\:\frac{\mathrm{M}{{\upbeta\:}}_{\mathrm{k}}{(\mathcal{K}}_{\mathrm{k}}+{{\upeta\:}}_{\mathrm{k}\:})}{{\mathcal{K}}_{\mathrm{k}}+\:1}$$

$$\:\mathrm{F}\mathrm{o}\mathrm{r}\:\mathrm{k}\:>\:({g}_{n}\:-\:1)\mathrm{G}\:.\mathrm{n}\mathrm{d}\:\mathrm{k}\:\ne\:\:\mathrm{i},\:\mathrm{w}\mathrm{e}\:\mathrm{h}\mathrm{a}\mathrm{v}\mathrm{e}:$$

141 $$\:{\widehat{\mathrm{Z}}}_{\mathrm{k}\mathrm{i}}=\frac{{{{{{\upbeta\:}}_{\mathrm{i}}{\upbeta\:}}_{\mathrm{k}}(\mathcal{K}}_{\mathrm{k}}\mathcal{K}}_{\mathrm{i}}{{\Phi\:}}_{\mathrm{k}\mathrm{i}}^{2}+{\mathrm{M}\mathcal{K}}_{\mathrm{k}}{{\upeta\:}}_{\mathrm{i}\:}+\mathrm{M}{\mathcal{K}}_{\mathrm{i}}{{\upeta\:}}_{\mathrm{k}\:}+\mathrm{M}{{\upeta\:}}_{\mathrm{i}\:}{{\upeta\:}}_{\mathrm{k}\:})}{{{(\mathcal{K}}_{\mathrm{k}}+\:1\left)\right(\mathcal{K}}_{\mathrm{i}}+1)}\:\:\:\:\:\:\:\:\:$$

The $\:(\:k,\:i\:)-$th entry of **V** can be approximated as:

142 $$\:{\widehat{\mathrm{V}}}_{\mathrm{k}\mathrm{i}}\approx\:{{\upbeta\:}}_{\mathrm{i}}\frac{\mathrm{M}{{\upbeta\:}}_{\mathrm{k}}{(\mathcal{K}}_{\mathrm{k}}+{{\upeta\:}}_{\mathrm{k}\:})}{\mathcal{K}+\:1}$$

The $\:(\:k,\:i\:)-$th entry of **L** can be approximated as:

143 $$\:{\widehat{\mathrm{L}}}_{\mathrm{k}\mathrm{i}}={{{\upsigma\:}}^{2}}_{\mathrm{e}\mathrm{i}}\frac{\mathrm{M}{{\upbeta\:}}_{\mathrm{k}}{(\mathcal{K}}_{\mathrm{k}}+{{\upeta\:}}_{\mathrm{k}\:})}{{\mathcal{K}}_{\mathrm{k}}+\:1}$$

Similar to the equal power allocation case, when $\:{N}_{G}\:=\:1$, the above power allocation and its approximation correspond to MRC, whereas for $\:{N}_{G}\:=\:K$, they correspond to MRC-SIC.

## Numerical results

**Fig. 3:**
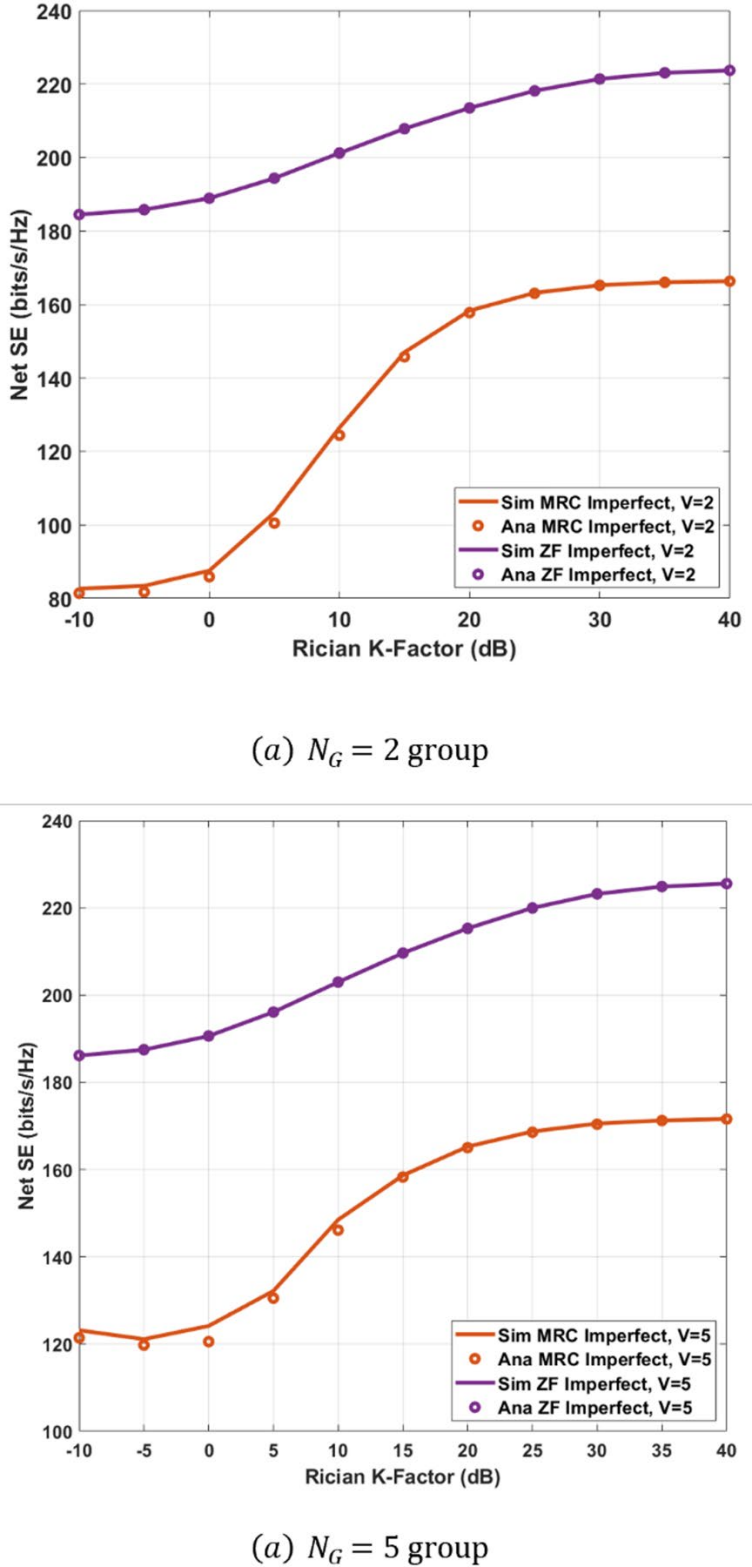
Uplink SE versus resolution 1-bit ΣΔ and Rician $\:\mathcal{K}$ -factor, with $\:M\:=\:200$ BS antennas,$\:\:K\:=\:40$ UEs, $\:{P}_{s}=\:-10\:dBm.a$

We now present numerical results to verify the analytical findings discussed in Sects.  4 and 5. The simulations consider a single-cell uplink system with $\:K\:=\:40$ UEs, where the large-scale fading coefficients βₖ follow the same settings as in^34^ and all UEs share identical Rician $\:\mathcal{K}$ -factors. A low-resolution $\:1-$bit $\:{\Sigma\:}{\Delta\:}-\mathrm{A}\mathrm{D}\mathrm{C}$ is used during both channel estimation and data transmission. Unless otherwise specified (e.g., Fig. 3), the pilot length is fixed at $\:L\:=\:K=40$ corresponding to orthogonal pilot sequences assigned to all UEs. The AoAs for the UEs are randomly generated, and the average SE is recorded. The noise power is set to $\:-94\:\mathrm{d}\mathrm{B}\mathrm{m}$, and the Rician $\:\mathcal{K}$ -factor is $\:0\:\mathrm{d}\mathrm{B}$ except in Fig. 3. Table 1summerize all parameters used in simulations.

**Table 1:** Complete system factors.

Factor	Definition	Value	Unit
$\:\boldsymbol{K}$	Number of UEs	40	#
$\:\boldsymbol{U}$	Coherence block length	1800	symbols
$\:{\boldsymbol{\zeta\:}}_{\boldsymbol{u}\boldsymbol{l}}$	Uplink activity ratio	0.4	
$\:\boldsymbol{B}$	System bandwidth	20e6	Hz
$\:\boldsymbol{L}$	Pilot length	40	
$\:{\boldsymbol{P}}_{\boldsymbol{B}\boldsymbol{S}}$	BS static RF chain power	1	W
$\:{\boldsymbol{P}}_{\boldsymbol{S}\boldsymbol{Y}\boldsymbol{N}}$	Local oscillator/synthesizer power	2	W
$\:{\boldsymbol{P}}_{\boldsymbol{o}\boldsymbol{t}\boldsymbol{h}\boldsymbol{e}\boldsymbol{r}}$	Backhaul + cooling power	0.2	W
$\:{\boldsymbol{P}}_{\boldsymbol{U}\boldsymbol{E}}$	UE transmit power	-10	W
$\:\boldsymbol{v}$	Path loss exponent	3.8	dBm
$\:{\boldsymbol{r}}_{\boldsymbol{h}}$	Reference distance	100	m
$\:\boldsymbol{r}\boldsymbol{a}\boldsymbol{d}\boldsymbol{i}\boldsymbol{u}\boldsymbol{s}$	Cell-edge radius	200	m
$\:{\mathcal{K}}_{\boldsymbol{d}\boldsymbol{B}}$	Rician K-factor	0	dB
$\:{\boldsymbol{\sigma\:}}^{2}$	Noise power	-94	dBm

### Spectral efficiency performance

**Fig. 4:**
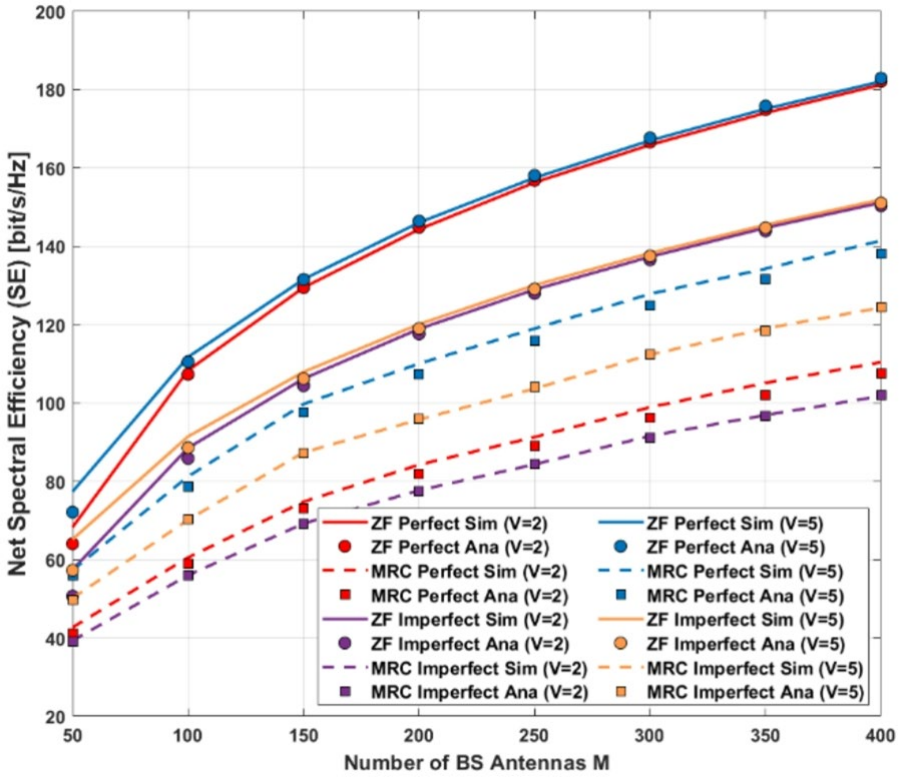
Uplink SE versus number of BS antennas with K = 40 UEs, $\:{\:\mathrm{P}}_{\mathrm{s}}$= −10 dBm, and resolution 1-bit ΣΔ at the BS.

This subsection evaluates the spectral efficiency (SE) performance of the proposed system employing low-resolution ΣΔ-ADCs and GSIC receivers under an equal power allocation strategy. All UEs are assumed to transmit with identical power during both the pilot and data transmission phases. The net SE is then calculated according to a predefined quality-of-service (QoS) requirement for all UEs. Figure 4 illustrates the uplink SE as a function of the number of BS antennas $\:M$. Equal power allocation is applied with a per-user transmit power of $\:{P}_{s}=-10$dBm, and 1-bit ΣΔ-ADCs are employed at the BS. The total SE, defined as the sum of the SEs of all UEs, is evaluated for MRC-GSIC and ZF-GSIC receivers under both perfect and imperfect CSI, over Rician fading channels, and for different numbers of GSIC groups. The results reveal a strong agreement between the analytical and simulation results, validating the accuracy of the derived SE expressions. Under both CSI conditions, the SE of all receivers increases monotonically with the number of BS antennas. Moreover, the ZF-GSIC receiver consistently outperforms the MRC-GSIC receiver, owing to its superior inter-user interference suppression capability. Increasing the number of GSIC groups further enhances the system SE, as the successive group interference cancellation process enables more effective mitigation of residual interference.

Figure 3 illustrates the SE performance of systems employing $\:1-$bit spatial ΣΔ-ADCs under different Rician $\:\mathcal{K}-$factors and imperfect CSI. The CSI is obtained using the LMMSE estimator. Moreover, the results compare low-resolution ΣΔ$\:-$ADCs with ideal ADCs that introduce negligible quantization noise. As the Rician $\:\mathcal{K}-$factor increases, the SE achieved by systems with MRC-GSIC receivers gradually approaches that of ZF-GSIC receivers. This trend is consistent with the analysis in Sect.  4.2: when the LoS component is strong, the dominant inter-user interference (IUI) originates from UEs whose AoAs are closely spaced. In this scenario, the ΣΔ architecture shapes and suppresses quantization noise in the directions where interference is most significant, thereby enhancing the performance of MRC-GSIC detection. Additionally, the performance loss associated with low-resolution ΣΔ$\:-$ADCs is notably reduced when either the number of GSIC groups or the Rician $\:\mathcal{K}-$factor increases, highlighting the robustness of the ΣΔ framework in spatially correlated propagation environments.

**Fig. 5:**
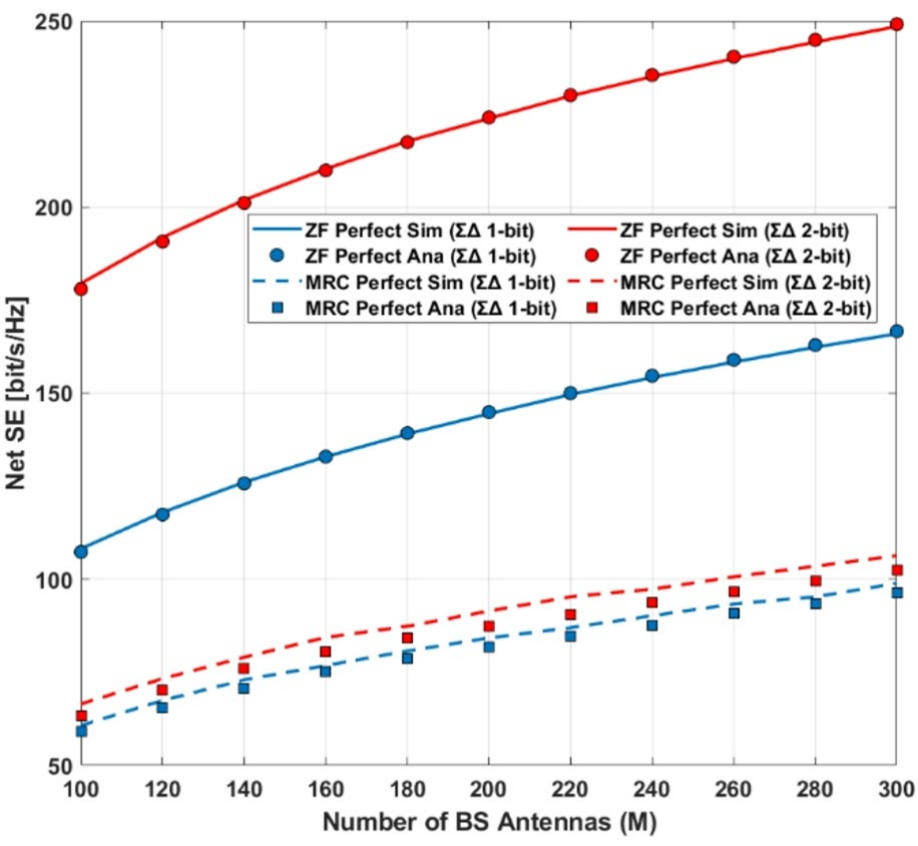
Uplink SE versus (1–2) bit a Sigma–Delta (ΣΔ) and number of BS antennas, with K = 40 UEs, $\:{\:\mathrm{P}}_{\mathrm{s}}$= −10 dBm.

Figure 5 further examines the uplink SE performance for ΣΔ$\:-$ADCs with different quantization resolutions using both MRC and ZF receivers. The results confirm that higher-resolution ΣΔ$\:-$ADCs (e.g., 2-bit) provide a substantial SE improvement compared with $\:1-$bit ADCs due to the lower noise quantization level. As expected, the ZF$\:-$GSIC receiver consistently outperforms the MRC$\:-$GSIC receiver because of its stronger capability to eliminate inter-user interference. Nevertheless, the MRC$\:-$GSIC receiver still achieves competitive SE, demonstrating its effectiveness even under the constraints imposed by low-resolution ΣΔ$\:-$ADCs.

**Fig. 6:**
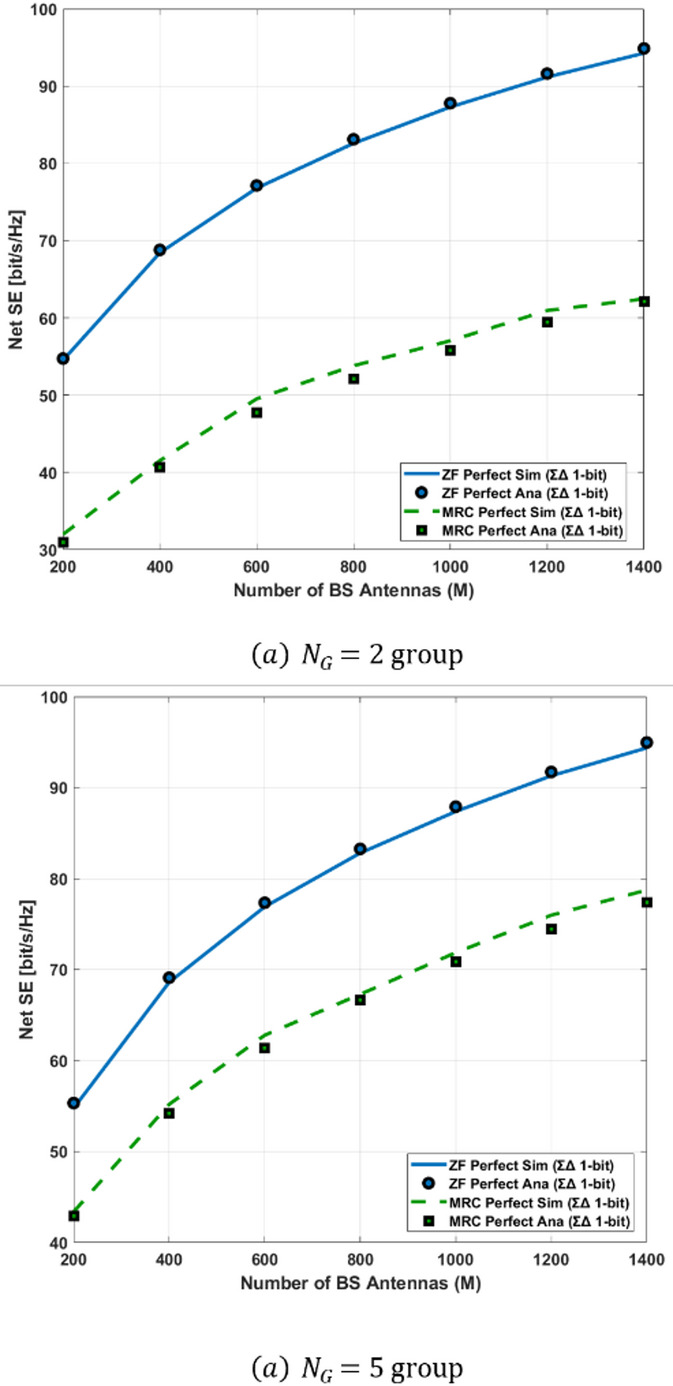
Demonstration of the power-scaling laws with $\:K\:=\:40$ UEs, $\:{\mathrm{E}}_{\mathrm{s}}$ = 0 dBm, 1-bit a Sigma–Delta (ΣΔ) at the BS.

We are now investigating the power scaling law discussed in Sect.  4.2. As shown in Fig. 6, when the number of BS antennas $\:M$ becomes very large, the SE of the system converges to a constant value. This observation confirms the analytical result in Sect.  4.2, which states that the transmit power can be reduced proportionally to $\:\frac{1}{M}$ while still maintaining a fixed SE. Moreover, the results demonstrate that, for sufficiently large $\:M$, the SE achieved by both ZF$\:-$GSIC and MRC$\:-$GSIC receivers converges to the same value, regardless of the number of GSIC groups.

**Fig. 7:**
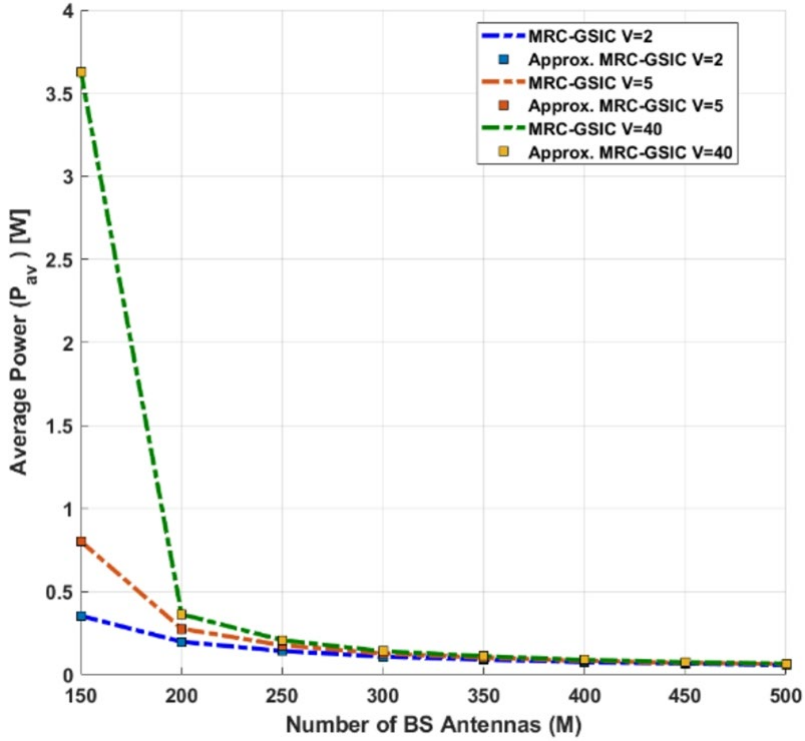
Given a QoS (SE = 2bits∕s∕Hz for each UE), the average power of a system with $\:2-$bits $\:{\Sigma\:}{\Delta\:}$, $\:K\:=\:40\:$UEs versus number of BS antennas.

Consequently, Fig. 7 presents the average total uplink transmit power of the system as a function of the number of BS antennas, considering different numbers of GSIC groups. The transmit power allocated to each UE is determined based on the required QoS constraint and can be computed using the following expression:

144 $$\:{P}_{k}^{ee}=\frac{\omega\:\sigma\:}{{\beta\:}_{k}}$$

Here, $\:\omega\:$ represents the QoS or SINR target, and $\:\sigma^{2}$ denotes the noise variance. As observed in the figure, the average transmits power decreases with an increasing number of BS antennas, enabling each UE to achieve the target SINR with lower power. Overall, the results in Fig. 7 confirm that, for a given QoS requirement, the transmit power can be effectively reduced by increasing the number of BS antennas or the number of GSIC groups.

### Energy efficiency performance

We also consider the EE performance measured by the number of bits transmitted per Joule

145 $$\:\mathrm{E}\mathrm{E}=\frac{\sum\:_{\mathrm{k}=1}^{\mathrm{K}}{\mathrm{R}}_{\mathrm{k}}}{{P}_{cons}}\:$$

where $\:{R}_{k}$ denotes the uplink net transmission rate of UE-*k* and $\:P$ is the average power consumption. Following^25^, we assume coherence blocks of $\:U\:=\:1800\:$symbols and uplink ratio$\:\:{\zeta\:}_{ul}$ = 0.4. Thus, $\:U{\zeta\:}_{ul}$*=* 720 symbols are transmitted in the uplink in each coherence block. Given the pilot length $\:L.$and the bandwidth $\:B\:=\:20\:\mathrm{M}\mathrm{H}\mathrm{z}$, the effective uplink transmission rate of UE-*k* and the uplink net transmission rate $\:{R}_{k}$ can be calculated by.

146 $$\:{\:\mathrm{R}}_{\mathrm{k}}\:={{\upzeta\:}}_{\mathrm{u}\mathrm{l}}\:(\:1\:-\:\frac{\mathrm{L}}{\mathrm{U}{{\upzeta\:}}_{\mathrm{u}\mathrm{l}}})\:{\stackrel{-}{\mathrm{R}}}_{\mathrm{k}}\:B$$

where $\:(1\:-\:\frac{L}{U{\zeta\:}_{ul}})\:$ characterizes the training overhead. the total average power consumption is given by^31^:

147 $$\:{P}_{\mathrm{c}\mathrm{o}\mathrm{n}\mathrm{s}}=M({P}_{BS}\:+\:2{P}_{ADC}^{\varSigma\:\varDelta\:})\:+\:K{P}_{UE}\:+{P}_{SYN}\:+\:{P}_{other}+\:{P}_{CD}\:+\:{P}_{CE}\:+\:\:{P}_{SC}^{\varSigma\:\varDelta\:}+{P}_{TX}^{\varSigma\:\varDelta\:}$$

Where$\:\:{P}_{UE}\:,{P}_{SYN}\:,\:{P}_{BS},\:{P}_{CD}\:\:$are the $\:\mathrm{R}\mathrm{F}\:$ circuit power consumption per RF chain at the BS, the total circuit power consumption of each UE’s device, the power of the local oscillator at the BS, the power for channel coding and decoding respectively. $\:{P}_{other}\:$represents the backhaul power consumption and the power of site cooling, etc. The values of $\:{P}_{UE}\:,{P}_{SYN}\:,\:{P}_{BS},\:{P}_{CD}\:\:$ and $\:{P}_{other}$ given in Table 1. Besides, $\:{P}_{ADC}^{\varSigma\:\varDelta\:}$ is the power consumed by a $\:{b}_{low}\:$is the actual number of bits in the low-resolution quantizer ($\:{\mathrm{b}}_{\mathrm{l}\mathrm{o}\mathrm{w}}$=1 or 2 bit) and can be evaluated as:^31^

148 $$\:{P}_{ADC}^{\varSigma\:\varDelta\:}\:=\:{FOM}_{\varSigma\:\varDelta\:}\:\:{f}_{s}\:\:{2}^{{b}_{low}}+{P}_{FB\:}$$

where the figure of merit of the $\:{\Sigma\:}{\Delta\:}-$ADC ($\:{\mathrm{F}\mathrm{O}\mathrm{M}}_{{\Sigma\:}{\Delta\:}}$) means the energy consumed per conversion step, $\:{f}_{s}$ the sampling rate and $\:{P}_{FB\:}$is the additional power consumed by the spatial feedback loop circuitry (integrators/delays, adders, and the Digital-to-Analog Converter (DAC) in the loop) within the $\:{\Sigma\:}{\Delta\:}$ modulator at each antenna element. In the following simulations, we set the value of $\:\mathrm{F}\mathrm{O}\mathrm{M}=1432.1\mathrm{e}-15\:\:\mathrm{J}/\mathrm{c}\mathrm{o}\mathrm{n}\mathrm{v}-\mathrm{s}\mathrm{t}\mathrm{e}\mathrm{p}$ and consider Nyquist sampling rate to model commercialized ADCs. The digital signal processing $\:\left(\mathrm{D}\mathrm{S}\mathrm{P}\right)\:$power consumption in the channel estimation is given by^35^:

149 $$\:{P}_{SC}^{\varSigma\:\varDelta\:}\:=\:B\:{\zeta\:}_{ul}\:(\:1\:-\:\frac{L}{{\zeta\:}_{ul}U}\:)\:\frac{{C}_{symbol}}{{L}_{BS}\:}\:+\:{P}_{BL}^{\varSigma\:\varDelta\:}$$

where$\:{\:\mathrm{L}}_{\mathrm{B}\mathrm{S}}$ represents the computational efficiency at the BS $\:{L}_{BS}$ = 12.8 Gflops/W, and $\:{\mathrm{C}}_{\mathrm{s}\mathrm{y}\mathrm{m}\mathrm{b}\mathrm{o}\mathrm{l}}$ is the complexity of recovering a symbol. The computational power consumed due to the filter calculation, $\:{\mathrm{P}}_{\mathrm{B}\mathrm{L}}^{{\Sigma\:}{\Delta\:}}\:$, is given as:

150 $$\:{P}_{BL}^{\varSigma\:\varDelta\:}\:=\:\frac{B\:{C}_{wm}^{\varSigma\:\varDelta\:}\:}{U\:{L}_{BS}}$$

where $\:{C}_{wm}^{\varDelta\:\varSigma\:}$being the complexity per user includes the feedback weight update of the ΣΔ modulator. The total uplink transmission power of the ΣΔ system, including both pilot and data components $\:{P}_{TX}^{\varDelta\:\varSigma\:}\:$ is given by^31^:

151 $$P_{{TX}}^{{\Sigma \Delta }}~~=~~~\underbrace {{\frac{{B~L~}}{{U\eta }}~K{P_t}}}_{{Pilot~~power}}+\underbrace {{\frac{{B~{\zeta _{ul}}}}{{\eta ~~}}~\left( {~1~ - ~\frac{{L~}}{{U{\zeta _{ul}}~}}} \right)K~{P_s}}}_{{data~~power}}$$

**Fig. 8:**
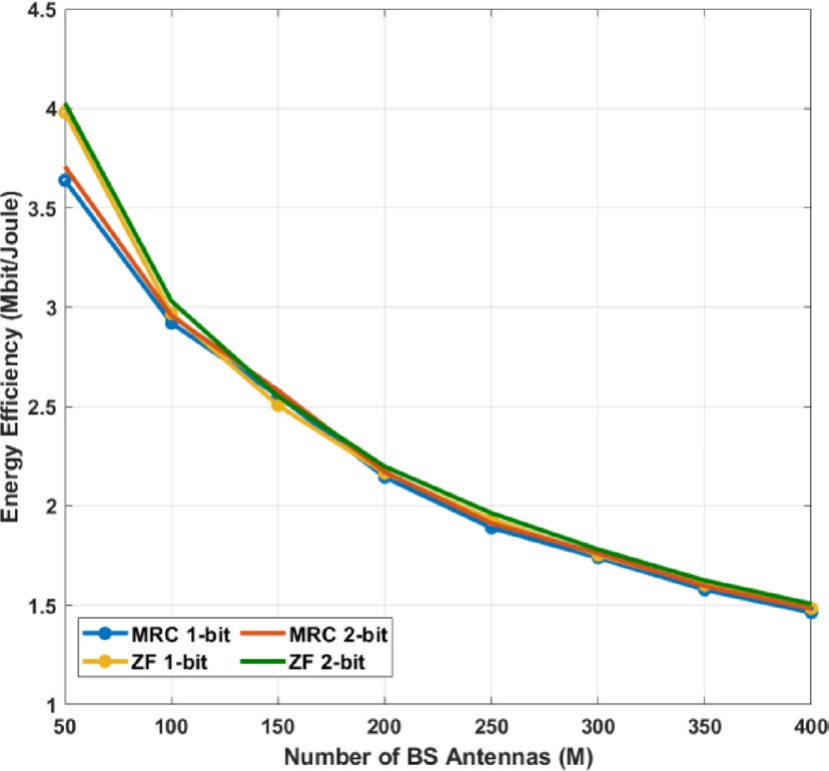
Uplink EE versus the number of antennas with $\:K\:=\:40$, $\:{P}_{s}\:=\:-10$ dB and Rician factor = 0 dB. with 1 and $\:2-$bit a ΣΔ.

Finally, Fig. 8 depicts the achievable EE of the system for different numbers of BS antennas under Rician fading. It can be observed that the EE does not increase monotonically with the number of antennas; in fact, it may decrease when the system has a very large number of antennas. This behavior is attributed to the increased RF circuit power consumption in large-scale antenna systems.

The following section is extension of our work to ensure the validation of our work in different channel models such as Nakagami m- fading.

### Validation on Nakagami-m fading

The Nakagami-m distribution in Eq. (152) is a generalized model that can represent various fading conditions by adjusting the shape parameter m where,

- *For Rayleigh Fading*: Occurs when m = 1.
- *Rician Fading*: Can be closely approximated by Nakagami-m when m > 1.

The relationship between the Rician $\:\mathcal{K}$ -factor and the Nakagami-m parameter is generally given by$\:\:\mathbf{m}=\frac{{(\mathcal{K}+1)}^{2}}{2\mathcal{K}+1}$.

c) *Severe Fading*: Occurs when m < 1.

152 $$\:f(r;m,{\Omega\:})=\frac{{2m}^{m}{r}^{2m-1}}{\varGamma\:\left(m\right){{\Omega\:}}^{m}}exp(\left(-\frac{m{r}^{2}}{{\Omega\:}}\right)$$

**Fig. 9:**
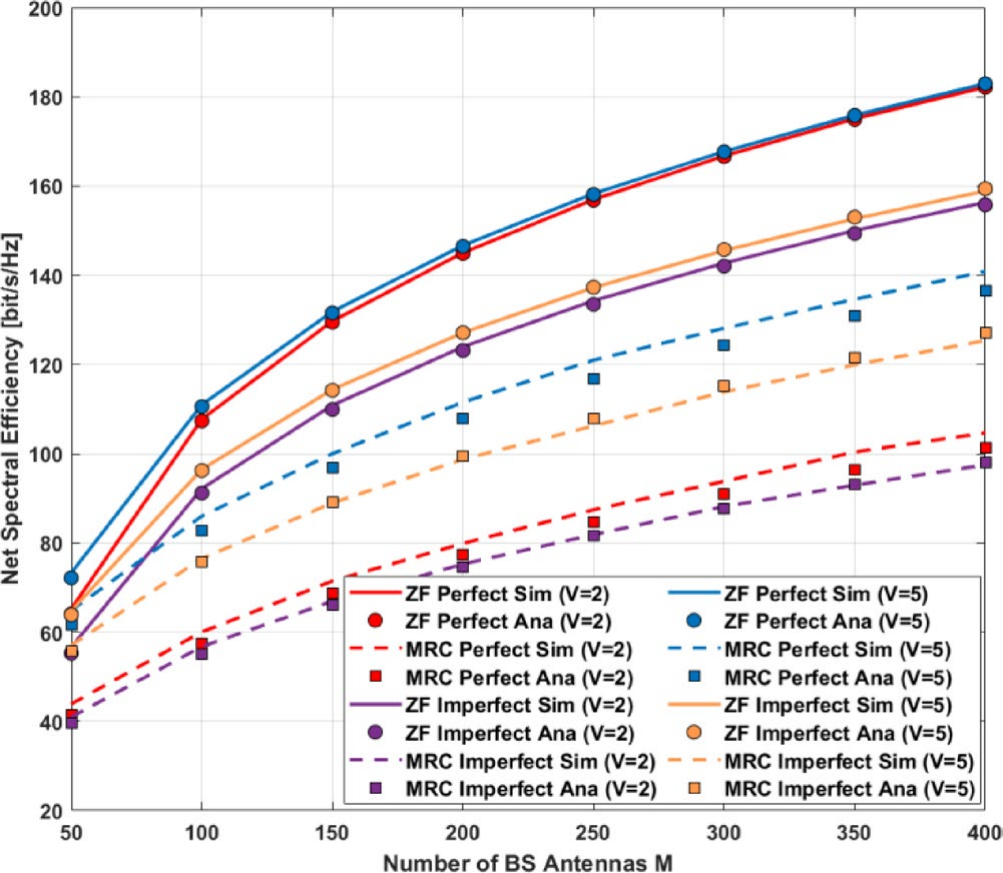
Uplink SE versus the number of antennas with $\:K\:=\:40$, $\:{P}_{s}\:=\:-10$ dB for Nakagami-m Fading with 1- bit ΣΔ.

Because theoretical results for the Rician model align with the Nakagami-m results, you have effectively shown that the oversampling and noise-shaping gains of the 1-bit ΣΔ ADC are dominant over the specific statistical distribution of the fading, provided the average received power is consistent. The proposed 1-bit ADC quantization framework exhibits significant statistical robustness across diverse fading environments. While the initial derivation focused on Rician channels to account for Line-of-Sight (LoS) components, numerical results in Fig. 9 indicate that the SE remains invariant when extended to Nakagami-m ΣΔ channels with equivalent parameters (m > 1). This convergence suggests that the spatial/temporal oversampling gain and the quantization noise-shaping characteristics of the modulator are the primary drivers of performance, effectively mitigating the sensitivity to specific small ΣΔ -scale fading distributions. Consequently, the derived closed-form expressions for Rician channels serve as a tight lower bound for generalized Nakagami-m fading scenarios.

### Dense multipath scenario

**Fig. 10:**
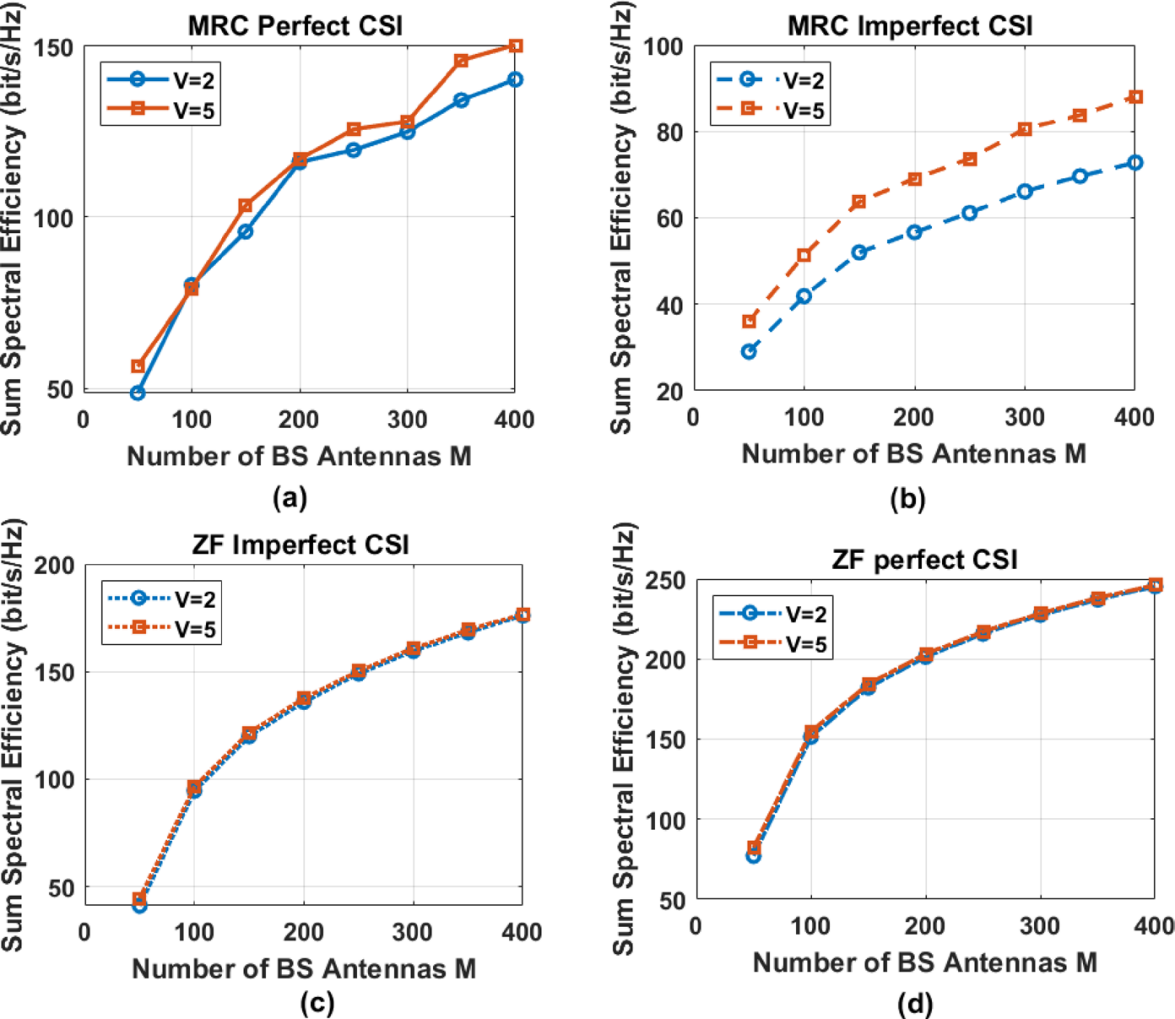
Impact of dense multipath (urban microcells) on Σ-Δ 1-bit ADCs (L = 20 paths, Angular spread = 15°).

Dense multipath introduces a “rich scattering” environment where each user signal arrives from many different angles, complicating the spatial filtering process. The analysis of the simulation results of ZF vs. MRC Performance confirms that ZF outperforms MRC in these dense environments. ZF is more effective at suppressing IUI caused by signals spreading across multiple paths. In addition, $\:\:{N}_{G\:}$changing comparison in Fig. 10 indicates that the increasing in the number of GSIC groups (from $\:{N}_{G\:}$=2 to $\:{N}_{G\:}$=5) elevates the overall SE floor for all configurations. The performance gap between low-resolution ΣΔ-ADCs and higher-resolution uniform ADCs is further reduced as the number of groups increases. Furthermore, the effect of CSI under dense multipath, channel estimation is more challenging. The code reflects this by reducing the estimation quality factor ($\:\eta\:$ = 0.7 for dense vs. 0.8 for sparse), which leads to lower SE in the “Imperfect CSI” plots. In addition, angular spread and spatial bleeding is discussed. The core mechanism of spatial ΣΔ-ADC is to “push” quantization noise into spatial frequencies where no users are present. An angular spread of 15° is used, meaning users are clusters rather than point sources. Consequently, as users occupy more of the angular spectrum, there are fewer “empty” spatial areas to move noise into. This makes quantization noise shaping less efficient because the noise is more likely to overlap with actual signal paths. Finally, resilience through antenna scaling, despite the challenges of dense multipath, the simulation shows that the system remains robust if the antenna count ($\:M$) is high. Where increasing $\:M$ from 50 to 400 provides the resolution needed to distinguish between multiple paths even in rich scattering environments. Also, the Sum SE continues to grow with $\:M$ without immediate saturation, proving that Massive MIMO serves as a powerful countermeasure to the degradations caused by urban multipath. So, dense multipath significantly affects the scheme by reducing the efficiency of spatial noise shaping and making channel estimation more difficult. However, architecture remains viable and scales effectively as the number of antennas increases, particularly when using ZF processing.

In the following subsection, the effect of different Rician factors on every user is discussed.

### Impact of heterogeneous channels and spatial correlation on SE performance

In practice, propagation conditions are highly heterogeneous, as summarized below:

- Users close to the BS with a clear LoS) typically experience strong Rician components (high $\:\mathcal{K}$ -factors), whereas cell edge users or users subject to blockage often exhibit low or near-Rayleigh $\:\mathcal{K}$ -factors.
- In our simulations, user-specific K-factors are generated based on distance, angle of arrival, and LoS probability, resulting in a wide and realistic distribution.
- Path loss and shadowing vary significantly across users. This is explicitly modeled as:

153 $$\:{\beta\:}_{k}={z}_{k}{\left(\frac{{r}_{k}}{{r}_{h}}\right)}^{-v}$$

where $\:{z}_{k}$represents log-normal shadowing and $\:{r}_{k}$is the distance between the BS and the $\:k$-th UE. In addition, $\:v$ is the path-loss exponent.

- Antenna correlation depends on angular spread, propagation environment, and LoS/NLoS conditions. Ignoring spatial correlation may lead to overestimation of spectral efficiency, particularly for LoS users with narrow angular spreads.
- In contrast, our model incorporates physically grounded spatial correlation matrices that account for antenna spacing, wavelength, mean angle of arrival, angular spread, user distance, and LoS/NLoS conditions.

For each user, a spatial correlation matrix is constructed using a Gaussian angular spread model for a ULA:

154 $$\:{\mathrm{R}}_{\mathrm{n}}(\mathrm{m},\mathcal{l})={\mathrm{e}}^{\frac{\mathrm{j}2{\uppi\:}}{{\uplambda\:}}(\mathrm{m}-\mathcal{l})\mathrm{d}\mathrm{s}\mathrm{i}\mathrm{n}{{\uptheta\:}}_{\mathrm{n}}}{\mathrm{e}}^{-\frac{1}{2}{\left(\frac{2{\uppi\:}}{{\uplambda\:}}\right(\mathrm{m}-\mathcal{l})\mathrm{d}{{\upsigma\:}}_{{\uptheta\:}}\mathrm{c}\mathrm{o}\mathrm{s}{{\uptheta\:}}_{\mathrm{n}})}^{2}}$$

where: $\:m-l$ =0, 1,…,M−1, $\:d$ is the antenna spacing, $\:{\theta\:}_{n}$​ denotes mean AoA of user n and $\:{\sigma\:}_{\theta\:}$​ gives angular spread in radians. These matrices are normalized and used directly in both channel generation and analytical SINR expressions. In addition, angular spread is explicitly modeled via $\:{{\upsigma\:}}_{{\uptheta\:}}$. The impact of angular spread is reflected through:

- Reduced spatial correlation.
- Effective rank $$\:{\mathrm{M}}_{\mathrm{e}\mathrm{f}\mathrm{f}}=\frac{{(\mathrm{t}\mathrm{r}\hspace{0.17em}{\mathrm{R}}_{\mathrm{n}})}^{2}}{\mathrm{t}\mathrm{r}\left({\mathrm{R}}_{n}^{2}\right)}$$.
- Correlation-aware analytical approximations for MRC and ZF.

While the angular spread is fixed in the current study, its effect is fully captured through the spatial correlation matrices, rather than being ignored.

**Fig. 11:**
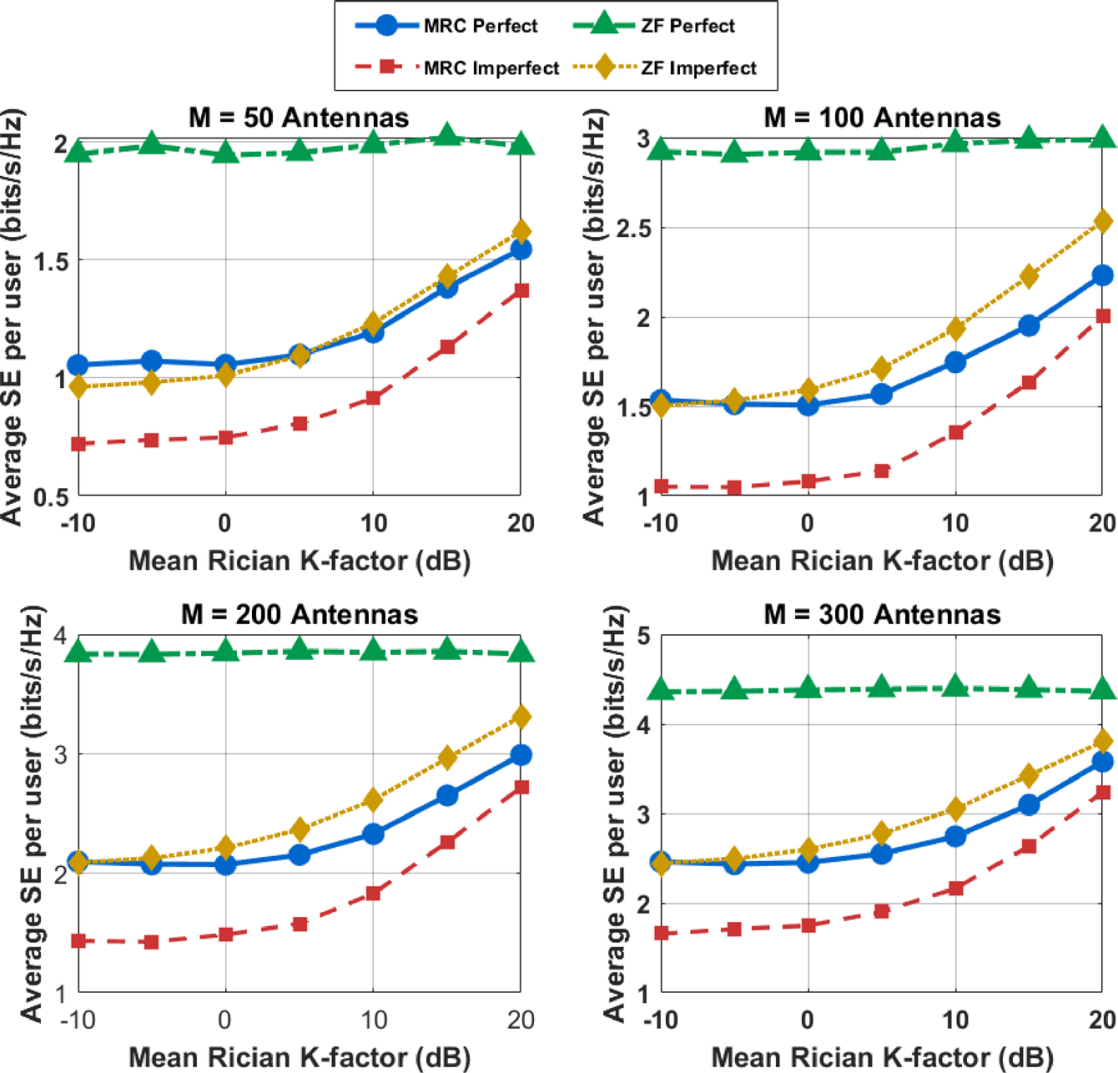
Massive MIMO performance with heterogeneous propagations for UEs = 40 users, 1-bit Σ-Δ ADC and V = 2.

As shown in Fig. 11, the simulated user-specific $\:\mathcal{K}$ -factors span a wide range due to variations in distance, blockage, mobility, and environment type. This heterogeneity directly impacts SE and alters the relative performance of linear receivers such as MRC and ZF. Furthermore, the results demonstrate that while increasing the mean $\:\mathcal{K}$ -factor improves SE, the gain saturates under realistic conditions, including imperfect CSI, spatial correlation, and coarse Σ-Δ quantization. This highlights the importance of realistic modeling, as simplified assumptions of identical $\:\mathcal{K}$ -factors and i.i.d. fading can produce overly optimistic performance predictions. Finally, our SE-versus- $\:\mathcal{K}$ -factor results confirm that although ZF generally outperforms MRC, it is also more sensitive to CSI imperfections, suffering significant performance degradation under heterogeneous and correlated channels. Increasing the number of BS antennas improves SE; however, the gains are non-linear due to spatial correlation and ADC distortion effects. These observations validate the necessity of incorporating heterogeneous propagation conditions and imperfect CSI in system-level performance evaluation.

The sensitivity of ΣΔ-ADC of spatial correlation arises due to the nature of spatial oversampling and noise shaping mechanisms inherent in Σ-Δ ADC architecture.

1. Spatial correlation characterizes the similarity between signals received at different antenna elements and is governed by the angular spread of incoming multipath components and the users’ spatial distribution. As seen in the simulation model, the channel correlation matrices are generated using a Laplacian Angular Power Spectrum (APS) with a defined angular spread $\:({\sigma\:}_{\theta\:}$).

- High spatial correlation (narrow angular spread) implies that signals at adjacent antennas are strongly correlated, reducing the effective spatial diversity. For ΣΔ ADCs, which exploit spatial oversampling, this results in diminished noise-shaping gain since correlated inputs reduce the effective quantization noise suppression achievable across the antenna array.
- Conversely, low spatial correlation (wide angular spread) increases the decorrelation between antenna signals, allowing the ΣΔ quantization noise to be better shaped and suppressed spatially, thus improving effective Signal-to-Quantization-Noise Ratio (SQNR).

2. The antenna array configuration, particularly ULAs with $\:\lambda\:/2$ spacing directly affects the spatial sampling of the incoming wave fronts.

- The antenna spacing and layout determine the spatial Nyquist sampling rate. Σ-Δ ADC schemes rely on oversampling in the spatial domain to push quantization noise out of the signal subspace.
- Inadequate spacing or array size leads to spatial aliasing or insufficient oversampling, impairing the noise-shaping effect and resulting in increased quantization noise in the useful signal subspace.
- Additionally, the array size $\:M$ modulates the degrees of freedom available for spatial filtering and noise shaping. Larger arrays enhance spatial oversampling, yielding improved Σ-Δ ADC performance, as evidenced by increasing spectral efficiency trends with MMM in your simulation results.

3. The intricate interplay between spatial correlation and array geometry means that the ΣΔ-ADC design must be carefully tailored to the propagation environment and antenna configuration:

- Environments with rich scattering and large angular spreads naturally complement ΣΔ-ADCs by providing decorrelated antenna inputs.
- In scenarios with strong line-of-sight (LoS) components or clustered angular arrivals, spatial correlation degrades the noise shaping benefit, necessitating optimization of antenna spacing or adoption of advanced signal processing techniques.
- Moreover, the presence of spatial correlation influences the channel estimation accuracy and hence the overall system performance under practical imperfect CSI conditions, as reflected in simulation loops.

4. The presented simulation framework incorporates spatial correlation effects in massive MIMO uplink channels with ΣΔ ADCs and compares SE performance under both perfect and imperfect CSI scenarios, using MRC and ZF combining schemes. While the study captures key realistic aspects such as Laplacian APS-based spatial correlation and shadow fading, the results tend to overstate the robustness of ΣΔ-ADCs in realistic correlated channels, for the following reasons:

- The quantization distortion covariance is approximated by a diagonal matrix $\:{{\mathbf{R}}_{\mathrm{v}}}_{\mathrm{q}}=\:\mathbf{A}\left(1-\mathbf{A}\right)\mathrm{d}\mathrm{i}\mathrm{a}\mathrm{g}\:\left(.\right)\:$neglecting off-diagonal spatial correlation in quantization noise. In practice, ΣΔ ADCs induce spatially correlated quantization noise due to noise shaping and oversampling effects, which can degrade performance significantly in correlated antenna arrays.
- The model assumes fixed and ideal correction factors (β) and fixed ADC resolution without accounting for implementation impairments, non-idealities, or mismatch effects which could amplify quantization noise and reduce effective robustness in real deployments.
- Although a Laplacian APS is used, the channel spatial correlation matrix approximation relies on Gaussian assumptions for the angular spread decay. This simplification may underestimate sidelobe and angular spread effects that can increase interference and channel estimation error, affecting ΣΔ ADC performance.
- The simulation considers a narrowband flat-fading channel snapshot. Realistic massive MIMO systems experience temporal and frequency selectivity, which interact with ADC noise shaping properties and could further degrade performance, especially under correlated fading.
- The analytical expressions for SE are set as placeholders equal to simulation results, omitting detailed quantification of quantization noise and channel estimation errors in closed form. This limits the ability to rigorously evaluate the impact of spatial correlation and ADC design on robustness.

### Impact of high mobility on system performance

**Fig. 12:**
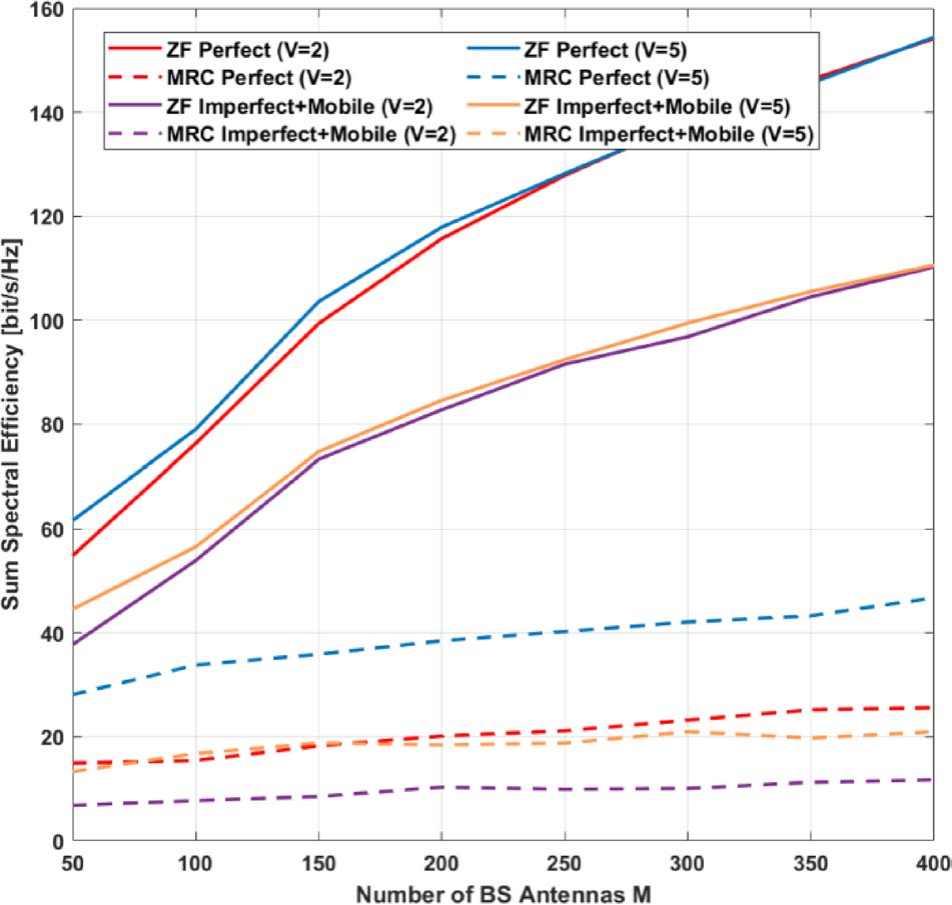
Uplink SE for perfect and imperfect CSI with mobility at high user speeds = 30 m/s and $\:\rho\:=-0.18$.

**Fig. 13:**
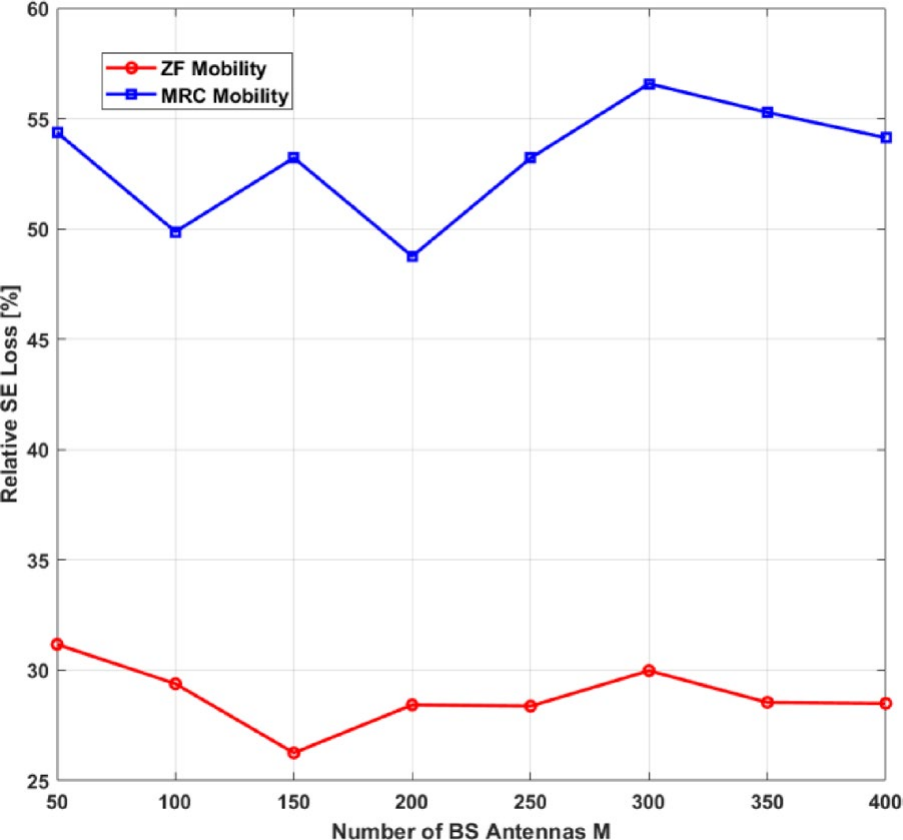
Relative SE loss caused by mobility for ZF and MRC receivers at velocity = 30 m/s with $\:{\sigma\:}_{\theta\:}=15^\circ\:$ and $\:{\:N}_{G\:}=2$.

The following results illustrate the sum SE as a function of the number of BS antennas $\:M$ under different mobility scenarios and receiver techniques. Figure 12 compares SE under perfect and imperfect CSI at high user speeds (30 m/s), revealing a noticeable performance gap between ideal and realistic conditions. Furthermore, Fig. 13 presents the relative SE loss caused by mobility for ZF and MRC receivers at a velocity of $\:v=30$m/s. The results indicate an average SE loss of approximately 28.8% for ZF and 53.2% for MRC due to mobility, with the degradation becoming more pronounced as the number of BS antennas increases. This behavior confirms that channel aging significantly impacts the reliability of CSI, reducing system performance in high-mobility scenarios. Thus, while the system achieves high SE under static or low-mobility conditions, CSI degradation at higher velocities limits its practical applicability in V2X scenarios. Future work will investigate robust or predictive CSI techniques to mitigate mobility-induced performance losses.

**Fig. 14:**
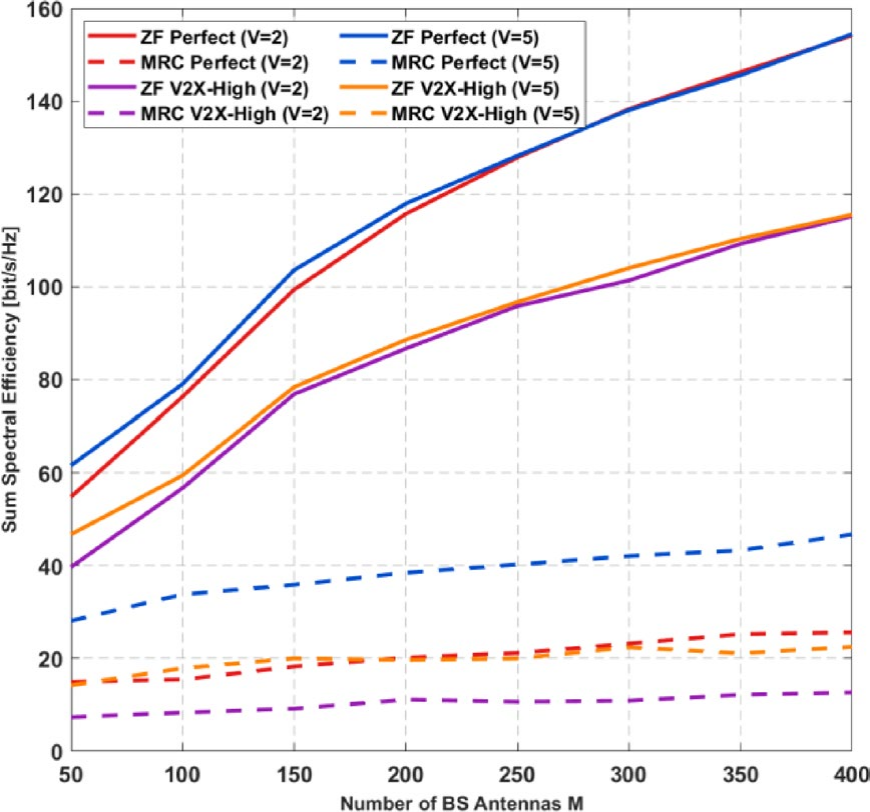
Uplink SE for V2X-high mobility of 50 m/s (180 km/h) and $\:\rho\:\:=\:-0.14$.

In addition, the results presented in Fig. 14 explicitly consider a high-mobility V2X scenario, modeled at a velocity of 50 m/s (180 km/h) with spatial correlation $\:\rho\:=-0.14$. Figure 14 demonstrates a significant degradation in sum SE when comparing perfect CSI with high-mobility V2X conditions, reflecting the rapid deterioration of CSI accuracy in such environments. Although increasing the number of base station antennas improves SE in both cases, a consistent performance gap remains due to CSI aging.

**Fig. 15:**
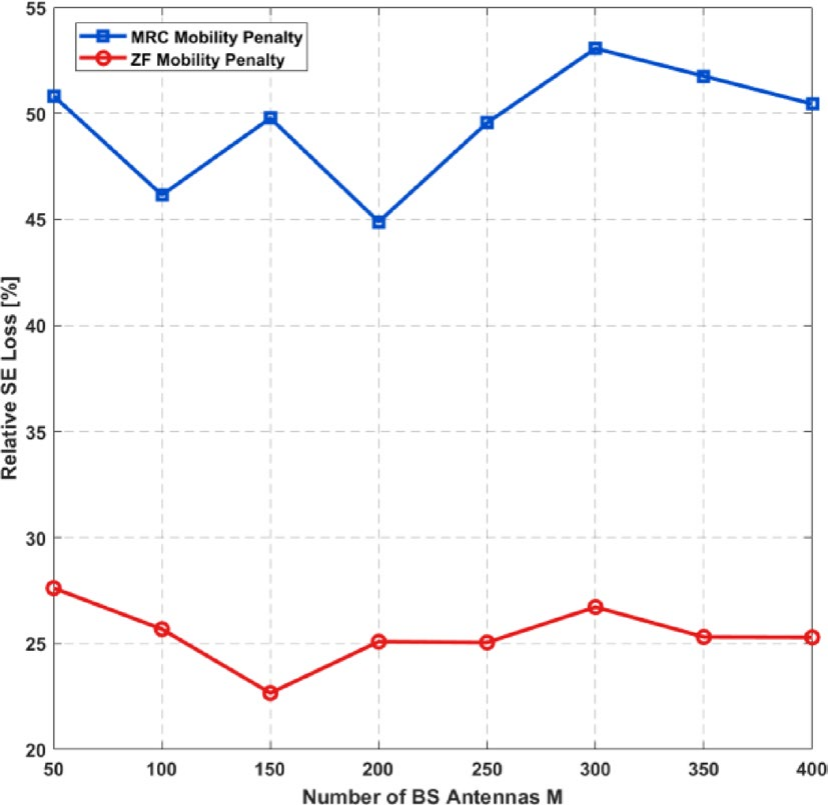
Relative SE loss caused by mobility for ZF and MRC receivers at V2X-high mobility of 50 m/s (180 km/h).

Figure 15 further quantifies this effect, showing a relative SE loss of approximately 25% for ZF receivers and nearly 50% for MRC receivers, underscoring the substantial penalties imposed by high mobility. These findings confirm that the proposed models effectively capture the impact of CSI aging under realistic high-mobility and V2X conditions.

To model user mobility, we adopt a first-order Gauss–Markov channel aging model. The time-varying channel vector of the $\:k$-th user at time slot $\:t$is expressed as^52^

155 $$\:{\mathbf{h}}_{k}\left(t\right)={\alpha\:}_{k}{\mathbf{h}}_{k}(t-1)+\sqrt{1-{\alpha\:}_{k}^{2}}{\hspace{0.17em}}{\mathbf{e}}_{k}\left(t\right)$$

where $\:{\alpha\:}_{k}$denotes the temporal correlation coefficient,, $\:{\mathbf{e}}_{k}\left(t\right)\sim\:\mathcal{C}\mathcal{N}(0,\mathbf{I})$ represents the innovation noise, and $\:{\mathbf{h}}_{k}(t-1)$ is the previously estimated channel. The temporal correlation coefficient is determined by the Doppler frequency according to

156 $$\:{\alpha\:}_{k}={J}_{0}\left(2\pi\:{f}_{D,k}{T}_{s}\right)$$

where $\:{J}_{0}(\cdot\:)$ is the zeroth-order Bessel function of the first kind, $\:{T}_{s}$ is the symbol duration, $\:{f}_{D,k}=\frac{{v}_{k}{f}_{c}}{c}$ is the Doppler frequency, $\:{v}_{k}$ is the velocity of the $\:k$-th user, $\:{f}_{c}\:$ is the carrier frequency, $\:c\:$ is the speed of light.

From a system design perspective, these results provide useful insights for operating under high-mobility conditions. In particular, the noticeable performance degradation due to CSI aging suggests that more frequent channel updates or even predictive tracking methods may be necessary to maintain reliable performance. In addition, the results show that ZF detection is more robust than MRC in high-mobility scenarios, which makes it a more suitable choice despite its higher complexity. While increasing the number of BS antennas generally improves the sum SE, it also makes the system more sensitive to outdated CSI, highlighting a trade-off between performance gains and channel tracking overhead. Overall, these observations indicate the importance of adopting adaptive and mobility-aware design strategies to ensure stable performance in high-speed environments such as V2X systems. Moreover, the effectiveness of spatial Sigma–Delta ADCs may depend on the spatial stationarity of the channel, which can be disrupted under high mobility, motivating further investigation into mobility-resilient quantization strategies.

Based on the results obtained, some practical design insights can be drawn. A moderate group size (typically in the range of 2–4 users per group) achieves a favorable balance between performance and computational complexity in group SIC detection. In addition, low-resolution Sigma–Delta ADCs, especially with 2-bit quantization, are sufficient to achieve near-optimal performance in most considered scenarios, offering a good trade-off between energy efficiency and system performance. These findings provide useful guidelines for the practical implementation of massive MIMO-NOMA systems.

## Conclusions

This paper analyzes the performance of massive MIMO–NOMA systems with MRC-GSIC and ZF-GSIC receivers using low-resolution spatial ΣΔ-ADCs over both Rician and Rayleigh fading channels. The derived asymptotic SINR expressions under imperfect CSI accurately capture system behavior for different numbers of antennas, ADC resolutions, and Rician K-factors. Results demonstrate that Sigma–Delta ADCs can effectively acquire CSI, particularly in the presence of strong LoS paths, mitigating quantization-induced errors. As the number of BS antennas grows, the spectral efficiency (SE) of both receivers converges and becomes limited by ADC resolution, while ZF-GSIC generally outperforms MRC-GSIC at moderate antenna counts. Additionally, using moderate-resolution ADCs provides a favorable trade-off between energy efficiency (EE) and performance. From a practical design perspective, the results suggest that employing moderate group sizes (e.g., 2–4 users per group) in GSIC detection achieves an effective balance between performance and computational complexity. Moreover, low-resolution Sigma–Delta ADCs, particularly with 2-bit quantization, are sufficient to attain near-optimal performance in most considered scenarios, offering a compelling trade-off between power consumption and system efficiency. Overall, these findings highlight the feasibility and efficiency of ΣΔ quantization in practical massive MIMO–NOMA systems, offering valuable guidance for designing future energy- and spectrally-efficient wireless networks. For future work, we will explore robust or predictive CSI techniques to mitigate mobility-induced performance losses.

## Data Availability

The datasets generated and/or analyzed during the current study are available from the corresponding author **Samar I. Farghaly** on reasonable request.
